# Visualizing the GPCR Network: Classification and Evolution

**DOI:** 10.1038/s41598-017-15707-9

**Published:** 2017-11-14

**Authors:** Geng-Ming Hu, Te-Lun Mai, Chi-Ming Chen

**Affiliations:** 1 Department of Physics, National Taiwan Normal University, Taipei, Taiwan; 2 Genomics Research Center, Academia Sinica, Taipei, Taiwan

## Abstract

In this study, we delineate an unsupervised clustering algorithm, minimum span clustering (MSC), and apply it to detect G-protein coupled receptor (GPCR) sequences and to study the GPCR network using a base dataset of 2770 GPCR and 652 non-GPCR sequences. High detection accuracy can be achieved with a proper dataset. The clustering results of GPCRs derived from MSC show a strong correlation between their sequences and functions. By comparing our level 1 MSC results with the GPCRdb classification, the consistency is 87.9% for the fourth level of GPCRdb, 89.2% for the third level, 98.4% for the second level, and 100% for the top level (the lowest resolution level of GPCRdb). The MSC results of GPCRs can be well explained by estimating the selective pressure of GPCRs, as exemplified by investigating the largest two subfamilies, peptide receptors (PRs) and olfactory receptors (ORs), in class A GPCRs. PRs are decomposed into three groups due to a positive selective pressure, whilst ORs remain as a single group due to a negative selective pressure. Finally, we construct and compare phylogenetic trees using distance-based and character-based methods, a combination of which could convey more comprehensive information about the evolution of GPCRs.

## Introduction

In the post-genome era, the number of newly discovered protein sequences deposited into databases grows explosively^1^. Faced with the avalanche of new sequences, it has now become a challenging task to efficiently determine the structure of these protein sequences and to further characterize their functions. How can we deal with the thousands of sequences being determined and deposited, and synthesize them into useful knowledge to the biological complexity and human medicine? To extract the knowledge from huge experimental data, new computational methods are desired to go beyond the traditional sequence homology method and efficiently seek correlations between protein sequences for their evolution history, structure, and functions. Moreover, to reach a tight control of cellular processes, proteins often act in association with other proteins in a dynamic way^2^. Therefore, it is desirable to develop powerful methods for the study of protein networks.

G-protein-coupled receptors (GPCRs), also known as seven transmembrane (TM) domain receptors or heptahelical receptors, form the largest protein superfamily in mammalian genomes. They share a common counter-clockwise bundle structure of seven TM helices and associate with heterotrimeric guanine nucleotide-binding proteins (G proteins)^3^. The conformational changes of GPCRs upon ligand binding activate the associated G protein to initiate a series of biochemical reactions within the cell. These intracellular reactions regulate a wide variety of physiological functions, such as smell, taste, vision, secretion, neurotransmission, metabolism, cellular differentiation and growth, inflammatory and immune response^4–6^. Consequently, malfunction of GPCR signaling pathways can cause various diseases, including cancer, diabetes, obesity, inflammation, cardiac dysfunction, and central nervous system disorders. The clinical importance of GPCRs can be delineated by their pharmaceutical applications; approximately 40–50% of all drugs (including the majority of best-selling drugs on the market) target GPCRs^7^. However, it is also known that drugs have only been developed to affect a small number of the GPCRs. As only 10% of all GPCRs encoded in the human genome are targeted by current drugs, GPCRs continue to be the most popular target class for drug discovery^8,9^. Furthermore, a growing number of studies have linked the aberrant over-expression of GPCRs and their autocrine/paracrine activation by agonists to various types of human malignancies^1,8,10^. Previous experiments have demonstrated that many GPCRs could serve as biomarkers for the early diagnosis of cancer, and the pharmacological inhibition of GPCRs could interrupt cancer progression and metastasis. Therefore GPCRs could play a crucial role in the development of a mechanism-based strategy for cancer prevention and treatment.

Currently, a widely used classification system of GPCRs is the A-F system that is mainly based on their amino acid sequences and functional similarities (designed fingerprints of the seven characteristic GPCR hydrophobic domains)^11,12^. This system identifies six classes, labeled A-F, from GPCR sequences from both vertebrates and invertebrates. Class A, also known as the “rhodopsin-like family”, is the largest group of GPCRs, which includes hormones, neurotransmitters, and light receptors and accounts for around 80% of GPCRs. Structurally, class A GPCRs have seven TM helices, together with the eighth helix and apalmitoylated cysteine at the C terminal tail. Class B, also called “secretin receptor family”, contains around 70 receptors, which have seven TM helices and a long N-terminal domain of around 120 residues stabilized by disulfide bonds. Class C includes the metabotropic glutamate family, GABA receptors, calcium-sensing receptors, and taste receptors. These receptors are characterized by seven TM helices and a large extracellular N-terminal domain with approximately 600 residues to which ligands bind. This clam-shaped domain is connected to the TM helix 1 by a cysteine-rich loop. Class D includes fungal mating pheromone receptors, class E includes cAMP receptors, and class F includes frizzled/smoothened receptors. The amino acid sequences of GPCRs in classes D-F also contain seven hydrophobic domains that are considered TM helices. Another classification system of GPCRs, called “GRAFS”, has also been proposed based on the phylogenetic tree of approximately 800 human GPCR sequences^13^. This system contains five main families named Glutamate (G), Rhodopsin (R), Adhesion (A), Frizzled/Taste2 (F), and Secretin (S). The main difference between the GRAFS system and the A-F system is the further division of class B into the Secretin family and the Adhesion family in the GRAFS system based on a preliminary finding that the evolutionary history of these two families is distinct from each other.

Due to the abundance of GPCRs and their diverse roles in cellular regulation and signal transduction, proper identification and classification of GPCRs are very helpful in their biological understanding and pharmaceutical applications^14,15^. Various methods have been proposed to identify GPCR sequences, such as alignment-free methods^16–18^ and statistical model methods^19–21^. To further classify identified GPCRs, hierarchical classifications of GPCRs have been developed. A straightforward strategy to classify proteins is mainly based on the sequence similarity searches using database search tools (e.g. BLAST, FASTA). Due to the lack of clear correlation between ligand specificity and sequence similarity, straightforward database search methods often fail to provide a proper classification scheme of GPCRs. Recently, we have developed the minimum span clustering (MSC) algorithm^22^ to classify proteins based on their sequence similarity. MSC is an efficient and unsupervised clustering algorithm, which performs automatic clustering at various resolution levels on the basis of the intrinsic properties of the data. This algorithm has successfully demonstrated the sequence-structure-function relationship for membrane proteins^23^ and enzymes^24^ that have known structures. For example, there is a high consistency of 0.86 between the MSC clustering of predicted protease sequences and the MEROPS functional classification. Visualization of the network structure of proteins has been realized by integrating MSC with the minimum spanning tree method^25^. Alternatively, instead of querying against complete sequences, search against motif databases have been implemented using regular expressions (PROSITE^26^), position-specific scoring matrices (BLOCKS^27^), frequency matrices (PRINTS^28^) and profile hidden Markov models (HMM) (Pfam^29^). Another strategy for protein classification is based on machine learning methods, such as support vector machines^30,31^, nearest neighbor^32^, artificial neural networks^33^, random forest^34^, logistic regression^35^, and covariant discriminant^36^. Other alignment-free methods, such as GPCR Tree^37^, 7TMRmine^38^, and the proteochemometric approach^39^, have also been applied to classify GPCRs.

The aim of the present work is to develop an automatic clustering system of GPCRs based on their sequence information, which can be used to visualize and delineate the relationship among GPCRs. In this study, we consider a protein network of mixed GPCRs and non-GPCR proteins, which is characterized by a distance matrix derived from the BLAST *E*-value of protein pairs to describe their sequence similarity. Based on the distance matrix, we apply the MSC method to cluster proteins in the database and distinguish GPCR clusters from non-GPCR clusters. The network structure of GPCR clusters is established and visualized by integrating MSC and minimum spanning tree. To interpret the observed network properties, such as the difference in the network structure of peptide receptors (PRs) and olfactory receptors (ORs), we calculate their *d*_N_/*d*_S_ ratio using the Nei-Gojobori method^40^ to reflect the selective pressure at the sequence level for these receptor subfamilies. Our results show a larger *d*_N_/*d*_S_ ratio for the PR subfamily, and therefore evolutionary change drives PRs to constitute a more diverse and distributed network structure, unlike a more aggregated structure of ORs, as seen in the minimum spanning tree diagram of GPCRs. Finally, we apply both distance-based and character-based methods to derive and compare the phylogenetic tree diagrams of GPCRs.

## Materials and Methods

### Dataset preparation

In this study, we study the detection and clustering of GPCRs by considering a base dataset of 2770 GPCR and 652 non-GPCR sequences. Among the 38525 GPCR sequences (in March 2015) in the GPCRdb (http://gpcrdb.org), only 2770 sequences that have been reviewed by Swiss-Prot were retrieved from GPCRdb as the target dataset. These GPCR sequences are from diverse organisms (more than 300 organisms) and contain both orthologs and paralogs^41^. We also consider simpler GPCR datasets of Homo sapiens (804 sequences), Mus musculus (488 sequences), and Rattus norvegicus (314 sequences) extracted from the target dataset, and the results of analyzing these three datasets are presented in the Supporting Information. The GPCRdb is a worldwide repository for GPCR-related data, which has the largest collections of receptor mutants and reference sequence alignments^42^. GPCRdb defines the classifications chemically (i.e., according to which ligands the receptor binds) based on the experimental data. Since protein-ligand interaction information has provided new ways of investigating protein functions, the GPCRdb classification can be viewed as a functional classification of GPCRs. As said by the classification of GPCRdb, our target dataset consists of 2305 class A receptors (rhodopsin-like), 182 class B receptors (secretin-like), 68 class C receptors (metabotropic glutamate/pheromone), 39 class D receptors (vomeronasal), 172 type 2 taste receptors (T2R), and 4 class E receptors (cAMP). The retrieved GPCRdb classification on 2015/03/09 is an earlier version, which is preferred over the newest version recently available. For the purpose of comparing the GPCRdb’s classification of GPCRs with the automatic clustering of MSC, no class F (frizzled) receptors are considered in this study. In addition, 652 non-GPCR membrane protein (MP) sequences (170 peripheral MPs and 482 TM MPs) are downloaded from PDBTM (http://pdbtm.enzim.hu) and added to the base dataset. The base dataset is classified by the multi-level MSC method to analyze the network structure of GPCRs and their differences from non-GPCR sequences. The detailed information regarding GPCR sequences in our target dataset is available in the Supporting Information Table S1. To test MSC for detecting GPCR sequences, we constructed a test dataset of 34 GPCRs (new depositions in Swiss-Prot after March 2015) and 20 non-GPCRs. The detailed information regarding sequences in our test dataset is available in the Supporting Information Table S2. We note that soluble protein sequences are not included in our test dataset since it is easy to distinguish between them and GPCRs.

To understand the MSC clustering of GPCRs, we further estimate the selective pressure for each GPCR subfamily by calculating the *d*_N_/*d*_S_ ratio, where *d*_N_ is the number of nonsynonymous substitutions per nonsynonymous site and *d*_S_ is the number of synonymous substitutions per synonymous site. For 2050 out of 2770 GPCRs in our target database, their corresponding gene sequences can be found from the KEGG database (http://www.genome.jp/kegg/) and used in estimating synonymous and nonsynonymous substitution rates by the Nei-Gojobori approximate method.

### Sequence distance calculation

The BLAST *E*-value is a parameter to describe the number of hits one expected to see just by chance when searching the best-matched region between sequences in a database of a particular size. As a lower *E*-value infers a more significant match, we define the symmetrized sequence distance, ${d}_{i,j}$, between two protein sequences *i* and *j* as ${d}_{i,j}=\sqrt{{E}_{i,j}{E}_{j,i}}$, where *E*_*i*,*j*_ is computed by BLASTP (http://blast.ncbi.nlm.nih.gov/Blast.cgi) using the general scoring matrix BLOSUM62 with default parameters. The defined sequence distance was used to cluster GPCR sequences with the MSC method. We note that this definition of sequence distance based on the *E*-value is not unique, and other distance definitions have been shown previously to give good predictions in studying protein networks^23,24^. In general, a large BLAST *E*-value (>1) has little meaning and only those sequence pairs with a small *E*-value play a role in this study. In the MSC method, the finest level clustering starts with the shortest distance and terminates at a pre-calculated threshold distance. At higher clustering levels, each cluster obtained from the previous level was considered as a node in the protein network, and the node to node distance was defined to be the shortest distance between the two clusters for further clustering of the protein network.

### Minimum span clustering method

**Figure 1:**
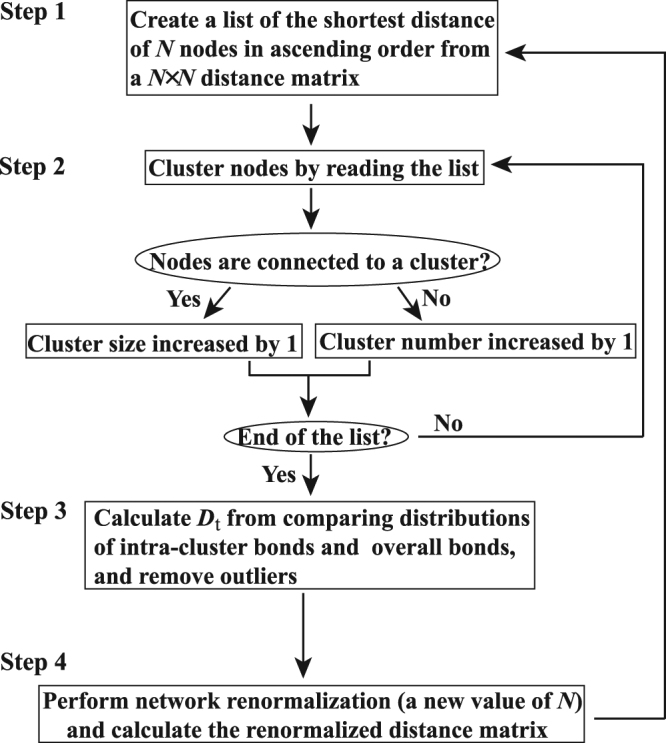
Flowchart of the unsupervised, multi-level MSC network clustering algorithm.

In this study, the MSC method was used to detect, cluster, and visualize the sequence similarity network of GPCRs from their sequence distance matrix by minimizing both the average intra-cluster distance of each cluster and the overall connected distance of the network. It provides a hierarchical approach to cluster and to visualize the structure of a complex network at various resolution levels. Here we briefly describe the four steps in the MSC procedure as in the flowchart of Fig. 1:

Step 1 (simplification). The MSC algorithm identifies the closest neighbor of each node and records their distances in a list of ascending order from the distance matrix. For a network of *N* nodes, instead of dealing with a distance matrix of *N* ^2^ elements, MSC only processes at most *N* distances in the list.

Step 2 (clustering). MSC starts to construct the first cluster by starting from the shortest node pair and then including additional pairs from the list in the order of increasing distance. For the added distance, if one of the two nodes is involved in one of the constructed clusters, the size of this cluster increases but the number of clusters remains the same. If both nodes of the distance are not involved in the above-constructed clusters, a new cluster is identified and the number of clusters increases. All clusters of the network are found when all distances in the list are considered. The identified clusters in the first run are referred as the first level clustering, which has the highest resolution.

**Figure 2:**
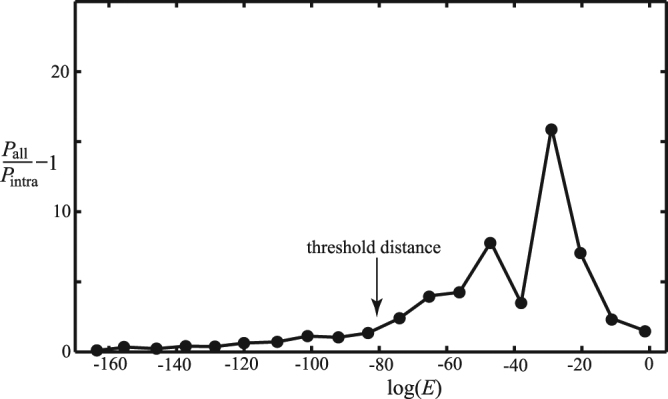
The relative difference in edge length distributions of the minimum spanning tree diagram and of the first level MSC clusters in the GPCR network. The length distribution of the minimum spanning tree includes both intra- and inter-cluster edges, whilst that of MSC clusters only includes intra-cluster edges. The difference is contributed from inter-cluster edges. The threshold distance between clusters is chosen to be 10^−80^, where the value of *P*_all_/*P*_intra_ −1 is nonzero and increases sharply.

Step 3 (truncation). For a minimum spanning tree diagram of the network, MSC calculates the threshold distance *D*_t_ by comparing the distributions of intra-cluster bonds (*P*_intra_) and overall bonds (*P*_all_). Since overall bonds of the network consist of intra- and inter-cluster bonds, the value of *P*_all_/*P*_intra_ is largely greater than 1 for the bond length greater than the threshold distance between clusters, which is considered as the definition of *D*_t_ in this study. For a node in a cluster predicted from step 2, if its shortest bond to the cluster members is longer than *D*_t_, this node will be considered as an outlier and removed from the cluster. The existence of outliers is often due to the fact that the dataset under investigation is incomplete. For the GPCR network, as shown in Fig. 2, the value of *D*_t_ is found to be 10^−80^ (where the value of *P*_all_/*P*_intra_ −1 is nonzero and increases sharply) and all intra-cluster bonds longer than 10^−80^ are truncated.

Step 4 (renormalization). Clusters constructed in step 2 are considered as renormalized nodes, and the distance between clusters is calculated by finding the shortest distance of inter-cluster node pairs between two clusters. The network consisting of these renormalized components is further clustered by steps 1–3, and higher levels of clustering with a lower resolution are constructed.

**Figure 3:**
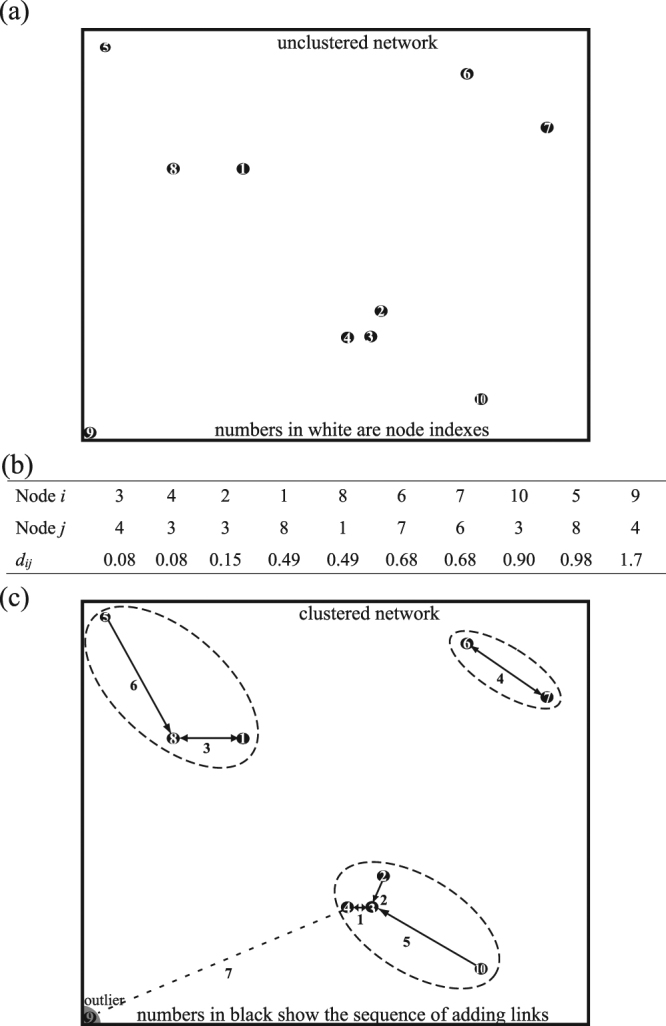
A demonstrated example of implementing MSC for a simple network of 10 nodes: (**a**) the un-clustered network, (**b**) the list of the shortest distance pairs for network nodes, and (**c**) the clustered network after implementing MSC.

An example of implementing MSC for a simple network of 10 nodes is shown in Fig. 3. For an un-clustered network shown in Fig. 3(a), the shortest distance list derived from step 1 of MSC is displayed in Fig. 3(b). In implementing step 2, the core of the first cluster contains nodes 3 and 4 (the shortest distance pair connected by a double-headed arrow). Node 2 is added to this cluster (indicated by a single-headed arrow) by considering the next distance in the list. The primary clustering is complete after all links in the list are considered. After step 2, as shown in Fig. 3(c), the network is decomposed into three MSC clusters, each of which is a minimum spanning tree diagram. In step 3, the threshold distance of clusters is calculated and assumed to be smaller than 1.7. All links longer than the threshold distance are broken (indicated by a dashed line), and isolated nodes (outliers) are removed from the network. In step 4, each cluster (enclosed by a dashed loop) is considered as a renormalized node in the next level MSC. In our previous test on the clustering of MPs, MSC can lead to more consistent clustering results (higher intra-cluster similarity and lower inter-cluster similarity) than Markov clustering algorithm (MCL), hierarchical clustering (HC), affinity propagation (AP) and K-means. The speed of MSC was also compared with that of various other clustering algorithms by running their Matlab codes on an Intel Core i7 desktop computer. For a network of 10,000 nodes, the clustering time is 0.1 second in MSC, which is significantly shorter than 18 seconds in HC, and 12+ hours in AP or *K*-means.

### Estimating synonymous and nonsynonymous substitution rates

In genetics, the ratio of *d*_N_/*d*_S_ is used to estimate the balance between neutral mutations (*d*_N_/*d*_S_ = 1), negative selection (*d*_N_/*d*_S_ < 1) and positive selection (*d*_N_/*d*_S_ > 1) acting on a set of homologous protein-coding genes. In the past two decades, several approximate methods have been suggested for this estimation, which share three basic steps including (1) counting the numbers of synonymous and nonsynonymous sites in the sequences, (2) counting the numbers of synonymous and nonsynonymous differences between the two sequences, and (3) calculating the numbers of synonymous (*d*_S_) and nonsynonymous (*d*_N_) substitutions per site between the two sequences with a correction for multiple substitutions at the same site. Here we use the method of Nei and Gojobori^40^, which appears to be most commonly used. Although the method gives no weights to different types of codon substitutions, computer simulation indicates that it tends to give an accurate estimation of *d*_S_ but an underestimation of *d*_N_. For a very large number of nucleotide substitutions per site, its accuracy in estimating both *d*_S_ and *d*_N_ deteriorates. The three basic steps of Nei-Gojobori method is described as the following:

Step 1. The number of synonymous (nonsynonymous) sites, *s*_*j*_ (*n*_*j*_), for the *j*-th codon of a gene sequence is given by *x*_j_ = Σ_*i*=1,2,3_ *f* ^*x*,*j*^_*i*_, where *x* = *s*, *n*, and *f* ^*x*,*j*^_*i*_ is the proportion of *x* changes at the *i*-th position of the codon. For a sequence of *r* codons, the total number of synonymous (nonsynonymous) sites, *S* (*N*) is given by *X* = Σ_*j*=1…*r*_ *x*_*j*_, where *X* = *S*, *N* and *N* + *S* = 3*r*.

Step 2. In this step, we calculate the number of synonymous and nonsynonymous changes between each pair of aligned sequences, codon-by-codon. When there is one (two, or three) nucleotide difference, there is one (two, or six) possible pathway between the codons. In these cases, the number of synonymous and nonsynonymous differences for the *j*-th codon, *s*_d,*j*_ and *n*_d,*j*_ respectively, can be calculated by considering all possible pathways. Summing for all codons, the total numbers of synonymous and nonsynonymous differences, *S*_d_ and *N*_d_ respectively, are given by *X*_d_ = Σ_*j*=1…*r*_ *x*_d,*j*_, where *X* = *S*, *N* and *x* = *s*, *n*.

Step 3. The proportions of synonymous (*p*_S_) and nonsynonymous (*p*_N_) differences are estimated by the equation *p*_X_ = *X*_d_/*X* with mean values taken over every pair-wise comparison (*X* = *S*, *N*). The numbers of synonymous (*d*_S_) and nonsynonymous (*d*_N_) substitutions per site are estimated using the Jukes-Cantor formula as *d*_X_ = −0.75 ln(1−4 *p*_X_/3).

## Results, Discussion and Applications

Detecting sequence similarities among proteins has been shown to be a reliable method for computational prediction of protein functions. Alternative strategies, such as fingerprints-based or support vector machine-based methods, have also been applied to search for novel GPCRs in protein sequence data. In this study, we attempted to use the MSC method for detecting and clustering GPCRs with the base dataset of mixed GPCR and non-GPCR sequences. To begin with, the base dataset was decomposed into clusters at various resolution levels and we analyzed the GPCR clusters and their differences from non-GPCR clusters. Based on the MSC analysis, we demonstrated a simple method to detect GPCR sequences from the test dataset. Furthermore, we discussed the clustering results of GPCRs at various resolution levels. In particular, we interpreted the observed difference in network structure between PRs and ORs by their d_N_/d_S_ ratio. Finally, we constructed and compared phylogenetic trees of GPCRs using both distance-based and character-based methods.

### MSC Clustering and GPCR Detection

**Figure 4:**
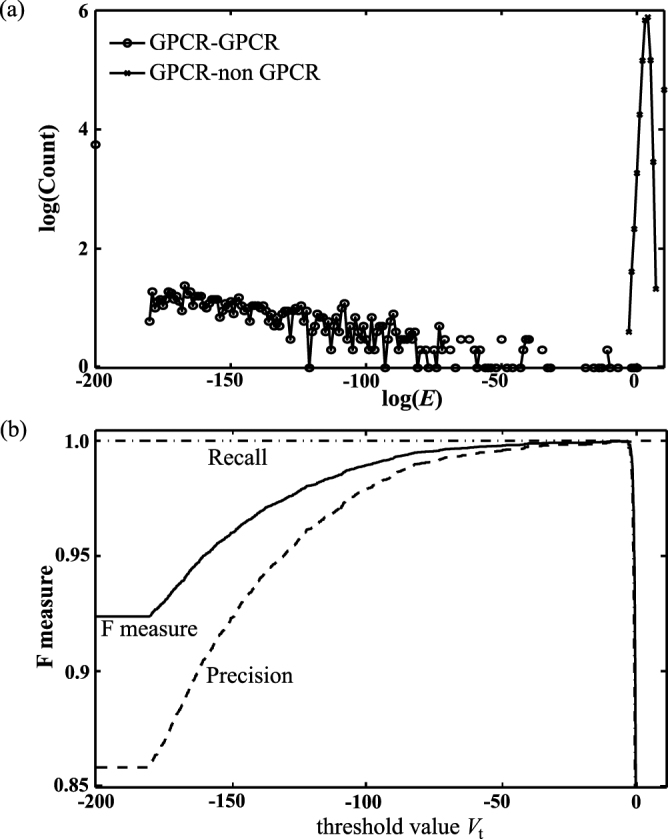
The distance distribution between GPCR and non-GPCR sequences and the minimum distance distribution of a GPCR sequence to other GPCR sequences in the base dataset (**a**), and the *F* measure for the detection of GPCR sequences based on the MSC results of the base dataset as a function of the threshold value *V*_t_ (**b**). Here the *F* measure is defined as *F* = 2·precision·recall/(precision + recall).

After implementing the MSC algorithm with a threshold distance of 10^−80^ for classifying the base dataset of protein sequences, as shown in the Supporting Information Table S1, the set of 3422 sequences was grouped into 763 clusters and 654 isolated nodes at the first (finest) resolution level. Here an isolated node is an outlier the distance of which to its closest neighbor is longer than 10^−80^. The existence of outliers is mainly due to limited dataset size (e.g., no family members of these outliers are included in the base dataset) and the number of outliers could be greatly reduced for a complete dataset. There is no mix-up of GPCR and non-GPCR sequences in the clusters predicted by MSC. In other words, the closest neighbor of a GPCR sequence is always another GPCR sequence. In Fig. 4(a), we display the distribution of the minimum distance of a GPCR sequence to other GPCR sequences and that of a GPCR sequence to non-GPCR sequences in the base dataset, which shows that the minimum distance of a GPCR to other GPCRs is in general much less than 0.0009, while that of a GPCR to non-GPCR sequences is greater than 0.0009. Since there are only three GPCR outliers whose minimum distance is greater than 0.0009, it is, therefore, feasible to use the MSC clustering of the target dataset of GPCRs as a tool to efficiently detect possible GPCR candidates from a large set of unknown sequences. In Fig. 4(b), we calculated the *F* measure (*F* = 2·precision·recall/(precision + recall)) for the detection of GPCR sequences with a threshold value *V*_t_ based on the MSC results of the base dataset. More specifically, for an arbitrary sequence whose minimum distance to the GPCR sequences in the target dataset is smaller than 10^*V*^_t_, it will be considered as a GPCR sequence. The *F* measure has a maximum value of 0.9998 if *V*_t_ is −3.05. Based on the MSC clustering results of the base dataset, we further examined the validity of GPCR detection with *V*_t_ = −3.05 for 54 test protein sequences (34 GPCRs and 20 non-GPCRs) as described in Dataset Preparation. As shown in the Supporting Information Table S2, there are only three GPCR sequences (G5ECQ2, Q8K4C8, and O45767) that were mistakenly identified (minimal log(*E*) = −1.7, −1.8 and −2.7). All these three sequences (G5ECQ2 and Q8K4C8 are frizzled receptors and O45767 is a pheromone receptor of Caenorhabditis elegans) are outliers, whose family members are not included in the base dataset. After enlarging the base dataset to include some of their family members, all test sequences are correctly identified.

### GPCR Clustering

**Figure 5:**
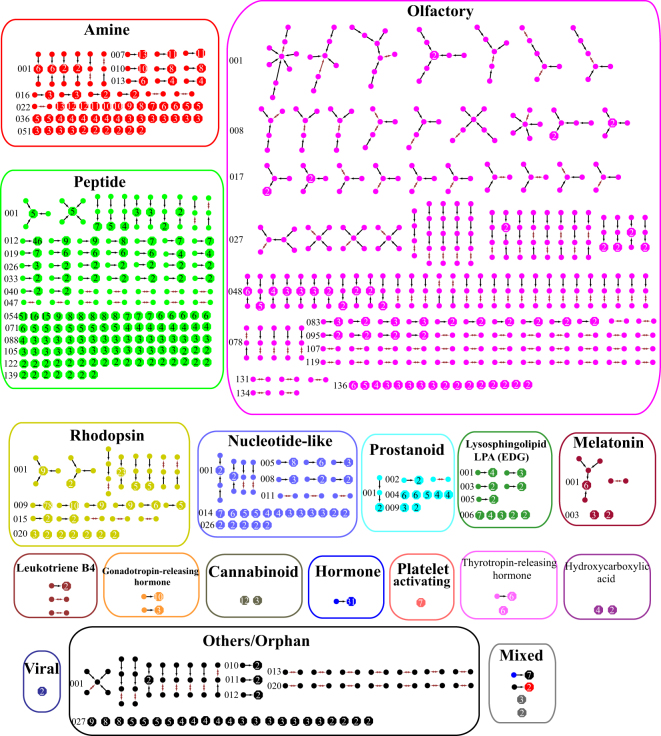
The minimum spanning tree diagrams of 503 level 1 MSC clusters in the rhodopsin-like class. Here each circle represents a GPCR sequence, and each hexagon represents a number of sequences whose mutual *E* value is zero (the number is shown in the hexagon). The color of circles or hexagons is chosen based on their functional classification in the GPCRdb.

For the set of 2770 GPCR sequences, it is decomposed into 620 clusters and 68 isolated nodes at the first MSC level. The 503 MSC clusters of the rhodopsin-like class (the largest family of GPCRs) are shown in Fig. 5, and those of other GPCR classes are shown in the Supporting Information Figure S1. Here each circle represents a GPCR sequence, and each hexagon represents a number of sequences whose mutual *E* value is zero (the number is shown in the hexagon). Thus each hexagon represents a collection of conserved protein sequences. Each node (circle or hexagon) is colored according to their functional classification in the GPCRdb. The color black is reserved for GPCRs labeled orphan/others, while the color grey is used for hexagons containing sequences with different colors. Moreover, the double-headed arrow signs show the core of MSC clusters (the pair of sequences with the shortest distance), and the arrow signs show the direction that additional sequences were added to the cluster. Each cluster is labeled by the first two alphabets of the receptor group and a three-digit number. For example, the first cluster in the amine receptor subfamily is labeled as Am-001. Overall, the first level MSC clustering of GPCRs has a small mean intra-cluster distance of 2.5 × 10^−61^ and a large mean inter-cluster distance of 1.7 × 10^3^, indicating that MSC groups similar sequences together and separates dissimilar sequences. By comparing level 1 MSC results with the classification of GPCRdb, the consistency is 87.9% for the fourth level of GPCRdb, 89.2% for the third level, 98.4% for the second level, and 100% for the top level (the lowest resolution level in GPCRdb). For rhodopsin-like receptors, 500 out of 503 MSC predicted clusters (99.4%) are consistent with the second level classification of GPCRdb. Here the consistency is calculated as the percentage of MSC clusters whose members belong to the same GPCRdb classification group. A detailed comparison of MSC and GPCRdb classifications is displayed in the Supporting Information Table S1.

### Minimum Spanning Tree Diagram of GPCR Clusters

**Figure 6:**
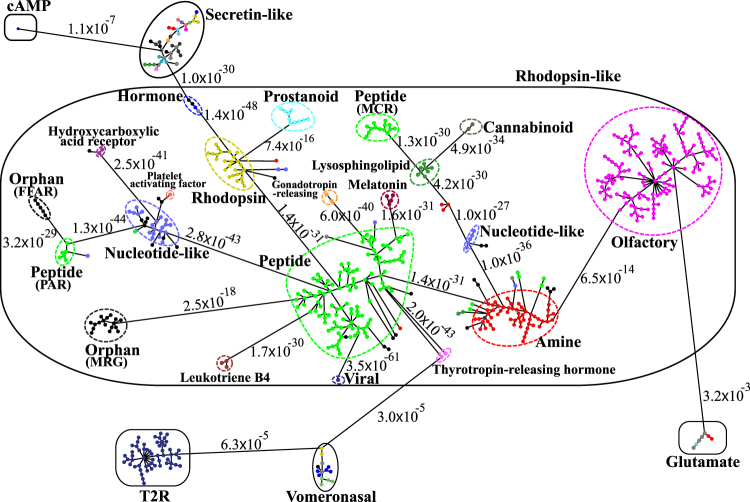
The minimum spanning tree diagram of the 620 level 1 MSC clusters for the GPCR network in the target dataset. Here each circle represents an MSC cluster the color of which is according to the color scheme in Figs 5 and S1. The length of edges is not proportional to their distance, but the distances between subfamilies and classes are labeled to see their sequence similarity.

To further illustrate the network structure of GPCR proteins, in Fig. 6, we display the minimum spanning tree diagram of the 620 MSC clusters. Here each cluster is coarse-grained as a node and represented by a filled circle. Each circle is colored according to the function of the corresponding MSC cluster. For the sake of clarity, the length of edges is not proportional to their distance due to heterogeneous distance distribution. However, the distances between subfamilies and classes are labeled to see their sequence similarity. Additional information for Fig. 6, such as the distribution of sequences from various organisms in each MSC cluster and the average percentage of paralog pairs for various functional clusters, can be found in the Supporting Information Figure S2. As shown in Fig. 6, MSC clusters of the same color tend to aggregate together and constitute a receptor subfamily (enclosed by a dashed loop). These receptor subfamilies can be grouped into 6 GPCR classes (rhodopsin-like, secretin-like, glutamate, vomeronasal, cAMP, and T2R) as seen from those families enclosed by a solid loop. The sequence clustering of MSC is generally consistent with the functional classification of GPCRdb. For example, most GPCR clusters (503/620) are rhodopsin-like. For taste receptors, T1R belongs to class C (glutamate), while T2R is a putative GPCR family (T2R is distantly related to class A in the A-F classification, but is a distinct cluster within the frizzled/taste 2 family in the GRAFS classification). Therefore, although GPCR sequences within these classes can share less than 25% identity between species^43^, information derived from their sequence alone (without knowing which ligand they bind) could still be very useful in deciphering their functions and predicting the functions of novel sequences. In general, we have intra-subfamily distances $\ll$inter-subfamily distances $\ll$inter-class distances. Most inter-class distances are greater than 10^−7^, whereas inter-subfamily distances are smaller than 10^−13^. However, the minimum distance of 10^−30^ between the Flamingo proteins (secretin-like) and the hormone receptors (rhodopsin-like) is considerably smaller than that of 10^−13^ between olfactory receptors and amine receptors in the rhodopsin-like class. The hormone receptor group contains LGR (leucine-rich repeat-containing GPCRs)-like receptors (colored in black for types 4 & 5, and colored in grey for types 7 & 8 mixed with a gonadotropin receptor sequence) and glycoprotein hormone receptors (colored in blue). Phylogenetic analysis showed that sea anemone LGR shares a close relatedness to mammalian glycoprotein hormone receptors^44^, which is consistent with our sequence similarity analysis. On the other hand, Flamingo proteins have large ectodomains containing cadherin repeats and seven-pass TM regions similar to peptide hormone receptors. The hormone receptor motif (HormR domain) of Flamingo proteins might participate in hormone interactions and requires further investigation^45^. The general network structure of GPCRs in the target dataset of 300 + organisms observed in Fig. 6 has also been observed in GPCR networks of a single organism. For the three simpler GPCR datasets, MSC finds 197 clusters for Homo sapiens, 110 clusters for Mus musculus, and 71 clusters for Rattus norvegicus at the first resolution level. In the Supporting Information Figure S3, we present the minimum spanning tree diagrams of level 1 MSC clusters for Homo sapiens (a), Mus musculus (b), and Rattus norvegicus (c). There is a close similarity in the relationship between GPCR functional clusters of the target dataset and that of the Homo sapiens dataset, except that T2R is closer to OR in Figure S3(a) for the Homo sapiens dataset. There is a slightly larger discrepancy in the cluster relationship between Fig. 6 and Figure S3(b, c). The cluster relationship between the Mus musculus and Rattus norvegicus datasets is also in close similarity, except that PR is closer to OR in Figure S3(c) for the Rattus norvegicus dataset.

**Figure 7:**
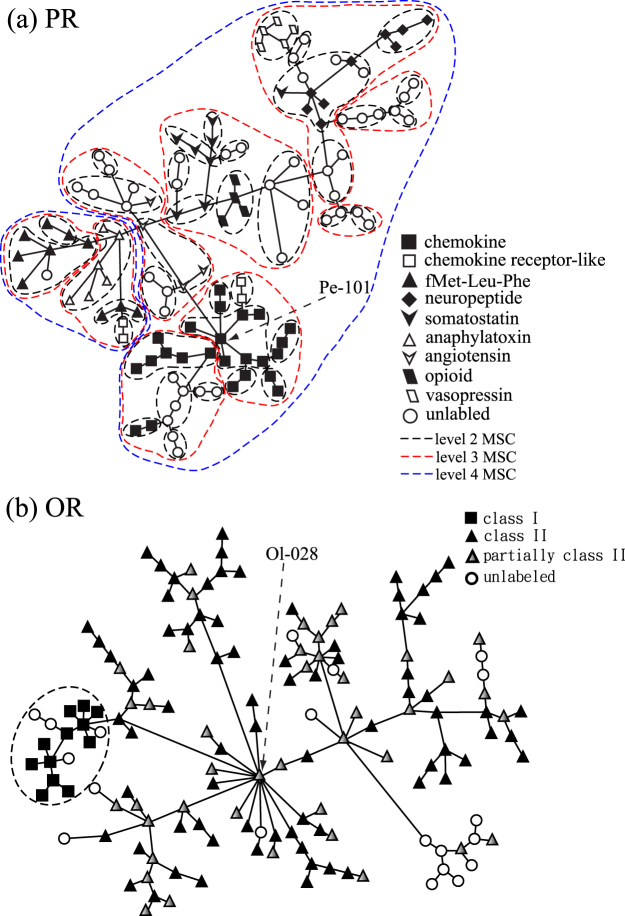
The minimum spanning tree diagrams of PRs’ main group (**a**) and ORs (**b**) in the target dataset. Here each node represents a level 1 MSC cluster. In (**a**), the shape of nodes designates which ligand they bind. Dashed loops in black, red, or blue show the MSC clustering of PRs at level 2, level 3, or level 4, respectively. In (**b**), the shape of nodes labels receptor clusters belonging to class I, class II, partially class II, or others.

For rhodopsin-like GPCRs, the largest two subfamilies are PRs and ORs. PRs constitute a large group of GPCRs that are activated by extracellular protein or peptide ligands; ORs are expressed in the cell membranes of olfactory sensory neurons and are responsible for detecting odorants, which give rise to the sense of smell. From the minimum spanning tree diagram in Fig. 6, the PR subfamily is clearly the hub of class A, and closely resembles other subfamilies in sequence. It is found that PRs are decomposed into three groups and several nodes connected to the amine subfamily. The two separate PR groups are the melanocortin receptor (MCR) group and the protease-activated receptor (PAR) group. For the main group of PRs, as shown in Fig. 7(a), we label the level 1 MSC clusters according to their ligands for the largest nine types. Clusters of all other types are not distinguished and are represented by open circles. Clusters of the same ligand type tend to aggregate together, suggesting a close sequence-function relationship of GPCRs. Here we use the clustering results of PRs in Fig. 7(a) as an example to further illustrate the multi-level MSC method. At the second level MSC, these clusters of similar functions are further clustered into the same group (level 2 clusters), as seen from those nodes enclosed by black dashed loops. At the third level MSC, level 2 clusters further aggregate to constitute level 3 clusters (red dashed loops). At this level, most GPCRs with the same ligand type can be found in the same cluster, except for chemokine receptors and fMet-Leu-Phe receptors. At the fourth level MSC, the main peptide receptor group is decomposed into two level 4 clusters (blue dashed loops). It is found that the chemokine receptor-like sequences (open squares) are distributed in two different level 4 clusters, suggesting that their sequence dissimilarity is larger than that of other PR sequences. Within the PR subfamily, the most connected hub (degree of the node is 7) indicated by a dashed arrow is the MSC cluster Pe-101 (C-C chemokine receptor type 4), which is surrounded by other types of chemokine receptors.

On the other hand, the OR subfamily is more remotely connected and less similar to other subfamilies in class A. The closest neighbor of the OR subfamily in class A is the amine receptor subfamily (which is understandable as trace amine receptors function as olfactory receptors for volatile amines in vertebrates^46,47^) and their minimum distance is of the order of 10^−13^. Unlike PRs, all ORs constitute a single group. As displayed in Fig. 7(b), we label both class I (filled squares for fish-like receptor clans: 51, 52, 55, 56) and class II (filled triangles for tetrapod specific receptor clans: 1–13) ORs on the basis of evolutionary divergence^48,49^. All other OR clusters are not labeled and are represented by open circles. For those clusters consisting of both class II and unlabeled ORs, they are represented by partially filled triangles. It is found that all class I ORs (in the target dataset) constitute a small subgroup while class II ORs are widely spread. Within the OR subfamily, the most connected hub as indicated by a dashed arrow is the level 1 MSC cluster Ol-028 the degree of which is 14.

**Figure 8:**
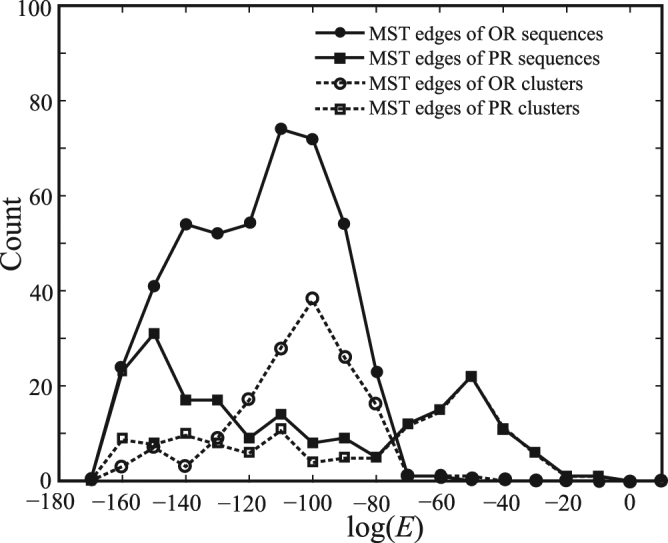
The distributions of sequence-sequence and cluster-cluster distances for PRs and ORs in a minimum spanning tree diagram.

In Fig. 8, we display the distributions of sequence-sequence and cluster-cluster distances for PRs and ORs in a minimum spanning tree diagram. For PRs, the distribution of sequence-sequence distances has three well-separated peaks: The first peak positioned at *E* = 0 (count = 478, not shown in Fig. 8 for clarity) is contributed from those hexagon nodes in Fig. 5; the second peak positioned at log(*E*) = −150 is the intra-cluster sequence-sequence distribution; the third peak positioned at log(*E*) = −50 is the inter-cluster sequence-sequence distribution. The above interpretation makes sense as the third peak of the sequence-sequence distribution overlaps with that of the cluster-cluster distribution. For ORs, there are also three peaks for the distribution of sequence-sequence distances and one peak for the distribution of cluster-cluster distances. Again, for clarity, we do not show the first peak positioned at *E* = 0 (count = 80). As the second peak of the sequence-sequence distribution is much higher than that of the cluster-cluster distribution, it is apparent that a significant portion of the intra-cluster sequence-sequence distances is in the range of the second peak. Therefore we conclude that sequences of PR clusters are more similar to each other than sequences of OR clusters.

### Evolution of GPCRs

**Figure 9:**
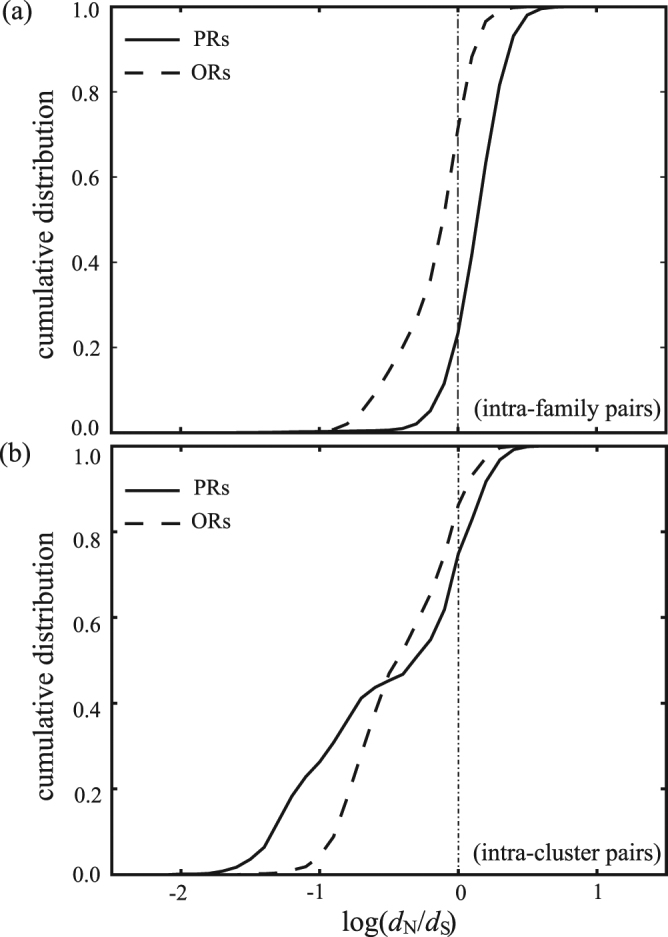
Cumulative distribution of the *d*_N_/*d*_S_ ratio for intra-subfamily sequence pairs (**a**) and for intra-cluster sequence pairs (**b**) of PRs and ORs. Negative selection is implied for *d*_N_/*d*_S_ < 1, while positive selection is implied for *d*_N_/*d*_S_ > 1.

To further understand the clustering results of PRs and ORs, in Fig. 9, we calculate the cumulative distribution of the *d*_N_/*d*_S_ ratio for intra-subfamily sequence pairs (a) and for intra-cluster sequence pairs (b). In Fig. 9(a), all sequence pairs in the PR/OR subfamilies are considered in calculating the ratio of *d*_N_/*d*_S_. For PRs, the cumulative distribution is 0.23 for negative selection (*d*_N_/*d*_S_ < 1) and 0.67 for positive selection (*d*_N_/*d*_S_ > 1). For ORs, the cumulative distribution is 0.74 for negative selection and 0.26 for positive selection. Clearly, the selective pressure tends to further diversify PR sequences but not OR sequences. This is consistent with our MSC clustering results in Fig. 6 that the PR subfamily is decomposed into three separate groups and the OR subfamily remains as one group consisting both classes I and II receptors. In Fig. 9(b), only sequence pairs in each MSC cluster (level 1) of PR/OR subfamilies are considered in calculating the ratio of *d*_N_/*d*_S_. For intra-cluster pairs, it is found that both PRs and ORs have a large cumulative distribution for negative selection (0.87 for ORs, and 0.75 for PRs), suggesting that most mutations are nonsynonymous substitutions. However, PRs have a higher cumulative distribution initially (up to *d*_N_/*d*_S_ = 0.32) reflecting the fact that a large number of sequence pairs have *E* = 0 in the PR subfamily. The ratio of *d*_N_/*d*_S_ for those hexagons in Fig. 5 is much less than 1, suggesting a negative selection for these conservative clusters of proteins.

### Comparing Phylogenetic Trees of GPCRs

Phylogenetic trees composed of a number of sequences can be constructed by distance-based methods or character-based methods^50^. Distance-based methods, such as neighbor-joining (NJ)^51^, multi-dimensional scaling (MDS)^52^ or minimum spanning tree, depend on a measure of genetic distance between sequences and construct a phylogenetic tree by linking closely related sequences together. Character-based methods, such as maximum likelihood or Bayesian inference, simultaneously compare all sequences in the alignment, calculate a score for each tree by considering one character at a time, and optimize the score to derive a phylogenetic tree (by an exhaustive search for small datasets or by a heuristic search for large datasets). In general, the trees derived from all available methods are un-rooted; rooting is performed after the analysis. The main difference between character-based and distance-based methods is that the former fit individual characters to the tree, whereas the latter fit all the characters at once.

**Figure 10:**
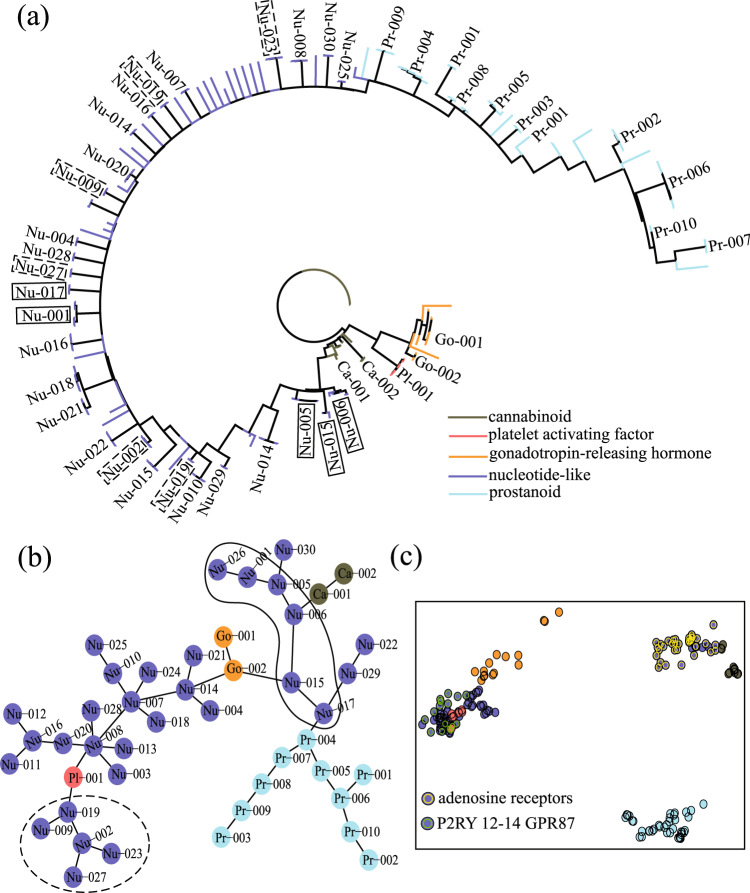
Phylogenetic trees of 185 GPCR sequences using both character-based (**a**) and distance-based methods (**b**). The data points in (**c**) show the location of these sequences on a two-dimensional projection by MDS. The branching tree diagram in (**a**) was constructed using the maximum likelihood method and displayed with the polar tree layout. The minimum spanning tree diagram in (**b**) was displayed by viewing each level 1 MSC cluster as a node.

To improve our understanding of the molecular evolution of GPCRs and the phylogenetic analysis, we construct phylogenetic trees of GPCRs using both distance-based and character-based methods and compare the general structure of these trees. For the distance-based method, we use the minimum spanning tree algorithm to derive the general tree structure of GPCRs. The tree diagram is further simplified using the MSC algorithm by representing each level 1 MSC cluster as a node. For the character-based method, we use ProtTest 3 to implement a parallel search for the maximum likelihood estimation of model parameters and phylogenetic trees^53^. For the purpose of demonstration, the phylogenetic trees composed of 185 sequences (45 level 1 MSC clusters) of nucleotide-like receptors, prostanoid receptors, gonadotropin-releasing receptors, cannabinoid receptors, and platelet activating receptors are displayed in Fig. 10: (a) the polar tree layout of the phylogenetic tree based on the maximum likelihood method, (b) the coarse-grained minimum spanning tree diagram, and (c) the two-dimensional projection of sequence location by MDS. In Fig. 10(a), if all sequences of a tree branch belong to the same MSC cluster, this branch will be noted by the corresponding cluster label; otherwise, the branch will not be labeled. In general, sequences of the same function tend to aggregate together in the two tree diagrams. Furthermore, sequences in the same MSC cluster (level 1) are often located in close proximity. Therefore both tree diagrams predict a similar relationship among sequences within level 1 MSC clusters. However, after renormalizing each cluster as a node, the two tree diagrams predict significantly different locations of these nodes. Thus these two trees predict different evolutionary relationships between clusters. To further elaborate this observation, we discuss two subgroups of nucleotide-like receptors enclosed by dashed and solid loops in Fig. 10. In Fig. 10(b), nodes enclosed by the dashed loop belong to the subfamily of purinoceptor: P2RY12–14 GPR87 (UDP-glucose), while nodes enclosed by the solid loop belong to the subfamily adenosine receptors. For comparison, in Fig. 10(a), these clusters are enclosed by dashed rectangles (P2RY 12-14 GPR87) or solid rectangles (adenosine receptors). In contrary to the aggregation of clusters of the same subfamily in Fig. 10(b), it is found that these clusters spread widely in the phylogenetic tree of Fig. 10(a). In general, we believe that predictions from distance-based methods are more consistent with the functional classification of GPCRdb than those from character-based methods. The two-dimensional projection of sequence locations by MDS in Fig. 10(c) basically gives the same prediction as that in Fig. 10(b), except that a higher dimensional projection is needed for the clustering of platelet-activating factor sequences. We note that the execution time for running MDS in (c) is substantially (about 200 times) longer than that in (b). We also note that, as shown in the Supporting Information Figure S4, the clustering of 185 sequences in the target dataset (a) and that of 804 sequences in the Homo sapiens dataset (b) by the NJ method are inconsistent with the GPCRdb classification. Finally, we investigate the structural robustness of these two trees upon sequence deletion. After randomly removing one sequence from the datasets, the resulted trees are only locally altered. The general structures observed in the two trees of Fig. 10 are not affected by random sequence deletion.

## Conclusion

In this study, we applied the MSC method to classify the sequence similarity network of proteins with a base dataset of 2770 GPCR and 652 non-GPCR sequences. MSC is an efficient and unsupervised clustering algorithm, which performs automatic clustering at various resolution levels on the basis of the intrinsic properties of the data. The purpose of this study is to demonstrate MSC as an efficient method for detecting GPCR sequences and systematically classifying the GPCR network. Our results suggest a significant difference in sequence between GPCR and non-GPCR proteins, and MSC is able to detect GPCR sequences with high accuracy if the base dataset is properly chosen. The clustering of 2770 GPCR sequences was performed at various resolution levels of MSC. The consistency between level 1 MSC clustering and the GPCRdb classification is between 88% (for the fourth GPCRdb level) and 100% (for the top GPCRdb level). To visualize the general structure of the GPCR network, we displayed a minimum spanning tree diagram for the level 1 MSC clusters of the network, which is meaningfully consistent with the functional classification of GPCRdb. Further explorations of the GPCR network were demonstrated by investigating the largest two subfamilies, PRs and ORs, in class A for their network structure and molecular evolution. Our calculations on the selective pressure (ratio of *d*_N_/*d*_S_) of PRs and ORs explain the observed MSC clustering structures of these protein subfamilies in the minimum spanning tree diagram: Overall ORs are subject to negative selection, while PRs are subject to positive selection; however, in many MSC clusters of PRs (hexagons in Fig. 5), these sequences are under large negative selective pressure and are conservative. Finally, we have constructed and compared phylogenetic trees of a test set of 185 GPCR sequences using both distance-based (minimum spanning tree) and character-based (maximum likelihood) methods. In general, branches or nodes in these trees are both consistent with our level 1 MSC results and functionalities of GPCRs. In other words, molecular evolution predicted by these two trees correlates strongly with molecular functions. At a higher level of GPCR functionalities, nodes of a similar functionality group together in the minimum spanning tree, while branches of similar functionalities spread widely in the phylogenetic tree based on maximum likelihood. In addition, branching tree diagrams constructed by maximum likelihood do not take into account adaptive radiation that might occur in the case of rapid diversification, whereas minimum spanning trees could contain hub nodes which link to a large number of similar sequence patterns. Since no computational phylogenetic methods are likely to perfectly replicate the evolutionary tree that represents the historical relationships between the species being analyzed, we believe that a combination of distance-based and character-based tree diagrams could convey a more comprehensive evolution picture of the system.

GPCRs are crucial gatekeepers for eukaryotic signal transduction, which represent the largest protein family in the human proteome. They recognize an astounding variety of extracellular stimuli and initiate cascades of cellular and physiological responses through coupling to various intracellular proteins. Understanding the structure, function, and evolution of GPCRs is valuable to basic research in cell signaling and molecular recognition, as well as to applied research in drug discovery and disease treatment. We believe that this study provides a panoramic view of the GPCR network. Currently, an interactive website for understanding the GPCR network is under construction, which will better assist readers to visualize the relationship among GPCRs at various resolutions through the multi-level MSC.

## Electronic supplementary material

Supporting Information
