# Supplementary material for: Visualizing the GPCR Network: Classification and Evolution
Source: Sci Rep. 2017 Nov 14;7:15495. doi: 10.1038/s41598-017-15707-9 (PMC5686146; doi:10.1038/s41598-017-15707-9)
Supplement: Supplementary file 1 — Supporting Information [file 41598_2017_15707_MOESM1_ESM.pdf]

Supporting Information

For **"Visualizing the GPCR Network: Classification and Evolution"**

by Geng-Ming Hu, Te-Lun Mai, and Chi-Ming Chen

Table of Contents

1. Supplementary Figures S1-S4 discussed in the paper (pages 1-4).
2. Supplementary Tables of the target dataset (S1) and the test dataset (S2) (pages 5-68).

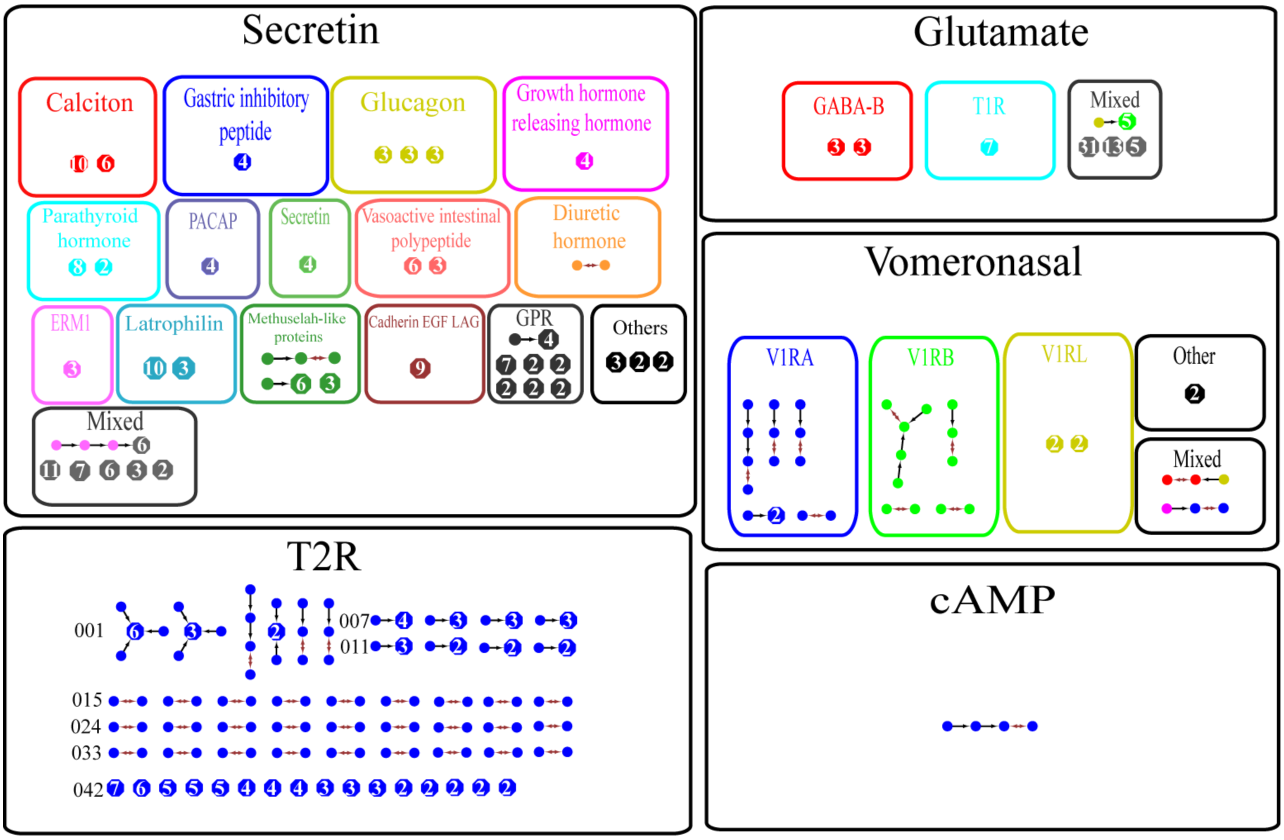

Figure S1. The minimum spanning tree diagrams of 117 level 1 MSC clusters in the target dataset (except for the rhodopsin-like class). Here each circle represents a GPCR sequence, and each hexagon represents a number of sequences whose mutual *E* value is zero (the number is shown in the hexagon). The color of circles or hexagons is chosen based on their functional classification in the GPCRdb.

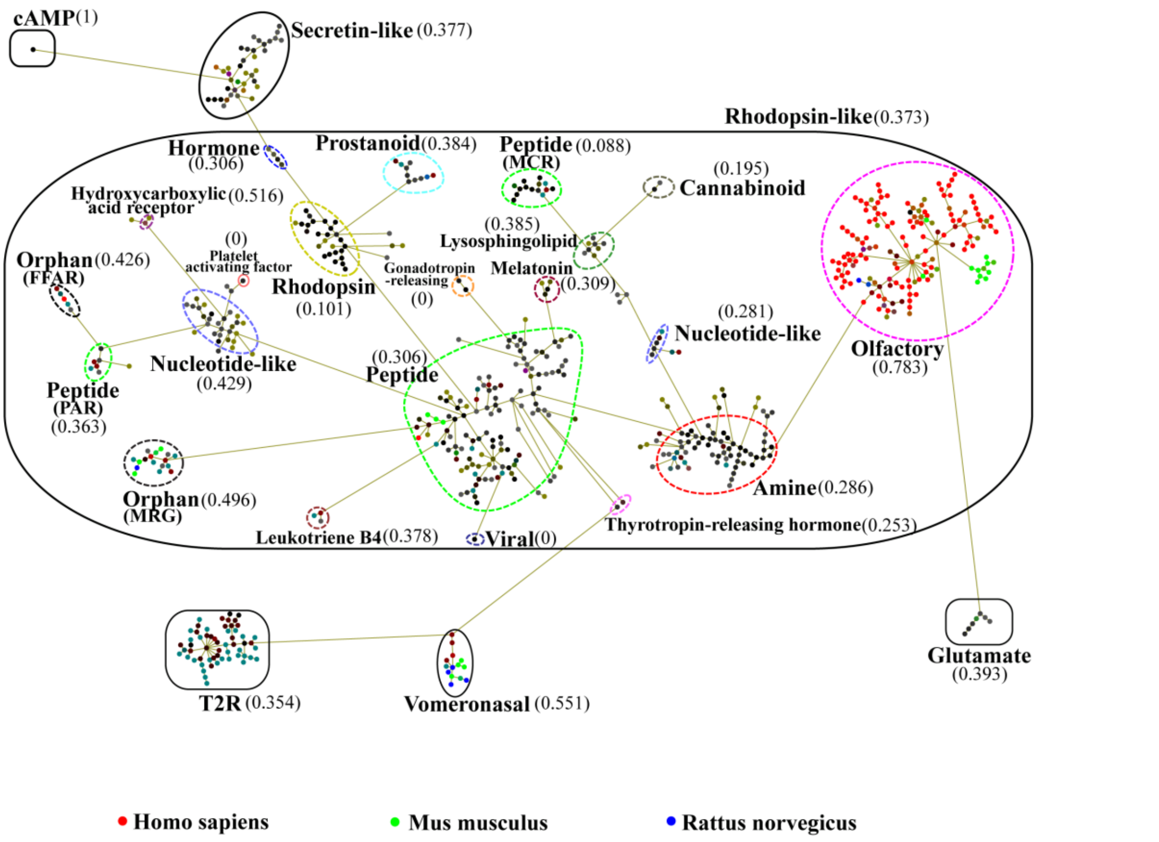

Figure S2. The minimum spanning tree diagram of the 620 level 1 MSC clusters for the GPCR network in the target dataset. Here each circle represents an MSC cluster the color of which is according to the types of organisms from which its member sequences are derived. Explicitly, to specify the species origin of sequences in each MSC cluster, we calculate the percentage of Homo sapiens (*h*), Mus musculus (*m*), Rattus norvegicus (*r*), and others ($1-h-m-r$). The (R, G, B) color of each node is calculated as ($255\times h$, $255\times m$, $255\times r$). Numbers in the parentheses are the average percentage of paralog pairs for various functional clusters (averaged over all nodes in each functional cluster).

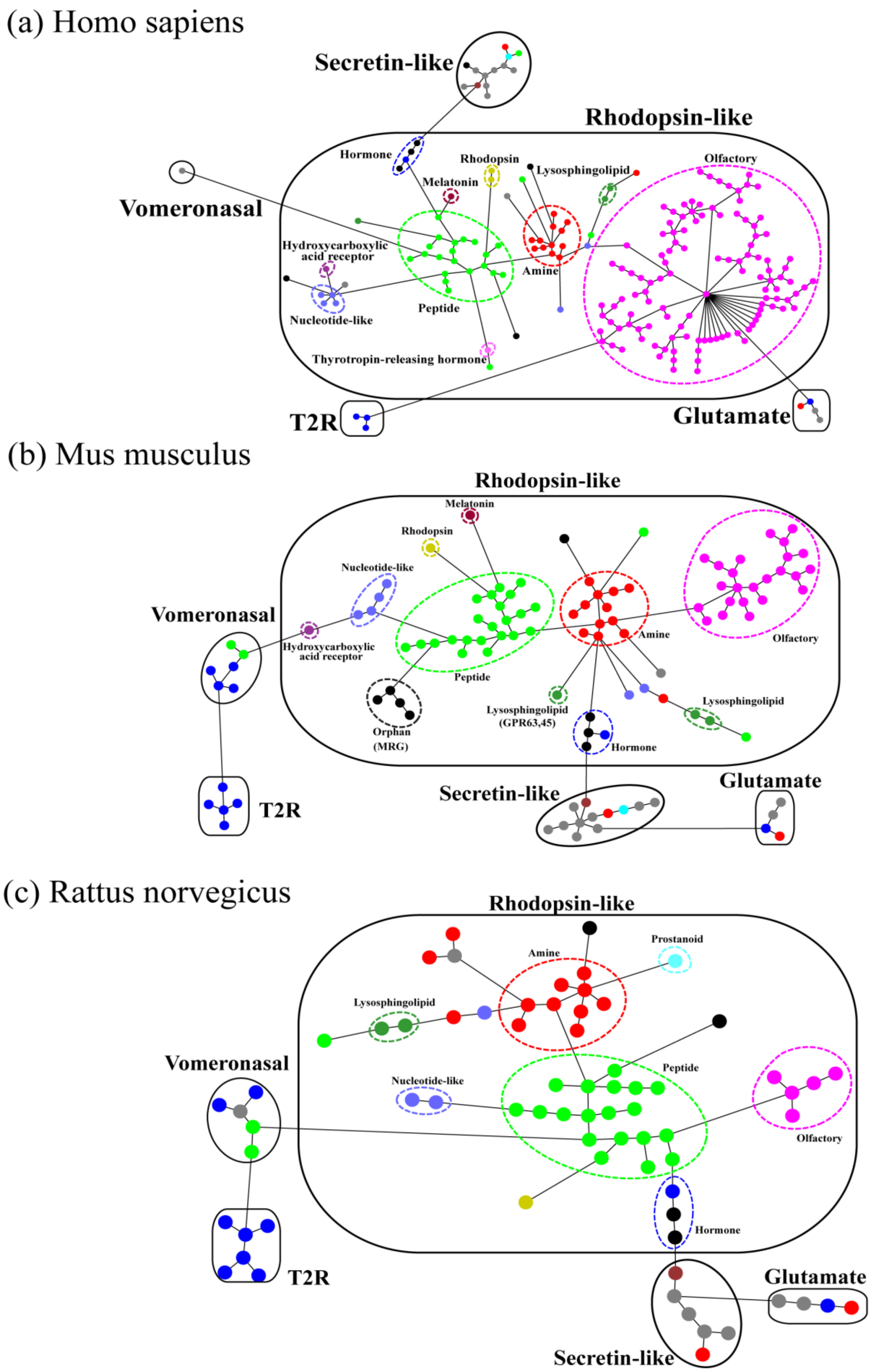

Figure S3. The minimum spanning tree diagrams of the GPCR network of Homo sapiens (a), Mus musculus (b), and Rattus norvegicus (c). Here each circle represents an MSC cluster the color of which is according to the color scheme in Fig. 5.

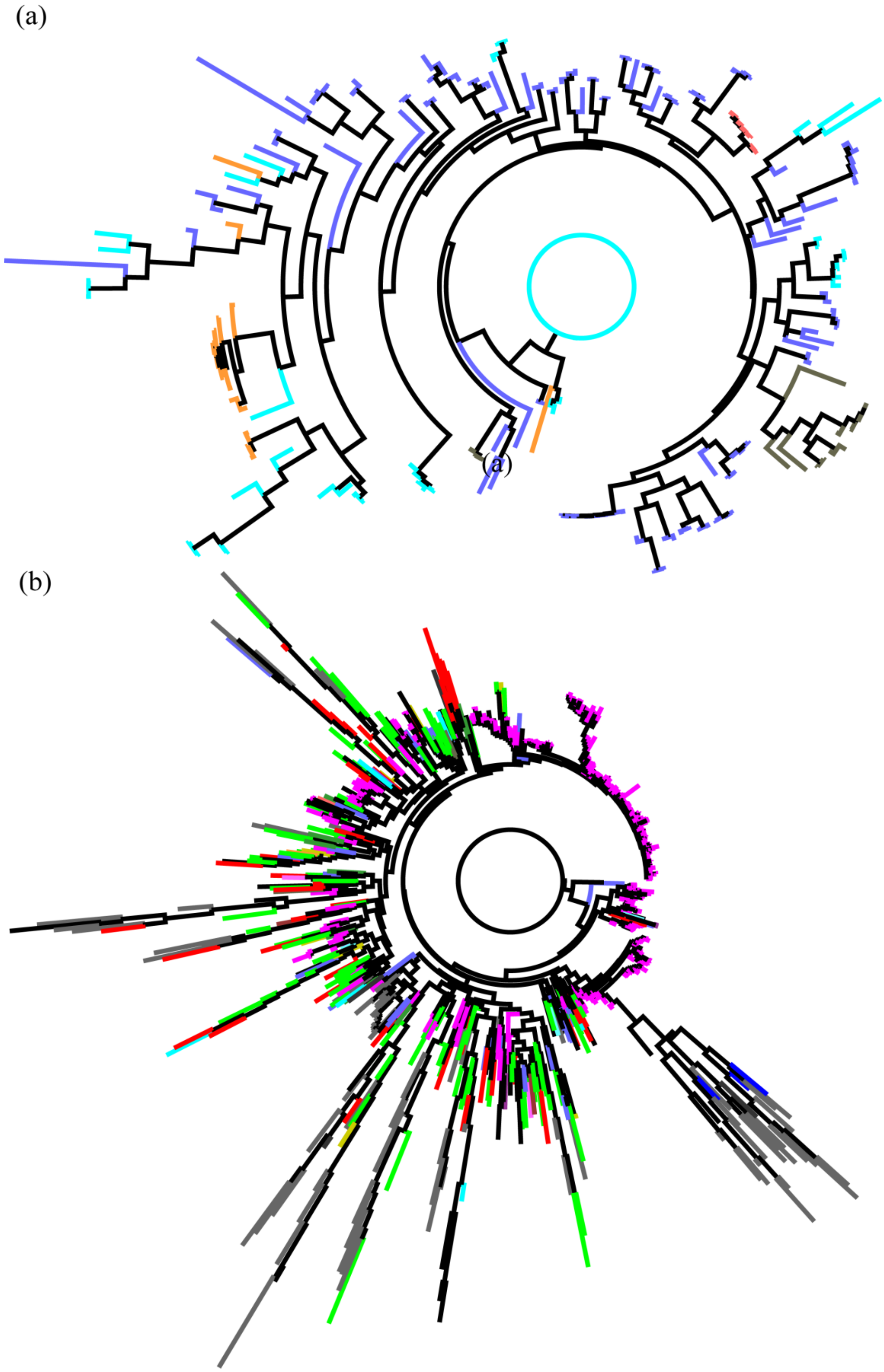

Figure S4. Phylogenetic tree of 185 GPCR sequences in the target dataset (a) and that of 804 GPCR sequences in the Homo sapiens dataset (b) constructed by the NJ method. The color scheme is the same as that in Fig. 5.

Table S1. MSC clustering of 2770 GPCR sequences in the target dataset.

MSC cluster label	Uniprot ID	GPCRDB label (level 2)	GPCRDB function label
Am-001	Q90WY4	Amine	Alpha Adrenoceptors type 2a
Am-001	Q60474	Amine	Alpha Adrenoceptors type 2a
Am-001	P18871	Amine	Alpha Adrenoceptors type 2a
Am-001	Q01338	Amine	Alpha Adrenoceptors type 2a
Am-001	P22909	Amine	Alpha Adrenoceptors type 2a
Am-001	Q28838	Amine	Alpha Adrenoceptors type 2a
Am-001	P08913	Amine	Alpha Adrenoceptors type 2a
Am-001	Q90WY5	Amine	Alpha Adrenoceptors type 2b
Am-002	P79748	Amine	Serotonin type 1d
Am-002	P49145	Amine	Serotonin type 1d
Am-002	P28565	Amine	Serotonin type 1d
Am-002	P28221	Amine	Serotonin type 1d
Am-002	P79400	Amine	Serotonin type 1d
Am-002	Q61224	Amine	Serotonin type 1d
Am-002	P11614	Amine	Serotonin type 1d
Am-002	Q60484	Amine	Serotonin type 1d
Am-003	P21917	Amine	Dopamine Vertebrate type 4
Am-003	P30729	Amine	Dopamine Vertebrate type 4
Am-003	Q6TLJ0	Amine	Dopamine Vertebrate type 4
Am-003	P51436	Amine	Dopamine Vertebrate type 4
Am-004	P22270	Amine	Octopamine type 4
Am-004	Q17232	Amine	Octopamine type 6
Am-004	Q25188	Amine	Octopamine type 6
Am-004	O02213	Amine	Octopamine type 6
Am-005	Q4LBB6	Amine	Dopamine Non Vertebrate
Am-005	Q9VCZ3	Amine	Octopamine type 1
Am-005	Q4LBB9	Amine	Octopamine type 2
Am-006	Q25414	Amine	Serotonin Insect
Am-006	Q25190	Amine	Serotonin Insect
Am-006	Q17239	Amine	Serotonin Insect
Am-007	P30545	Amine	Alpha Adrenoceptors type 2b
Am-007	Q60475	Amine	Alpha Adrenoceptors type 2b
Am-007	P18089	Amine	Alpha Adrenoceptors type 2b
Am-007	O19012	Amine	Alpha Adrenoceptors type 2b
Am-007	O77830	Amine	Alpha Adrenoceptors type 2b
Am-007	O77715	Amine	Alpha Adrenoceptors type 2b
Am-007	O18935	Amine	Alpha Adrenoceptors type 2b
Am-007	P19328	Amine	Alpha Adrenoceptors type 2b
Am-007	O77721	Amine	Alpha Adrenoceptors type 2b
Am-007	O19014	Amine	Alpha Adrenoceptors type 2b
Am-007	O19054	Amine	Alpha Adrenoceptors type 2b

Am-007	O19091	Amine	Alpha Adrenoceptors type 2b
Am-007	O77700	Amine	Alpha Adrenoceptors type 2b
Am-007	O77713	Amine	Alpha Adrenoceptors type 2b
Am-008	Q6XXY0	Amine	Serotonin type 1a
Am-008	Q9N298	Amine	Serotonin type 1a
Am-008	Q98998	Amine	Serotonin type 1a
Am-008	P19327	Amine	Serotonin type 1a
Am-008	Q9N297	Amine	Serotonin type 1a
Am-008	Q64264	Amine	Serotonin type 1a
Am-008	O42384	Amine	Serotonin type 1a
Am-008	O42385	Amine	Serotonin type 1a
Am-008	Q9N296	Amine	Serotonin type 1a
Am-008	Q0EAB6	Amine	Serotonin type 1a
Am-008	Q6XXX9	Amine	Serotonin type 1a
Am-008	P08908	Amine	Serotonin type 1a
Am-009	Q4KWL2	Amine	Beta Adrenoceptors type 2
Am-009	P54833	Amine	Beta Adrenoceptors type 2
Am-009	Q28509	Amine	Beta Adrenoceptors type 2
Am-009	P07550	Amine	Beta Adrenoceptors type 2
Am-009	Q8K4Z4	Amine	Beta Adrenoceptors type 2
Am-009	Q28997	Amine	Beta Adrenoceptors type 2
Am-009	P10608	Amine	Beta Adrenoceptors type 2
Am-009	Q8UUY8	Amine	Beta Adrenoceptors type 2
Am-009	Q9TST5	Amine	Beta Adrenoceptors type 2
Am-009	P18762	Amine	Beta Adrenoceptors type 2
Am-009	Q28044	Amine	Beta Adrenoceptors type 2
Am-009	P04274	Amine	Beta Adrenoceptors type 2
Am-010	Q9TST4	Amine	Beta Adrenoceptors type 3
Am-010	Q28524	Amine	Beta Adrenoceptors type 3
Am-010	P46626	Amine	Beta Adrenoceptors type 3
Am-010	Q60483	Amine	Beta Adrenoceptors type 3
Am-010	P13945	Amine	Beta Adrenoceptors type 3
Am-010	Q95252	Amine	Beta Adrenoceptors type 3
Am-010	O02662	Amine	Beta Adrenoceptors type 3
Am-010	Q9XT58	Amine	Beta Adrenoceptors type 3
Am-010	Q9XT57	Amine	Beta Adrenoceptors type 3
Am-010	P25962	Amine	Beta Adrenoceptors type 3
Am-010	P26255	Amine	Beta Adrenoceptors type 3
Am-011	Q9U7D5	Amine	Musc. acetylcholine Non Vertebrate
Am-011	Q9N2A3	Amine	Musc. acetylcholine Vertebrate type 3
Am-011	P20309	Amine	Musc. acetylcholine Vertebrate type 3
Am-011	P49578	Amine	Musc. acetylcholine Vertebrate type 3
Am-011	P08483	Amine	Musc. acetylcholine Vertebrate type 3
Am-011	P11483	Amine	Musc. acetylcholine Vertebrate type 3
Am-011	Q9N2A2	Amine	Musc. acetylcholine Vertebrate type 3

Am-011	P41984	Amine	Musc. acetylcholine Vertebrate type 3
Am-011	Q9ERZ3	Amine	Musc. acetylcholine Vertebrate type 3
Am-012	Q28998	Amine	Beta Adrenoceptors type 1
Am-012	Q9TST6	Amine	Beta Adrenoceptors type 1
Am-012	P08588	Amine	Beta Adrenoceptors type 1
Am-012	P79148	Amine	Beta Adrenoceptors type 1
Am-012	P47899	Amine	Beta Adrenoceptors type 1
Am-012	P18090	Amine	Beta Adrenoceptors type 1
Am-012	P34971	Amine	Beta Adrenoceptors type 1
Am-012	Q9TT96	Amine	Beta Adrenoceptors type 1
Am-012	P43141	Amine	Beta Adrenoceptors type 4
Am-013	P43140	Amine	Alpha Adrenoceptors type 1a
Am-013	Q9WU25	Amine	Alpha Adrenoceptors type 1a
Am-013	Q91175	Amine	Alpha Adrenoceptors type 1a
Am-013	P97718	Amine	Alpha Adrenoceptors type 1a
Am-013	O02824	Amine	Alpha Adrenoceptors type 1a
Am-013	P18130	Amine	Alpha Adrenoceptors type 1a
Am-013	P35348	Amine	Alpha Adrenoceptors type 1a
Am-014	P32305	Amine	Serotonin type 7
Am-014	P50407	Amine	Serotonin type 7
Am-014	P34969	Amine	Serotonin type 7
Am-014	P32304	Amine	Serotonin type 7
Am-014	Q91559	Amine	Serotonin type 7
Am-015	P35405	Amine	Alpha Adrenoceptors type 2c
Am-015	Q01337	Amine	Alpha Adrenoceptors type 2c
Am-015	P18825	Amine	Alpha Adrenoceptors type 2c
Am-015	Q60476	Amine	Alpha Adrenoceptors type 2c
Am-015	P22086	Amine	Alpha Adrenoceptors type 2c
Am-016	Q9QXI3	Amine	Trace amine type 9
Am-016	Q8BZA7	Amine	Trace amine type 9
Am-016	Q96P69	Amine	Trace amine type 9
Am-016	Q8NDV2	Amine	Trace amine type 9
Am-017	Q02152	Amine	Serotonin type 2b
Am-017	Q8UUG8	Amine	Serotonin type 2b
Am-017	P30994	Amine	Serotonin type 2b
Am-017	P41595	Amine	Serotonin type 2b
Am-018	Q8TDV5	Amine	Trace amine type 9
Am-018	Q7TQP3	Amine	Trace amine type 9
Am-018	Q7TQN8	Amine	Trace amine type 9
Am-019	Q91ZY2	Amine	Histamine type 4
Am-019	Q91ZY1	Amine	Histamine type 4
Am-019	Q9H3N8	Amine	Histamine type 4
Am-020	Q91081	Amine	Alpha Adrenoceptors type 2c
Am-020	Q90WY6	Amine	Alpha Adrenoceptors type 2c
Am-021	P28285	Amine	Serotonin Insect

Am-021	P28286	Amine	Serotonin Insect
Am-022	O42574	Amine	Beta Adrenoceptors type 1
Am-022	P07700	Amine	Beta Adrenoceptors type 1
Am-023	P35404	Amine	Serotonin type 1b
Am-023	P28222	Amine	Serotonin type 1b
Am-023	O08892	Amine	Serotonin type 1b
Am-023	P60020	Amine	Serotonin type 1b
Am-023	Q0EAB5	Amine	Serotonin type 1b
Am-023	P46636	Amine	Serotonin type 1b
Am-023	Q588Y6	Amine	Serotonin type 1b
Am-023	P49144	Amine	Serotonin type 1b
Am-023	P56496	Amine	Serotonin type 1b
Am-023	P28334	Amine	Serotonin type 1b
Am-023	P79250	Amine	Serotonin type 1b
Am-023	P28564	Amine	Serotonin type 1b
Am-023	Q6XXX8	Amine	Serotonin type 1b
Am-024	Q923Y2	Amine	Trace amine type 15
Am-024	Q5QD12	Amine	Trace amine type 15
Am-024	Q5QD11	Amine	Trace amine type 7
Am-024	Q5QD09	Amine	Trace amine type 7
Am-024	Q923X5	Amine	Trace amine type 7
Am-024	Q923X6	Amine	Trace amine type 7
Am-024	Q923Y4	Amine	Trace amine type 7
Am-024	Q923Y1	Amine	Trace amine type 7
Am-024	Q5QD21	Amine	Trace amine type 7
Am-024	Q5QD10	Amine	Trace amine type 7
Am-024	Q5QD08	Amine	Trace amine type 7
Am-024	Q923X8	Amine	Trace amine type 7
Am-025	Q6TLI9	Amine	Dopamine Vertebrate type 2
Am-025	Q9GJU1	Amine	Dopamine Vertebrate type 2
Am-025	P61168	Amine	Dopamine Vertebrate type 2
Am-025	P34973	Amine	Dopamine Vertebrate type 2
Am-025	O73810	Amine	Dopamine Vertebrate type 2
Am-025	P60026	Amine	Dopamine Vertebrate type 2
Am-025	P52702	Amine	Dopamine Vertebrate type 2
Am-025	P24628	Amine	Dopamine Vertebrate type 2
Am-025	P61169	Amine	Dopamine Vertebrate type 2
Am-025	P20288	Amine	Dopamine Vertebrate type 2
Am-025	P53453	Amine	Dopamine Vertebrate type 2
Am-025	P14416	Amine	Dopamine Vertebrate type 2
Am-026	Q969N4	Amine	Trace amine type 17
Am-026	Q923Y5	Amine	Trace amine type 6
Am-026	Q5QD13	Amine	Trace amine type 6
Am-026	Q5W8W0	Amine	Trace amine type 6
Am-026	Q96RI8	Amine	Trace amine type 6

Am-026	Q923X9	Amine	Trace amine type 8
Am-026	Q923Y3	Amine	Trace amine type 8
Am-026	Q5QD06	Amine	Trace amine type 8
Am-026	Q923Y0	Amine	Trace amine type 8
Am-026	Q5QD05	Amine	Trace amine type 8
Am-026	Q5QD07	Amine	Trace amine type 8
Am-027	Q9ERZ4	Amine	Musc. acetylcholine Vertebrate type 2
Am-027	P10980	Amine	Musc. acetylcholine Vertebrate type 2
Am-027	P08172	Amine	Musc. acetylcholine Vertebrate type 2
Am-027	Q9N2A7	Amine	Musc. acetylcholine Vertebrate type 2
Am-027	P41985	Amine	Musc. acetylcholine Vertebrate type 2
Am-027	P06199	Amine	Musc. acetylcholine Vertebrate type 2
Am-027	P30372	Amine	Musc. acetylcholine Vertebrate type 2
Am-027	P08173	Amine	Musc. acetylcholine Vertebrate type 4
Am-027	P32211	Amine	Musc. acetylcholine Vertebrate type 4
Am-027	P17200	Amine	Musc. acetylcholine Vertebrate type 4
Am-028	P42289	Amine	Dopamine Vertebrate type 1
Am-028	Q95136	Amine	Dopamine Vertebrate type 1
Am-028	P21728	Amine	Dopamine Vertebrate type 1
Am-028	Q61616	Amine	Dopamine Vertebrate type 1
Am-028	P50130	Amine	Dopamine Vertebrate type 1
Am-028	P35406	Amine	Dopamine Vertebrate type 1
Am-028	P42288	Amine	Dopamine Vertebrate type 1
Am-028	O77680	Amine	Dopamine Vertebrate type 1
Am-028	P53452	Amine	Dopamine Vertebrate type 1
Am-028	P18901	Amine	Dopamine Vertebrate type 1
Am-029	P14842	Amine	Serotonin type 2a
Am-029	Q75Z89	Amine	Serotonin type 2a
Am-029	P18599	Amine	Serotonin type 2a
Am-029	Q5R4Q6	Amine	Serotonin type 2a
Am-029	P28223	Amine	Serotonin type 2a
Am-029	P35363	Amine	Serotonin type 2a
Am-029	P50129	Amine	Serotonin type 2a
Am-029	P50128	Amine	Serotonin type 2a
Am-029	O46635	Amine	Serotonin type 2a
Am-030	Q76MS7	Amine	Histamine type 2
Am-030	P25021	Amine	Histamine type 2
Am-030	P61752	Amine	Histamine type 2
Am-030	P17124	Amine	Histamine type 2
Am-030	P60021	Amine	Histamine type 2
Am-030	P25102	Amine	Histamine type 2
Am-030	P97292	Amine	Histamine type 2
Am-030	P47747	Amine	Histamine type 2
Am-031	P31389	Amine	Histamine type 1
Am-031	Q9N2B0	Amine	Histamine type 1

Am-031	P31390	Amine	Histamine type 1
Am-031	P70174	Amine	Histamine type 1
Am-031	Q9N2B1	Amine	Histamine type 1
Am-031	P30546	Amine	Histamine type 1
Am-031	P35367	Amine	Histamine type 1
Am-032	Q5IS53	Amine	Musc. acetylcholine Vertebrate type 5
Am-032	Q5IS98	Amine	Musc. acetylcholine Vertebrate type 5
Am-032	Q920H4	Amine	Musc. acetylcholine Vertebrate type 5
Am-032	P56490	Amine	Musc. acetylcholine Vertebrate type 5
Am-032	P08911	Amine	Musc. acetylcholine Vertebrate type 5
Am-032	P08912	Amine	Musc. acetylcholine Vertebrate type 5
Am-033	P12657	Amine	Musc. acetylcholine Vertebrate type 1
Am-033	P11229	Amine	Musc. acetylcholine Vertebrate type 1
Am-033	Q5R949	Amine	Musc. acetylcholine Vertebrate type 1
Am-033	P08482	Amine	Musc. acetylcholine Vertebrate type 1
Am-033	P56489	Amine	Musc. acetylcholine Vertebrate type 1
Am-033	P04761	Amine	Musc. acetylcholine Vertebrate type 1
Am-034	P28335	Amine	Serotonin type 1c
Am-034	P34968	Amine	Serotonin type 1c
Am-034	Q60F97	Amine	Serotonin type 1c
Am-034	P08909	Amine	Serotonin type 1c
Am-034	Q5IS66	Amine	Serotonin type 1c
Am-035	P97717	Amine	Alpha Adrenoceptors type 1b
Am-035	P18841	Amine	Alpha Adrenoceptors type 1b
Am-035	P15823	Amine	Alpha Adrenoceptors type 1b
Am-035	P35368	Amine	Alpha Adrenoceptors type 1b
Am-035	P11615	Amine	Alpha Adrenoceptors type 1b
Am-036	P52703	Amine	Dopamine Vertebrate type 3
Am-036	Q5IS72	Amine	Dopamine Vertebrate type 3
Am-036	P35462	Amine	Dopamine Vertebrate type 3
Am-036	P30728	Amine	Dopamine Vertebrate type 3
Am-036	P19020	Amine	Dopamine Vertebrate type 3
Am-037	Q9TTM9	Amine	Alpha Adrenoceptors type 1d
Am-037	P25100	Amine	Alpha Adrenoceptors type 1d
Am-037	P23944	Amine	Alpha Adrenoceptors type 1d
Am-037	P97714	Amine	Alpha Adrenoceptors type 1d
Am-037	O02666	Amine	Alpha Adrenoceptors type 1d
Am-038	Q9QYN8	Amine	Histamine type 3
Am-038	P58406	Amine	Histamine type 3
Am-038	Q9JI35	Amine	Histamine type 3
Am-038	Q9Y5N1	Amine	Histamine type 3
Am-039	Q13639	Amine	Serotonin type 4
Am-039	P97288	Amine	Serotonin type 4
Am-039	Q62758	Amine	Serotonin type 4
Am-039	O70528	Amine	Serotonin type 4

Am-040	P25115	Amine	Dopamine Vertebrate type 5
Am-040	Q8BLD9	Amine	Dopamine Vertebrate type 5
Am-040	P42290	Amine	Dopamine Vertebrate type 5
Am-040	P21918	Amine	Dopamine Vertebrate type 5
Am-041	P30940	Amine	Serotonin type 1f
Am-041	P30939	Amine	Serotonin type 1f
Am-041	Q02284	Amine	Serotonin type 1f
Am-041	O08890	Amine	Serotonin type 1f
Am-042	O14804	Amine	Trace amine type 5
Am-042	Q5QD23	Amine	Trace amine type 5
Am-042	Q5QD28	Amine	Trace amine type 5
Am-042	Q5QD14	Amine	Trace amine type 5
Am-043	P50406	Amine	Serotonin type 6
Am-043	P31388	Amine	Serotonin type 6
Am-043	Q5IS65	Amine	Serotonin type 6
Am-043	Q9R1C8	Amine	Serotonin type 6
Am-044	P47800	Amine	Dopamine Vertebrate type 1
Am-044	P53454	Amine	Dopamine Vertebrate type 1
Am-044	P42291	Amine	Dopamine Vertebrate type 1
Am-045	P35364	Amine	Serotonin type 5a
Am-045	P47898	Amine	Serotonin type 5a
Am-045	P30966	Amine	Serotonin type 5a
Am-046	P30951	Amine	Trace amine type 9
Am-046	P35412	Amine	Trace amine type 9
Am-046	P47775	Amine	Trace amine type 9
Am-047	P32251	Amine	Alpha Adrenoceptors type 2d
Am-047	Q8JG70	Amine	Alpha Adrenoceptors type 2d
Am-047	Q8JG69	Amine	Alpha Adrenoceptors type 2d
Am-048	Q6VB83	Amine	Serotonin type 1e
Am-048	P28566	Amine	Serotonin type 1e
Am-048	Q9N2B6	Amine	Serotonin type 1e
Am-049	Q8K1Q3	Amine	Trace amine type 9
Am-049	P46089	Amine	Trace amine type 9
Am-049	P35413	Amine	Trace amine type 9
Am-050	Q5QD16	Amine	Trace amine type 3
Am-050	Q5QD24	Amine	Trace amine type 3
Am-050	Q9P1P4	Amine	Trace amine type 3
Am-051	Q9P1P5	Amine	Trace amine type 2
Am-051	Q5QD17	Amine	Trace amine type 2
Am-051	Q5QD25	Amine	Trace amine type 2
Am-052	Q8HZ64	Amine	Trace amine type 1
Am-052	Q5QD29	Amine	Trace amine type 1
Am-052	Q96RJ0	Amine	Trace amine type 1
Am-053	Q8IZ08	Amine	Dopamine Vertebrate type 1
Am-053	Q7TQP2	Amine	Dopamine Vertebrate type 1

Am-053	Q7TQN7	Amine	Dopamine Vertebrate type 1
Am-054	Q6YNI2	Amine	Trace amine type 9
Am-054	P51651	Amine	Trace amine type 9
Am-054	P46095	Amine	Trace amine type 9
Am-055	Q25322	Amine	Octopamine type 6
Am-055	Q25321	Amine	Octopamine type 6
Am-056	O95800	Amine	Trace amine type 9
Am-056	Q6X632	Amine	Trace amine type 9
Am-057	Q16950	Amine	Dopamine Insect type 1
Am-057	Q16951	Amine	Dopamine Insect type 1
Am-058	Q923Y9	Amine	Trace amine type 1
Am-058	Q923Y8	Amine	Trace amine type 1
Am-059	P31387	Amine	Serotonin type 5b
Am-059	P35365	Amine	Serotonin type 5b
Am-060	Q61H86	Amine	Dopamine Other
Am-060	Q6RYS9	Amine	Dopamine Other
Ca-001	Q98895	Cannabinoid	Cannabinoid type 1
Ca-001	Q71SP5	Cannabinoid	Cannabinoid type 1
Ca-001	P21554	Cannabinoid	Cannabinoid type 1
Ca-001	O02777	Cannabinoid	Cannabinoid type 1
Ca-001	P56971	Cannabinoid	Cannabinoid type 1
Ca-001	Q333S9	Cannabinoid	Cannabinoid type 1
Ca-001	Q801M1	Cannabinoid	Cannabinoid type 1
Ca-001	P47746	Cannabinoid	Cannabinoid type 1
Ca-001	Q5IS73	Cannabinoid	Cannabinoid type 1
Ca-001	Q9PUI7	Cannabinoid	Cannabinoid type 1
Ca-001	Q98894	Cannabinoid	Cannabinoid type 1
Ca-001	P20272	Cannabinoid	Cannabinoid type 1
Ca-002	Q9QZN9	Cannabinoid	Cannabinoid type 2
Ca-002	P47936	Cannabinoid	Cannabinoid type 2
Ca-002	P34972	Cannabinoid	Cannabinoid type 2
Go-001	Q01776	Gonadotropin-releasing hormone	Gonadotropin-releasing hormone type I
Go-001	Q9MZI6	Gonadotropin-releasing hormone	Gonadotropin-releasing hormone type I
Go-001	P30968	Gonadotropin-releasing hormone	Gonadotropin-releasing hormone type I
Go-001	P32236	Gonadotropin-releasing hormone	Gonadotropin-releasing hormone type I
Go-001	O18821	Gonadotropin-releasing hormone	Gonadotropin-releasing hormone type I
Go-001	P32237	Gonadotropin-releasing hormone	Gonadotropin-releasing hormone type I
Go-001	P49922	Gonadotropin-releasing hormone	Gonadotropin-releasing hormone type I
Go-001	Q9TTI8	Gonadotropin-releasing hormone	Gonadotropin-releasing hormone type I
Go-001	Q8CH60	Gonadotropin-releasing hormone	Gonadotropin-releasing hormone type I
Go-001	Q19PY9	Gonadotropin-releasing hormone	Gonadotropin-releasing hormone type I
Go-001	P30969	Gonadotropin-releasing hormone	Gonadotropin-releasing hormone type I
Go-002	Q95MG6	Gonadotropin-releasing hormone	Gonadotropin-releasing hormone type II
Go-002	Q95MH6	Gonadotropin-releasing hormone	Gonadotropin-releasing hormone type II
Go-002	Q95JG1	Gonadotropin-releasing hormone	Gonadotropin-releasing hormone type II

Go-002	O42329	Gonadotropin-releasing hormone	Gonadotropin-releasing hormone type II
Ho-001	Q95179	Hormone protein	Follicle stimulating hormone
Ho-001	P23945	Hormone protein	Follicle stimulating hormone
Ho-001	P79763	Hormone protein	Follicle stimulating hormone
Ho-001	Q6YNB6	Hormone protein	Follicle stimulating hormone
Ho-001	P35378	Hormone protein	Follicle stimulating hormone
Ho-001	P47799	Hormone protein	Follicle stimulating hormone
Ho-001	Q6R6L8	Hormone protein	Follicle stimulating hormone
Ho-001	P32212	Hormone protein	Follicle stimulating hormone
Ho-001	P35379	Hormone protein	Follicle stimulating hormone
Ho-001	Q8R428	Hormone protein	Follicle stimulating hormone
Ho-001	P35376	Hormone protein	Follicle stimulating hormone
Ho-001	Q5GJ04	Hormone protein	Follicle stimulating hormone
Ho-001	Q7ZTV5	Hormone protein	Follicle stimulating hormone
Ho-001	P49059	Hormone protein	Follicle stimulating hormone
Ho-001	P20395	Hormone protein	Follicle stimulating hormone
Ho-001	O02721	Hormone protein	Lutropin-choriogonadotropic hormone
Ho-001	P16582	Hormone protein	Lutropin-choriogonadotropic hormone
Ho-001	Q28005	Hormone protein	Lutropin-choriogonadotropic hormone
Ho-001	P16235	Hormone protein	Lutropin-choriogonadotropic hormone
Ho-001	Q28585	Hormone protein	Lutropin-choriogonadotropic hormone
Ho-001	P30730	Hormone protein	Lutropin-choriogonadotropic hormone
Ho-001	P22888	Hormone protein	Lutropin-choriogonadotropic hormone
Ho-001	P56495	Hormone protein	Thyrotropin
Ho-001	Q8SPP9	Hormone protein	Thyrotropin
Ho-001	Q6QMG1	Hormone protein	Thyrotropin
Ho-001	P35409	Hormone protein	Thyrotropin
Ho-001	P47750	Hormone protein	Thyrotropin
Ho-001	P14763	Hormone protein	Thyrotropin
Ho-001	Q27987	Hormone protein	Thyrotropin
Ho-001	P21463	Hormone protein	Thyrotropin
Ho-001	P16473	Hormone protein	Thyrotropin
Ho-001	Q9BGN4	Hormone protein	Thyrotropin
Hy-001	P49019	Hydroxycarboxylic acid receptor	GPR109
Hy-001	Q8TDS4	Hydroxycarboxylic acid receptor	GPR109
Hy-001	Q9EP66	Hydroxycarboxylic acid receptor	GPR109
Hy-001	Q80Z39	Hydroxycarboxylic acid receptor	GPR109
Hy-002	Q9BXC0	Hydroxycarboxylic acid receptor	GPR81
Hy-002	Q8C131	Hydroxycarboxylic acid receptor	GPR81
Le-001	Q9JJL9	Leukotriene B4 receptor	Leukotriene B4 receptor type 2
Le-001	Q924U0	Leukotriene B4 receptor	Leukotriene B4 receptor type 2
Le-001	Q9NPC1	Leukotriene B4 receptor	Leukotriene B4 receptor type 2
Le-002	Q9R0Q2	Leukotriene B4 receptor	Leukotriene B4 receptor type 1
Le-002	O88855	Leukotriene B4 receptor	Leukotriene B4 receptor type 1
Le-003	Q3T181	Leukotriene B4 receptor	Leukotriene B4 receptor type 1

Le-003	Q15722	Leukotriene B4 receptor	Leukotriene B4 receptor type 1
Ly-001	Q9DDK4	Lysosphingolipid and LPA (EDG)	Sphingosine 1-phosphate Edg-1
Ly-001	P21453	Lysosphingolipid and LPA (EDG)	Sphingosine 1-phosphate Edg-1
Ly-001	O08530	Lysosphingolipid and LPA (EDG)	Sphingosine 1-phosphate Edg-1
Ly-001	Q5E9P3	Lysosphingolipid and LPA (EDG)	Sphingosine 1-phosphate Edg-1
Ly-001	P48303	Lysosphingolipid and LPA (EDG)	Sphingosine 1-phosphate Edg-1
Ly-002	Q9I8K8	Lysosphingolipid and LPA (EDG)	Sphingosine 1-phosphate Edg-5
Ly-002	P47752	Lysosphingolipid and LPA (EDG)	Sphingosine 1-phosphate Edg-5
Ly-002	P52592	Lysosphingolipid and LPA (EDG)	Sphingosine 1-phosphate Edg-5
Ly-002	O95136	Lysosphingolipid and LPA (EDG)	Sphingosine 1-phosphate Edg-5
Ly-003	Q9HBW0	Lysosphingolipid and LPA (EDG)	Lysophosphatidic acid Edg-4
Ly-003	Q9JL06	Lysosphingolipid and LPA (EDG)	Lysophosphatidic acid Edg-4
Ly-003	Q95KH4	Lysosphingolipid and LPA (EDG)	Lysophosphatidic acid Edg-4
Ly-004	Q9EQQ4	Lysosphingolipid and LPA (EDG)	GPR45
Ly-004	Q9Y5Y3	Lysosphingolipid and LPA (EDG)	GPR45
Ly-004	P79945	Lysosphingolipid and LPA (EDG)	GPR45
Ly-005	Q99500	Lysosphingolipid and LPA (EDG)	Sphingosine 1-phosphate Edg-3
Ly-005	Q9Z0U9	Lysosphingolipid and LPA (EDG)	Sphingosine 1-phosphate Edg-3
Ly-005	Q9PUQ8	Lysosphingolipid and LPA (EDG)	Sphingosine 1-phosphate Edg-3
Ly-006	Q9PU17	Lysosphingolipid and LPA (EDG)	Lysophosphatidic acid Edg-2
Ly-006	P61793	Lysosphingolipid and LPA (EDG)	Lysophosphatidic acid Edg-2
Ly-006	P61794	Lysosphingolipid and LPA (EDG)	Lysophosphatidic acid Edg-2
Ly-006	Q92633	Lysosphingolipid and LPA (EDG)	Lysophosphatidic acid Edg-2
Ly-006	Q9PU16	Lysosphingolipid and LPA (EDG)	Lysophosphatidic acid Edg-2
Ly-006	Q28031	Lysosphingolipid and LPA (EDG)	Lysophosphatidic acid Edg-2
Ly-006	P46628	Lysosphingolipid and LPA (EDG)	Lysophosphatidic acid Edg-2
Ly-007	Q9H228	Lysosphingolipid and LPA (EDG)	Sphingosine 1-phosphate Edg-8
Ly-007	Q9JKM5	Lysosphingolipid and LPA (EDG)	Sphingosine 1-phosphate Edg-8
Ly-007	Q684M3	Lysosphingolipid and LPA (EDG)	Sphingosine 1-phosphate Edg-8
Ly-007	Q91X56	Lysosphingolipid and LPA (EDG)	Sphingosine 1-phosphate Edg-8
Ly-008	Q9EQ31	Lysosphingolipid and LPA (EDG)	Lysophosphatidic acid Edg-7
Ly-008	Q9UBY5	Lysosphingolipid and LPA (EDG)	Lysophosphatidic acid Edg-7
Ly-008	Q8K5E0	Lysosphingolipid and LPA (EDG)	Lysophosphatidic acid Edg-7
Ly-009	Q9BZJ6	Lysosphingolipid and LPA (EDG)	GPR63
Ly-009	Q9EQQ3	Lysosphingolipid and LPA (EDG)	GPR63
Ly-010	O95977	Lysosphingolipid and LPA (EDG)	Sphingosine 1-phosphate Edg-6
Ly-010	Q9Z0L1	Lysosphingolipid and LPA (EDG)	Sphingosine 1-phosphate Edg-6
Me-001	P48039	Melatonin	Melatonin type 1
Me-001	P49217	Melatonin	Melatonin type 1
Me-001	P51049	Melatonin	Melatonin type 1
Me-001	Q61184	Melatonin	Melatonin type 1
Me-001	Q90456	Melatonin	Melatonin type 1
Me-001	P51050	Melatonin	Melatonin type 1
Me-001	O02769	Melatonin	Melatonin type 1
Me-001	P49285	Melatonin	Melatonin type 1

Me-001	P48040	Melatonin	Melatonin type 1
Me-001	P51046	Melatonin	Melatonin type 1
Me-002	P49219	Melatonin	Melatonin type 1
Me-002	P49288	Melatonin	Melatonin type 1
Me-003	O88495	Melatonin	Melatonin-like (GPR50)
Me-003	Q28558	Melatonin	Melatonin-like (GPR50)
Me-003	Q13585	Melatonin	Melatonin-like (GPR50)
Me-004	P49286	Melatonin	Melatonin type 1
Me-004	Q8CIQ6	Melatonin	Melatonin type 1
Mix-001	Q6R6I7	Class A Orphan/other	LGR like (hormone receptors) type 7 and 8
Mix-001	Q5XM32	Class A Orphan/other	LGR like (hormone receptors) type 7 and 8
Mix-001	Q6R6I6	Class A Orphan/other	LGR like (hormone receptors) type 7 and 8
Mix-001	Q91ZZ5	Class A Orphan/other	LGR like (hormone receptors) type 7 and 8
Mix-001	Q9HBX9	Class A Orphan/other	LGR like (hormone receptors) type 7 and 8
Mix-001	Q5R5V8	Class A Orphan/other	LGR like (hormone receptors) type 7 and 8
Mix-001	Q8WXD0	Class A Orphan/other	LGR like (hormone receptors) type 7 and 8
Mix-001	P46023	Hormone protein	Gonadotropin type I
Mix-002	Q923Y7	Amine	Trace amine type 4
Mix-002	Q5QD15	Amine	Trace amine type 4
Mix-002	Q5QNP2	Class A Orphan/other	Other 509
Mix-003	Q14439	Class A Orphan/other	ORPH_MSC429
Mix-003	Q80WT4	Class A Orphan/other	ORPH_MSC429
Mix-003	Q64017	Class A Orphan/other	ORPH_MSC429
Mix-004	Q80T62	Gonadotropin-releasing hormone	Gonadotropin-releasing hormone type II
Mix-004	Q96P66	Peptide	C-C Chemokine type 5
Nu-001	Q0VC81	Nucleotide-like	Adenosine type 3
Nu-001	O02667	Nucleotide-like	Adenosine type 3
Nu-001	P33765	Nucleotide-like	Adenosine type 3
Nu-001	P35342	Nucleotide-like	Adenosine type 3
Nu-001	Q28309	Nucleotide-like	Adenosine type 3
Nu-002	Q9ESG6	Nucleotide-like	Purinoceptor P2RY12-14 GPR87 (UDP-Glucose)
Nu-002	O35881	Nucleotide-like	Purinoceptor P2RY12-14 GPR87 (UDP-Glucose)
Nu-002	Q3SX17	Nucleotide-like	Purinoceptor P2RY12-14 GPR87 (UDP-Glucose)
Nu-002	Q15391	Nucleotide-like	Purinoceptor P2RY12-14 GPR87 (UDP-Glucose)
Nu-003	Q149R9	Nucleotide-like	P2RY5
Nu-003	Q3ZC80	Nucleotide-like	P2RY5
Nu-003	Q9H1C0	Nucleotide-like	P2RY5
Nu-004	Q99MT6	Nucleotide-like	Succinate receptors
Nu-004	Q6IYF9	Nucleotide-like	Succinate receptors
Nu-004	Q9BXA5	Nucleotide-like	Succinate receptors
Nu-005	P28190	Nucleotide-like	Adenosine type 1
Nu-005	P11616	Nucleotide-like	Adenosine type 1
Nu-005	P49892	Nucleotide-like	Adenosine type 1
Nu-005	P34970	Nucleotide-like	Adenosine type 1
Nu-005	P47745	Nucleotide-like	Adenosine type 1

Nu-005	Q5RF57	Nucleotide-like	Adenosine type 1
Nu-005	Q60612	Nucleotide-like	Adenosine type 1
Nu-005	P30542	Nucleotide-like	Adenosine type 1
Nu-005	P25099	Nucleotide-like	Adenosine type 1
Nu-006	Q60614	Nucleotide-like	Adenosine type 2
Nu-006	P29276	Nucleotide-like	Adenosine type 2
Nu-006	P29275	Nucleotide-like	Adenosine type 2
Nu-006	Q32ZE2	Nucleotide-like	Adenosine type 2
Nu-006	Q1LZD0	Nucleotide-like	Adenosine type 2
Nu-006	O13076	Nucleotide-like	Adenosine type 2
Nu-006	Q6W3F4	Nucleotide-like	Adenosine type 2
Nu-007	Q9JJS7	Nucleotide-like	P2RY4
Nu-007	P51582	Nucleotide-like	P2RY4
Nu-007	O35811	Nucleotide-like	P2RY4
Nu-007	P79928	Nucleotide-like	P2RY4
Nu-008	Q8BMC0	Nucleotide-like	P2RY5
Nu-008	P43657	Nucleotide-like	P2RY5
Nu-008	Q4G072	Nucleotide-like	P2RY5
Nu-008	P32250	Nucleotide-like	P2RY9
Nu-009	Q9D8I2	Nucleotide-like	Purinoceptor P2RY12-14 GPR87 (UDP-Glucose)
Nu-009	Q6GUG4	Nucleotide-like	Purinoceptor P2RY12-14 GPR87 (UDP-Glucose)
Nu-009	Q9BPV8	Nucleotide-like	Purinoceptor P2RY12-14 GPR87 (UDP-Glucose)
Nu-010	Q9ERK9	Nucleotide-like	P2RY3and6
Nu-010	Q63371	Nucleotide-like	P2RY3and6
Nu-010	Q15077	Nucleotide-like	P2RY3and6
Nu-011	Q9UNW8	Nucleotide-like	P2RY5
Nu-011	Q9Z282	Nucleotide-like	P2RY5
Nu-012	Q61038	Nucleotide-like	P2RY5
Nu-012	Q8IYL9	Nucleotide-like	P2RY5
Nu-013	O00398	Nucleotide-like	P2RY5 other
Nu-013	Q8BFU7	Nucleotide-like	P2RY5 other
Nu-014	P49651	Nucleotide-like	P2RY1
Nu-014	P34996	Nucleotide-like	P2RY1
Nu-014	P49650	Nucleotide-like	P2RY1
Nu-014	P49652	Nucleotide-like	P2RY1
Nu-014	P47900	Nucleotide-like	P2RY1
Nu-014	P48042	Nucleotide-like	P2RY1
Nu-014	P59902	Nucleotide-like	P2RY1
Nu-015	P29274	Nucleotide-like	Adenosine type 2
Nu-015	P11617	Nucleotide-like	Adenosine type 2
Nu-015	P30543	Nucleotide-like	Adenosine type 2
Nu-015	Q60613	Nucleotide-like	Adenosine type 2
Nu-015	Q6TLI7	Nucleotide-like	Adenosine type 2
Nu-015	P46616	Nucleotide-like	Adenosine type 2
Nu-016	Q8BUD0	Nucleotide-like	P2RY5

Nu-016	Q4KLH9	Nucleotide-like	P2RY5
Nu-016	Q1JQB3	Nucleotide-like	P2RY5
Nu-016	P46093	Nucleotide-like	P2RY5
Nu-016	P50132	Nucleotide-like	P2RY5
Nu-017	B3DM66	Nucleotide-like	Adenosine type 1
Nu-017	B2RPY5	Nucleotide-like	Adenosine type 1
Nu-017	Q90X46	Nucleotide-like	Adenosine type 1
Nu-017	Q8N6U8	Nucleotide-like	Adenosine type 1
Nu-017	Q2YDN1	Nucleotide-like	Adenosine type 1
Nu-018	P41232	Nucleotide-like	P2RY2
Nu-018	Q5YA25	Nucleotide-like	P2RY2
Nu-018	P41231	Nucleotide-like	P2RY2
Nu-018	P35383	Nucleotide-like	P2RY2
Nu-019	Q9CPV9	Nucleotide-like	Purinoceptor P2RY12-14 GPR87 (UDP-Glucose)
Nu-019	Q9H244	Nucleotide-like	Purinoceptor P2RY12-14 GPR87 (UDP-Glucose)
Nu-019	Q9EPX4	Nucleotide-like	Purinoceptor P2RY12-14 GPR87 (UDP-Glucose)
Nu-019	Q95KC3	Nucleotide-like	Purinoceptor P2RY12-14 GPR87 (UDP-Glucose)
Nu-020	Q8BFQ3	Nucleotide-like	P2RY5
Nu-020	O46685	Nucleotide-like	P2RY5
Nu-020	Q15743	Nucleotide-like	P2RY5
Nu-021	Q6IYF8	Nucleotide-like	P2RY1
Nu-021	Q6Y1R5	Nucleotide-like	P2RY1
Nu-021	Q96P68	Nucleotide-like	P2RY1
Nu-022	A6QLE7	Nucleotide-like	P2RY5
Nu-022	Q9Y2T5	Nucleotide-like	P2RY5
Nu-022	P0C5J4	Nucleotide-like	P2RY5
Nu-023	Q8BG55	Nucleotide-like	Purinoceptor P2RY12-14 GPR87 (UDP-Glucose)
Nu-023	Q3ZBK9	Nucleotide-like	Purinoceptor P2RY12-14 GPR87 (UDP-Glucose)
Nu-023	O14626	Nucleotide-like	Purinoceptor P2RY12-14 GPR87 (UDP-Glucose)
Nu-024	Q99678	Nucleotide-like	P2RY5
Nu-024	Q8BYC4	Nucleotide-like	P2RY5
Nu-025	Q98907	Nucleotide-like	P2RY3and6
Nu-025	O93361	Nucleotide-like	P2RY3and6
Nu-026	Q61618	Nucleotide-like	Adenosine type 3
Nu-026	P28647	Nucleotide-like	Adenosine type 3
Nu-027	Q99MT7	Nucleotide-like	Purinoceptor P2RY12-14 GPR87 (UDP-Glucose)
Nu-027	Q9BY21	Nucleotide-like	Purinoceptor P2RY12-14 GPR87 (UDP-Glucose)
Nu-028	Q8BLG2	Nucleotide-like	P2RY9
Nu-028	Q99677	Nucleotide-like	P2RY9
Nu-029	Q99679	Nucleotide-like	P2RY5
Nu-029	Q8BX79	Nucleotide-like	P2RY5
Nu-030	Q8BZL4	Nucleotide-like	Purinoceptor P2RY12-14 GPR87 (UDP-Glucose)
Nu-030	Q99680	Nucleotide-like	Purinoceptor P2RY12-14 GPR87 (UDP-Glucose)
Ol-001	Q60892	Olfactory	Olfactory 107
Ol-001	P23265	Olfactory	Olfactory 108

Ol-001	Q8NGA2	Olfactory	Olfactory 71
Ol-001	Q95157	Olfactory	Olfactory 71
Ol-001	O76100	Olfactory	Olfactory 71
Ol-001	Q9JHB2	Olfactory	Olfactory 71
Ol-001	P35898	Olfactory	Olfactory 71
Ol-001	O14581	Olfactory	Olfactory 71
Ol-001	Q15622	Olfactory	Olfactory 71
Ol-002	Q8NHC7	Olfactory	Olfactory 183
Ol-002	Q9UGF5	Olfactory	Olfactory 201
Ol-002	Q8NGZ2	Olfactory	Olfactory 232
Ol-002	Q8NHC6	Olfactory	Olfactory 232
Ol-002	Q96R54	Olfactory	Olfactory 293
Ol-002	A6ND48	Olfactory	Olfactory 86
Ol-002	Q8NHC5	Olfactory	Olfactory 86
Ol-003	Q6IFG1	Olfactory	Olfactory 240
Ol-003	Q96RD3	Olfactory	Olfactory 240
Ol-003	Q8NGH9	Olfactory	Olfactory 240
Ol-003	Q9H346	Olfactory	Olfactory 241
Ol-003	P0C646	Olfactory	Olfactory 241
Ol-003	Q8NH55	Olfactory	Olfactory 246
Ol-003	Q8NH60	Olfactory	Olfactory 246
Ol-003	Q8NH57	Olfactory	Olfactory II fam 12 / MOR250
Ol-004	Q8VFB9	Olfactory	Olfactory 143
Ol-004	A6NDH6	Olfactory	Olfactory 185
Ol-004	Q8VEX6	Olfactory	Olfactory 185
Ol-004	A6NKK0	Olfactory	Olfactory 185
Ol-004	A6NHG9	Olfactory	Olfactory 279
Ol-004	Q8NGV7	Olfactory	Olfactory 279
Ol-004	Q8NGV6	Olfactory	Olfactory 279
Ol-004	Q8VEX5	Olfactory	Olfactory 279
Ol-005	Q9H341	Olfactory	Olfactory 133
Ol-005	Q8NH63	Olfactory	Olfactory 242
Ol-005	Q8NGK1	Olfactory	Olfactory 247
Ol-005	Q8NGJ5	Olfactory	Olfactory 247
Ol-005	Q8NH59	Olfactory	Olfactory 247
Ol-005	Q8NGK0	Olfactory	Olfactory II fam 10 / MOR263-269
Ol-006	Q8NGP6	Olfactory	Olfactory 225
Ol-006	Q8NGP3	Olfactory	Olfactory 225
Ol-006	Q8NGP4	Olfactory	Olfactory 225
Ol-006	Q8VFL5	Olfactory	Olfactory 226
Ol-006	Q8NGE7	Olfactory	Olfactory 290
Ol-006	Q96RB7	Olfactory	Olfactory 60
Ol-007	Q8NG78	Olfactory	Olfactory 147
Ol-007	Q15617	Olfactory	Olfactory 147
Ol-007	P34983	Olfactory	Olfactory 147

Ol-007	Q60884	Olfactory	Olfactory 147
Ol-007	Q60895	Olfactory	Olfactory 307
Ol-007	Q15614	Olfactory	Olfactory 312
Ol-008	P35896	Olfactory	Olfactory 70
Ol-008	P34987	Olfactory	Olfactory 70
Ol-008	Q7TRF3	Olfactory	Olfactory 70
Ol-008	Q96RA2	Olfactory	Olfactory 71
Ol-008	Q8NG98	Olfactory	Olfactory 71
Ol-009	Q8NH56	Olfactory	Olfactory II fam 12 / MOR250
Ol-009	Q8NGF1	Olfactory	Olfactory II fam 12 / MOR250
Ol-009	Q8NH53	Olfactory	Olfactory II fam 12 / MOR250
Ol-009	Q8NGI2	Olfactory	Olfactory II fam 12 / MOR250
Ol-009	Q8NGI0	Olfactory	Olfactory II fam 12 / MOR250
Ol-010	Q8NGI4	Olfactory	Olfactory 19
Ol-010	Q8NGE8	Olfactory	Olfactory 19
Ol-010	Q8NGI6	Olfactory	Olfactory 19
Ol-010	Q8NGN0	Olfactory	Olfactory 297
Ol-010	Q8NGJ1	Olfactory	Olfactory 92
Ol-011	Q9Y5P0	Olfactory	Olfactory 244
Ol-011	Q9Y5P1	Olfactory	Olfactory 244
Ol-011	Q9H339	Olfactory	Olfactory 244
Ol-011	Q9H340	Olfactory	Olfactory 244
Ol-011	Q9H2C8	Olfactory	Olfactory 247
Ol-012	Q96R47	Olfactory	Olfactory II fam 3 / MOR255
Ol-012	Q6IF42	Olfactory	Olfactory II fam 3 / MOR255
Ol-012	Q8NGT9	Olfactory	Olfactory II fam 3 / MOR255
Ol-012	Q96R48	Olfactory	Olfactory II fam 3 / MOR255
Ol-012	Q8NGT7	Olfactory	Olfactory II fam 3 / MOR255
Ol-013	Q60881	Olfactory	Olfactory 219
Ol-013	Q8NGF8	Olfactory	Olfactory 219
Ol-013	Q8NH49	Olfactory	Olfactory 25
Ol-013	Q8NGF9	Olfactory	Olfactory 25
Ol-013	Q8NH73	Olfactory	Olfactory 314
Ol-013	Q8NGB4	Olfactory	Olfactory 314
Ol-013	Q8NGL7	Olfactory	Olfactory 314
Ol-014	Q8NH43	Olfactory	Olfactory 118
Ol-014	Q8NGD5	Olfactory	Olfactory II fam 8 / MOR161-171
Ol-014	Q8NGC6	Olfactory	Olfactory II fam 8 / MOR161-171
Ol-014	Q96R72	Olfactory	Olfactory II fam 8 / MOR161-171
Ol-014	Q8NGD2	Olfactory	Olfactory II fam 8 / MOR161-171
Ol-014	Q8NGD4	Olfactory	Olfactory II fam 8 / MOR161-171
Ol-015	Q9H207	Olfactory	Olfactory 125
Ol-015	Q9H208	Olfactory	Olfactory 125
Ol-015	Q96KK4	Olfactory	Olfactory 149
Ol-015	Q8NGE5	Olfactory	Olfactory 284

Ol-015	Q9H209	Olfactory	Olfactory 300
Ol-015	Q8NH19	Olfactory	Olfactory 51
Ol-016	Q8VGS3	Olfactory	Olfactory 28
Ol-016	Q8NGP9	Olfactory	Olfactory 28
Ol-016	Q60894	Olfactory	Olfactory 291
Ol-016	Q8NGR4	Olfactory	Olfactory 39
Ol-016	Q8N127	Olfactory	Olfactory 54
Ol-017	Q8NH80	Olfactory	Olfactory II fam 6 / MOR103-105,107-119
Ol-017	Q60888	Olfactory	Olfactory II fam 6 / MOR103-105,107-119
Ol-017	Q60887	Olfactory	Olfactory II fam 6 / MOR103-105,107-119
Ol-017	Q8NGN7	Olfactory	Olfactory II fam 6 / MOR103-105,107-119
Ol-017	Q8VEY3	Olfactory	Olfactory II fam 6 / MOR103-105,107-119
Ol-018	Q8VG03	Olfactory	Olfactory II fam 4 / MOR225-248
Ol-018	Q8VFD3	Olfactory	Olfactory II fam 4 / MOR225-248
Ol-018	Q8VEW5	Olfactory	Olfactory II fam 4 / MOR225-248
Ol-018	Q8VFD1	Olfactory	Olfactory II fam 4 / MOR225-248
Ol-018	Q8VFD2	Olfactory	Olfactory II fam 4 / MOR225-248
Ol-019	Q96RD2	Olfactory	Olfactory 241
Ol-019	Q8NGF0	Olfactory	Olfactory 241
Ol-019	Q8NGJ2	Olfactory	Olfactory 304
Ol-019	Q8NGK2	Olfactory	Olfactory 91
Ol-020	Q8NH48	Olfactory	Olfactory 226
Ol-020	Q96R08	Olfactory	Olfactory 226
Ol-020	Q96R09	Olfactory	Olfactory 226
Ol-020	Q8NGF7	Olfactory	Olfactory 226
Ol-021	Q8VFC9	Olfactory	Olfactory 180
Ol-021	Q8VG44	Olfactory	Olfactory 180
Ol-021	Q8VG43	Olfactory	Olfactory 180
Ol-021	Q8VG06	Olfactory	Olfactory II fam 4 / MOR225-248
Ol-022	Q8NH94	Olfactory	Olfactory 257
Ol-022	Q8NH93	Olfactory	Olfactory 257
Ol-022	Q15612	Olfactory	Olfactory 258
Ol-022	Q8NGR8	Olfactory	Olfactory 259
Ol-023	Q8NGM8	Olfactory	Olfactory 129
Ol-023	Q8NH79	Olfactory	Olfactory 129
Ol-023	Q8NGC5	Olfactory	Olfactory 129
Ol-023	Q8NH40	Olfactory	Olfactory 177
Ol-024	Q8NH54	Olfactory	Olfactory 15
Ol-024	Q8NGH8	Olfactory	Olfactory 15
Ol-024	P0C7T3	Olfactory	Olfactory 15
Ol-024	Q8NGH5	Olfactory	Olfactory 15
Ol-025	Q5TZ20	Olfactory	Olfactory 106
Ol-025	Q8NGZ4	Olfactory	Olfactory 106
Ol-025	Q8N628	Olfactory	Olfactory 194
Ol-025	Q8NGZ5	Olfactory	Olfactory 20

Ol-026	P59922	Olfactory	Olfactory 106
Ol-026	Q60883	Olfactory	Olfactory 106
Ol-026	Q8NGV0	Olfactory	Olfactory 106
Ol-026	Q8NGU4	Olfactory	Olfactory 295
Ol-027	Q8NGR9	Olfactory	Olfactory 134
Ol-027	Q8NGS0	Olfactory	Olfactory 134
Ol-027	Q8NH06	Olfactory	Olfactory 145
Ol-027	O60431	Olfactory	Olfactory 71
Ol-027	Q8VFM9	Olfactory	Olfactory 71
Ol-027	Q8NGA1	Olfactory	Olfactory 71
Ol-028	O95221	Olfactory	Olfactory 193
Ol-028	Q8NGC0	Olfactory	Olfactory 211
Ol-028	Q8VFK7	Olfactory	Olfactory 226
Ol-028	Q8NGF4	Olfactory	Olfactory 226
Ol-028	Q9UGF6	Olfactory	Olfactory 234
Ol-029	O43749	Olfactory	Olfactory 134
Ol-029	Q96R84	Olfactory	Olfactory 134
Ol-029	P23266	Olfactory	Olfactory 134
Ol-029	Q15619	Olfactory	Olfactory 71
Ol-029	Q8NHA8	Olfactory	Olfactory 71
Ol-030	Q96R67	Olfactory	Olfactory 220
Ol-030	Q8NGM1	Olfactory	Olfactory 25
Ol-030	A6NMZ5	Olfactory	Olfactory 25
Ol-030	Q8NGP0	Olfactory	Olfactory 25
Ol-030	A6NHA9	Olfactory	Olfactory 90
Ol-031	Q96RD1	Olfactory	Olfactory 229
Ol-031	A6NL08	Olfactory	Olfactory 320
Ol-031	A6NCV1	Olfactory	Olfactory 320
Ol-031	Q9NZP0	Olfactory	Olfactory 320
Ol-032	Q60890	Olfactory	Olfactory 105
Ol-032	P58173	Olfactory	Olfactory 105
Ol-032	Q9GZK3	Olfactory	Olfactory 190
Ol-032	O76000	Olfactory	Olfactory 192
Ol-032	Q5JQS5	Olfactory	Olfactory 204
Ol-033	Q8NGC1	Olfactory	Olfactory 117
Ol-033	Q8NGC9	Olfactory	Olfactory 206
Ol-033	Q8NGC8	Olfactory	Olfactory 206
Ol-033	Q8NGC7	Olfactory	Olfactory 235
Ol-033	Q8NGX0	Olfactory	Olfactory 316
Ol-034	Q8NGQ2	Olfactory	Olfactory 103
Ol-034	Q8NGW1	Olfactory	Olfactory 103
Ol-034	Q6IFH4	Olfactory	Olfactory 103
Ol-034	P34986	Olfactory	Olfactory 138
Ol-034	O95007	Olfactory	Olfactory 156
Ol-035	Q8NGL6	Olfactory	Olfactory 25

Ol-035	Q8NGB2	Olfactory	Olfactory 25
Ol-035	Q6IF82	Olfactory	Olfactory 25
Ol-035	Q8NGN8	Olfactory	Olfactory 288
Ol-035	Q8NH72	Olfactory	Olfactory 45
Ol-036	A6NJZ3	Olfactory	Olfactory 320
Ol-036	A6NM76	Olfactory	Olfactory 320
Ol-036	A6NIJ9	Olfactory	Olfactory 77
Ol-036	A6NF89	Olfactory	Olfactory 77
Ol-037	Q60889	Olfactory	Olfactory 109
Ol-037	O95222	Olfactory	Olfactory 156
Ol-037	P23270	Olfactory	Olfactory 156
Ol-037	Q8NGX8	Olfactory	Olfactory 187
Ol-037	Q8NGX9	Olfactory	Olfactory 252
Ol-038	Q9Z1V0	Olfactory	Olfactory 216
Ol-038	Q8NGN1	Olfactory	Olfactory 216
Ol-038	Q8NGE2	Olfactory	Olfactory 272
Ol-038	Q8NGE1	Olfactory	Olfactory 272
Ol-039	Q8VGI5	Olfactory	Olfactory 202
Ol-039	Q8VEZ0	Olfactory	Olfactory II fam 4 / MOR225-248
Ol-039	Q8WZ94	Olfactory	Olfactory II fam 4 / MOR225-248
Ol-039	Q8VG42	Olfactory	Olfactory II fam 4 / MOR225-248
Ol-040	Q60880	Olfactory	Olfactory 18
Ol-040	Q8VES2	Olfactory	Olfactory 18
Ol-040	Q8VF13	Olfactory	Olfactory 18
Ol-040	Q8VFL9	Olfactory	Olfactory 18
Ol-041	Q8NGY1	Olfactory	Olfactory 215
Ol-041	P30954	Olfactory	Olfactory 215
Ol-041	Q8NHC4	Olfactory	Olfactory 215
Ol-041	Q8NGY7	Olfactory	Olfactory 215
Ol-042	Q8NGL1	Olfactory	Olfactory 26
Ol-042	Q8NGL4	Olfactory	Olfactory 287
Ol-042	Q8NGL3	Olfactory	Olfactory 47
Ol-042	Q8NGK9	Olfactory	Olfactory 47
Ol-043	P34985	Olfactory	Olfactory 228
Ol-043	Q15620	Olfactory	Olfactory 68
Ol-043	Q8NGG6	Olfactory	Olfactory 68
Ol-043	Q60882	Olfactory	Olfactory 68
Ol-044	Q7TR96	Olfactory	Olfactory 267
Ol-044	Q8NGQ1	Olfactory	Olfactory 29
Ol-044	P0C7N8	Olfactory	Olfactory 313
Ol-044	Q8NH87	Olfactory	Olfactory 313
Ol-045	Q6IEY1	Olfactory	Olfactory 218
Ol-045	O95013	Olfactory	Olfactory 218
Ol-045	Q8NGB9	Olfactory	Olfactory 283
Ol-045	Q8NGB8	Olfactory	Olfactory II fam 7 / MOR139-155

Ol-046	Q8N349	Olfactory	Olfactory 16
Ol-046	Q8NG80	Olfactory	Olfactory 16
Ol-046	Q8NH16	Olfactory	Olfactory 16
Ol-046	Q8NGZ0	Olfactory	Olfactory 16
Ol-047	P34984	Olfactory	Olfactory II fam 3 / MOR255
Ol-047	Q96R45	Olfactory	Olfactory II fam 3 / MOR255
Ol-047	O95047	Olfactory	Olfactory II fam 3 / MOR255
Ol-047	A4D2G3	Olfactory	Olfactory II fam 3 / MOR255
Ol-048	P30955	Olfactory	Olfactory 71
Ol-048	Q9TQX4	Olfactory	Olfactory 71
Ol-048	Q9TU94	Olfactory	Olfactory 71
Ol-048	Q9TUA2	Olfactory	Olfactory 71
Ol-048	Q9TUA9	Olfactory	Olfactory 71
Ol-048	Q8WZA6	Olfactory	Olfactory II fam 1 / MOR125-138,156
Ol-048	P30953	Olfactory	Olfactory II fam 1 / MOR125-138,156
Ol-048	P47887	Olfactory	Olfactory II fam 1 / MOR125-138,156
Ol-049	Q9TU88	Olfactory	Olfactory II fam 2 / MOR256-262,270-285
Ol-049	Q9TUA0	Olfactory	Olfactory II fam 2 / MOR256-262,270-285
Ol-049	P47883	Olfactory	Olfactory II fam 2 / MOR256-262,270-285
Ol-049	P47893	Olfactory	Olfactory II fam 2 / MOR256-262,270-285
Ol-049	P47888	Olfactory	Olfactory II fam 2 / MOR256-262,270-285
Ol-049	Q9TU97	Olfactory	Olfactory II fam 2 / MOR256-262,270-285
Ol-049	Q60891	Olfactory	Olfactory II fam 2 / MOR256-262,270-285
Ol-050	Q8NG81	Olfactory	Olfactory 136
Ol-050	A3KFT3	Olfactory	Olfactory 136
Ol-050	Q8NHA4	Olfactory	Olfactory 136
Ol-050	Q8NG83	Olfactory	Olfactory 136
Ol-050	Q96R27	Olfactory	Olfactory 136
Ol-050	Q96R28	Olfactory	Olfactory 136
Ol-051	Q8NGT0	Olfactory	Olfactory 122
Ol-051	Q8NGS8	Olfactory	Olfactory 122
Ol-051	Q8NGS9	Olfactory	Olfactory 122
Ol-051	Q8NGT2	Olfactory	Olfactory 263
Ol-051	Q8NGV5	Olfactory	Olfactory 37
Ol-052	Q8NGN2	Olfactory	Olfactory 269
Ol-052	Q8NGN6	Olfactory	Olfactory II fam 6 / MOR103-105,107-119
Ol-052	Q8NGN4	Olfactory	Olfactory II fam 6 / MOR103-105,107-119
Ol-052	Q8NGN5	Olfactory	Olfactory II fam 6 / MOR103-105,107-119
Ol-052	Q8NGN3	Olfactory	Olfactory II fam 6 / MOR103-105,107-119
Ol-053	O88628	Olfactory	Olfactory 174
Ol-053	Q8VBV9	Olfactory	Olfactory 174
Ol-053	Q9H255	Olfactory	Olfactory 174
Ol-053	Q8NGF3	Olfactory	Olfactory 248
Ol-053	Q8TCB6	Olfactory	Olfactory 75
Ol-054	Q8VGI1	Olfactory	Olfactory 71

Ol-054	P23274	Olfactory	Olfactory 71
Ol-054	P70526	Olfactory	Olfactory 71
Ol-054	P23272	Olfactory	Olfactory 71
Ol-055	Q8NH04	Olfactory	Olfactory 34
Ol-055	P0C7T2	Olfactory	Olfactory 34
Ol-055	Q8NHC8	Olfactory	Olfactory 35
Ol-055	O43869	Olfactory	Olfactory 35
Ol-056	Q96RC9	Olfactory	Olfactory 64
Ol-056	Q60886	Olfactory	Olfactory 64
Ol-056	Q8NGG8	Olfactory	Olfactory 64
Ol-056	Q96RD0	Olfactory	Olfactory 64
Ol-057	Q8N162	Olfactory	Olfactory 82
Ol-057	Q8N0Y5	Olfactory	Olfactory 82
Ol-057	Q8NGG4	Olfactory	Olfactory 82
Ol-057	Q8N146	Olfactory	Olfactory 82
Ol-058	P0C623	Olfactory	Olfactory 297
Ol-058	Q8NGB6	Olfactory	Olfactory 297
Ol-058	Q8NH05	Olfactory	Olfactory 297
Ol-058	Q8NGD0	Olfactory	Olfactory 297
Ol-059	Q8NGA6	Olfactory	Olfactory 37
Ol-059	Q9Y4A9	Olfactory	Olfactory 37
Ol-059	O60403	Olfactory	Olfactory 37
Ol-060	Q9Y3N9	Olfactory	Olfactory 189
Ol-060	Q7Z3T1	Olfactory	Olfactory 73
Ol-060	Q8NHA6	Olfactory	Olfactory 73
Ol-061	Q9UKL2	Olfactory	Olfactory 120
Ol-061	A6NMU1	Olfactory	Olfactory 120
Ol-061	Q9H2C5	Olfactory	Olfactory 200
Ol-062	Q8VG09	Olfactory	Olfactory II fam 4 / MOR225-248
Ol-062	Q8VG04	Olfactory	Olfactory II fam 4 / MOR225-248
Ol-062	Q8WZ92	Olfactory	Olfactory II fam 4 / MOR225-248
Ol-063	Q8NGS1	Olfactory	Olfactory 71
Ol-063	Q8NGS2	Olfactory	Olfactory 71
Ol-063	Q8VGK5	Olfactory	Olfactory 87
Ol-064	O60404	Olfactory	Olfactory 37
Ol-064	Q8VBW9	Olfactory	Olfactory 37
Ol-064	Q8NGA5	Olfactory	Olfactory 37
Ol-065	Q8NH70	Olfactory	Olfactory 288
Ol-065	P0C604	Olfactory	Olfactory 288
Ol-065	Q8NH83	Olfactory	Olfactory 288
Ol-066	Q8NGC4	Olfactory	Olfactory 269
Ol-066	Q8NGC3	Olfactory	Olfactory 269
Ol-066	Q8NH81	Olfactory	Olfactory 269
Ol-067	Q8NH76	Olfactory	Olfactory 158
Ol-067	Q8NGI3	Olfactory	Olfactory 88

Ol-067	Q8NGI1	Olfactory	Olfactory 88
Ol-068	A6NGY5	Olfactory	Olfactory 172
Ol-068	Q8NGJ9	Olfactory	Olfactory 21
Ol-068	Q8NH61	Olfactory	Olfactory 247
Ol-069	Q8NGG5	Olfactory	Olfactory 113
Ol-069	Q8NH51	Olfactory	Olfactory 57
Ol-069	Q8NH50	Olfactory	Olfactory 59
Ol-070	Q8NGD3	Olfactory	Olfactory II fam 8 / MOR161-171
Ol-070	Q8NH42	Olfactory	Olfactory II fam 8 / MOR161-171
Ol-070	Q8NH41	Olfactory	Olfactory II fam 8 / MOR161-171
Ol-071	Q8NGY2	Olfactory	Olfactory 251
Ol-071	Q8NGW6	Olfactory	Olfactory 251
Ol-071	Q8NGY3	Olfactory	Olfactory 251
Ol-072	Q8NGY0	Olfactory	Olfactory 210
Ol-072	Q8NGX3	Olfactory	Olfactory 210
Ol-072	Q8NGX6	Olfactory	Olfactory 214
Ol-073	Q8NGT5	Olfactory	Olfactory 129
Ol-073	Q8NGU2	Olfactory	Olfactory 129
Ol-073	Q8NGU1	Olfactory	Olfactory 129
Ol-074	Q8NGQ5	Olfactory	Olfactory 18
Ol-074	Q8NGE9	Olfactory	Olfactory 18
Ol-074	Q8NGQ6	Olfactory	Olfactory 18
Ol-075	Q8NGQ4	Olfactory	Olfactory 227
Ol-075	Q8NGF6	Olfactory	Olfactory II fam 3 / MOR255
Ol-075	Q8NGI7	Olfactory	Olfactory II fam 3 / MOR255
Ol-076	Q8NGP2	Olfactory	Olfactory 27
Ol-076	Q8NGG0	Olfactory	Olfactory 27
Ol-076	Q8NGG1	Olfactory	Olfactory 27
Ol-077	Q8NGG7	Olfactory	Olfactory 226
Ol-077	Q8NGM9	Olfactory	Olfactory 226
Ol-077	Q60893	Olfactory	Olfactory 226
Ol-078	Q8NGK3	Olfactory	Olfactory 22
Ol-078	Q8NGK4	Olfactory	Olfactory 22
Ol-078	Q8NGK5	Olfactory	Olfactory 89
Ol-079	Q8NGG3	Olfactory	Olfactory 18
Ol-079	Q8NGG2	Olfactory	Olfactory 18
Ol-079	Q8NG75	Olfactory	Olfactory 18
Ol-080	Q8IXE1	Olfactory	Olfactory 297
Ol-080	Q8N0Y3	Olfactory	Olfactory 297
Ol-080	Q8NGD1	Olfactory	Olfactory 297
Ol-081	Q8NG95	Olfactory	Olfactory 71
Ol-081	Q8NGA0	Olfactory	Olfactory 71
Ol-081	Q8NG99	Olfactory	Olfactory 71
Ol-082	P23271	Olfactory	Olfactory 71
Ol-082	P23273	Olfactory	Olfactory 71

Ol-082	P23269	Olfactory	Olfactory 71
Ol-083	Q9TU92	Olfactory	Olfactory 319
Ol-083	Q9TUA7	Olfactory	Olfactory 319
Ol-083	Q9Y585	Olfactory	Olfactory 319
Ol-083	Q9P1Q5	Olfactory	Olfactory 319
Ol-084	A6NET4	Olfactory	Olfactory 142
Ol-084	Q8NHB7	Olfactory	Olfactory 188
Ol-084	Q8NHB8	Olfactory	Olfactory 188
Ol-085	Q8NH02	Olfactory	Olfactory II fam 13 / MOR253
Ol-085	Q6IEZ7	Olfactory	Olfactory II fam 13 / MOR253
Ol-085	Q8NGZ9	Olfactory	Olfactory II fam 13 / MOR253
Ol-085	Q8NH00	Olfactory	Olfactory II fam 13 / MOR253
Ol-086	Q8NG97	Olfactory	Olfactory 23
Ol-086	A6NH00	Olfactory	Olfactory 38
Ol-086	Q8NG76	Olfactory	Olfactory 38
Ol-086	Q8NG77	Olfactory	Olfactory 38
Ol-087	O76001	Olfactory	Olfactory 191
Ol-087	O76002	Olfactory	Olfactory 191
Ol-087	Q9GZK6	Olfactory	Olfactory 191
Ol-088	Q96R30	Olfactory	Olfactory 38
Ol-088	Q8NHB1	Olfactory	Olfactory 38
Ol-088	Q8VGD6	Olfactory	Olfactory 38
Ol-089	Q8NH64	Olfactory	Olfactory 209
Ol-089	Q8NGJ6	Olfactory	Olfactory 209
Ol-089	Q8NGJ7	Olfactory	Olfactory 209
Ol-090	Q7TS48	Olfactory	Olfactory 280
Ol-090	Q8VGQ7	Olfactory	Olfactory 280
Ol-090	A6NMS3	Olfactory	Olfactory 80
Ol-091	Q8NH85	Olfactory	Olfactory 224
Ol-091	P0C617	Olfactory	Olfactory 224
Ol-091	Q8VGS1	Olfactory	Olfactory 224
Ol-092	Q8NH01	Olfactory	Olfactory 315
Ol-092	Q6IF00	Olfactory	Olfactory 33
Ol-092	Q8NGX2	Olfactory	Olfactory 33
Ol-093	O60412	Olfactory	Olfactory 217
Ol-093	O76099	Olfactory	Olfactory 71
Ol-094	Q8NH69	Olfactory	Olfactory II fam 5 / MOR172-224,249,254
Ol-094	Q95155	Olfactory	Olfactory II fam 5 / MOR172-224,249,254
Ol-095	Q8VFK1	Olfactory	Olfactory 268
Ol-095	Q9QY00	Olfactory	Olfactory 61
Ol-095	Q8VFK2	Olfactory	Olfactory 61
Ol-096	Q8NGR3	Olfactory	Olfactory 257
Ol-096	Q8NGR2	Olfactory	Olfactory 96
Ol-096	Q8NGR5	Olfactory	Olfactory 96
Ol-097	Q8NGY9	Olfactory	Olfactory 16

Ol-097	Q8NG85	Olfactory	Olfactory 16
Ol-097	Q8NG84	Olfactory	Olfactory 301
Ol-098	Q8VG08	Olfactory	Olfactory II fam 4 / MOR225-248
Ol-098	Q8VG05	Olfactory	Olfactory II fam 4 / MOR225-248
Ol-098	Q8VG07	Olfactory	Olfactory II fam 4 / MOR225-248
Ol-099	P58182	Olfactory	Olfactory 281
Ol-099	Q9UGF7	Olfactory	Olfactory 281
Ol-100	P0C628	Olfactory	Olfactory 79
Ol-100	Q9NZP5	Olfactory	Olfactory 79
Ol-101	A6NDL8	Olfactory	Olfactory 115
Ol-101	Q9NZP2	Olfactory	Olfactory 320
Ol-102	Q8NGS7	Olfactory	Olfactory 36
Ol-102	Q9NQN1	Olfactory	Olfactory 95
Ol-103	Q9H344	Olfactory	Olfactory 242
Ol-103	Q9H343	Olfactory	Olfactory 243
Ol-104	Q8NGH3	Olfactory	Olfactory 119
Ol-104	Q9H210	Olfactory	Olfactory 238
Ol-105	Q9H205	Olfactory	Olfactory 14
Ol-105	A6NM03	Olfactory	Olfactory 14
Ol-106	Q8WZ84	Olfactory	Olfactory 63
Ol-106	Q9GZM6	Olfactory	Olfactory 63
Ol-107	Q95154	Olfactory	Olfactory 49
Ol-107	Q13606	Olfactory	Olfactory 49
Ol-108	Q8VGR8	Olfactory	Olfactory 58
Ol-108	Q8NH18	Olfactory	Olfactory 58
Ol-109	Q8VGI6	Olfactory	Olfactory II fam 4 / MOR225-248
Ol-109	Q8VGI4	Olfactory	Olfactory II fam 4 / MOR225-248
Ol-110	Q8VEW6	Olfactory	Olfactory II fam 4 / MOR225-248
Ol-110	Q8VG13	Olfactory	Olfactory II fam 4 / MOR225-248
Ol-111	Q8VFD0	Olfactory	Olfactory II fam 4 / MOR225-248
Ol-111	Q8VG02	Olfactory	Olfactory II fam 4 / MOR225-248
Ol-112	Q8VFX2	Olfactory	Olfactory 213
Ol-112	A6NL26	Olfactory	Olfactory 226
Ol-113	Q8NGI8	Olfactory	Olfactory 101
Ol-113	Q8VFV4	Olfactory	Olfactory 101
Ol-114	P0C626	Olfactory	Olfactory 62
Ol-114	Q8VF76	Olfactory	Olfactory 62
Ol-115	Q8NH89	Olfactory	Olfactory 226
Ol-115	Q8NH90	Olfactory	Olfactory 226
Ol-116	P58181	Olfactory	Olfactory 178
Ol-116	Q8NH74	Olfactory	Olfactory 178
Ol-117	Q60878	Olfactory	Olfactory 25
Ol-117	Q8NH37	Olfactory	Olfactory 25
Ol-118	P23267	Olfactory	Olfactory 177
Ol-118	Q8NGZ6	Olfactory	Olfactory 262

Ol-119	Q8NGX5	Olfactory	Olfactory 215
Ol-119	Q6IF99	Olfactory	Olfactory 215
Ol-120	Q8NGS4	Olfactory	Olfactory 37
Ol-120	Q8NGT1	Olfactory	Olfactory 37
Ol-121	Q8NGS5	Olfactory	Olfactory 36
Ol-121	Q8NGS6	Olfactory	Olfactory 36
Ol-122	Q8NGS3	Olfactory	Olfactory 71
Ol-122	Q60879	Olfactory	Olfactory 71
Ol-123	Q8NGL9	Olfactory	Olfactory 17
Ol-123	Q6IEV9	Olfactory	Olfactory 17
Ol-124	Q8NGJ4	Olfactory	Olfactory 246
Ol-124	Q8NGJ3	Olfactory	Olfactory 246
Ol-125	Q8NGI9	Olfactory	Olfactory 18
Ol-125	Q8NGJ0	Olfactory	Olfactory 18
Ol-126	Q8NGH7	Olfactory	Olfactory II fam 12 / MOR250
Ol-126	Q8NGH6	Olfactory	Olfactory II fam 12 / MOR250
Ol-127	Q60885	Olfactory	Olfactory 154
Ol-127	Q8NGE3	Olfactory	Olfactory 154
Ol-128	Q8NGC2	Olfactory	Olfactory 212
Ol-128	P0C645	Olfactory	Olfactory 212
Ol-129	Q6IFN5	Olfactory	Olfactory 24
Ol-129	Q0VAX9	Olfactory	Olfactory 24
Ol-130	Q5JRS4	Olfactory	Olfactory 266
Ol-130	P0C629	Olfactory	Olfactory 266
Ol-131	Q8NGZ3	Olfactory	Olfactory 277
Ol-131	Q8NGR1	Olfactory	Olfactory 298
Ol-132	Q8NGY5	Olfactory	Olfactory 251
Ol-132	Q8NGY6	Olfactory	Olfactory 251
Ol-133	Q9GZK4	Olfactory	Olfactory 20
Ol-133	O95918	Olfactory	Olfactory 20
Ol-134	Q15615	Olfactory	Olfactory 182
Ol-134	P58180	Olfactory	Olfactory 40
Ol-135	O95371	Olfactory	Olfactory 159
Ol-135	P23275	Olfactory	Olfactory 159
Ol-136	P37072	Olfactory	Olfactory II fam 5 / MOR172-224,249,254
Ol-136	P37067	Olfactory	Olfactory II fam 5 / MOR172-224,249,254
Ol-136	P37069	Olfactory	Olfactory II fam 5 / MOR172-224,249,254
Ol-136	P37071	Olfactory	Olfactory II fam 5 / MOR172-224,249,254
Ol-136	P37070	Olfactory	Olfactory II fam 5 / MOR172-224,249,254
Ol-136	P37068	Olfactory	Olfactory II fam 5 / MOR172-224,249,254
Ol-137	Q9TUA6	Olfactory	Olfactory 78
Ol-137	P58170	Olfactory	Olfactory 78
Ol-137	P47884	Olfactory	Olfactory 78
Ol-137	Q9TU90	Olfactory	Olfactory 78
Ol-137	Q9TU95	Olfactory	Olfactory 78

Ol-138	Q8NH10	Olfactory	Olfactory 152
Ol-138	P0C7N1	Olfactory	Olfactory 152
Ol-138	Q8VGR9	Olfactory	Olfactory 152
Ol-138	P0C7N5	Olfactory	Olfactory 90
Ol-139	Q9TUA4	Olfactory	Olfactory II fam 2 / MOR256-262,270-285
Ol-139	Q9TU89	Olfactory	Olfactory II fam 2 / MOR256-262,270-285
Ol-139	P47881	Olfactory	Olfactory II fam 2 / MOR256-262,270-285
Ol-140	Q9TU93	Olfactory	Olfactory 317
Ol-140	Q9TUA8	Olfactory	Olfactory 317
Ol-140	P34982	Olfactory	Olfactory 317
Ol-141	B2RN74	Olfactory	Olfactory 235
Ol-141	Q8NG94	Olfactory	Olfactory 235
Ol-141	Q8NH07	Olfactory	Olfactory 235
Ol-142	Q13607	Olfactory	Olfactory 31
Ol-142	Q95156	Olfactory	Olfactory 31
Ol-142	O95006	Olfactory	Olfactory 31
Ol-143	Q8NGA8	Olfactory	Olfactory II fam 8 / MOR161-171
Ol-143	Q8NH21	Olfactory	Olfactory II fam 8 / MOR161-171
Ol-143	Q96R69	Olfactory	Olfactory II fam 8 / MOR161-171
Ol-144	Q8NGL0	Olfactory	Olfactory 286
Ol-144	Q8NGL2	Olfactory	Olfactory 286
Ol-145	P47890	Olfactory	Olfactory 134
Ol-145	Q9TU86	Olfactory	Olfactory 71
Ol-146	Q8NH67	Olfactory	Olfactory 285
Ol-146	Q8NGK6	Olfactory	Olfactory 285
Ol-147	Q8NH03	Olfactory	Olfactory II fam 13 / MOR253
Ol-147	Q8NGX1	Olfactory	Olfactory II fam 13 / MOR253
Ol-148	Q8VEW2	Olfactory	Olfactory II fam 4 / MOR225-248
Ol-148	Q8VF12	Olfactory	Olfactory II fam 4 / MOR225-248
Ol-149	Q8VF66	Olfactory	Olfactory II fam 4 / MOR225-248
Ol-149	Q8VF65	Olfactory	Olfactory II fam 4 / MOR225-248
Ol-150	Q8NGQ3	Olfactory	Olfactory 71
Ol-150	Q8NH92	Olfactory	Olfactory 71
Ol-151	Q6IEU7	Olfactory	Olfactory 60
Ol-151	Q8NGP8	Olfactory	Olfactory 60
OT-001	Q7TN49	Class A Orphan/other	Mas proto-oncogene and Mas-related (MRGs)
OT-001	Q91WW4	Class A Orphan/other	Mas proto-oncogene and Mas-related (MRGs)
OT-001	Q91ZC7	Class A Orphan/other	Mas proto-oncogene and Mas-related (MRGs)
OT-001	Q91ZC6	Class A Orphan/other	Mas proto-oncogene and Mas-related (MRGs)
OT-001	Q91WW5	Class A Orphan/other	Mas proto-oncogene and Mas-related (MRGs)
OT-002	Q3UG61	Class A Orphan/other	Mas proto-oncogene and Mas-related (MRGs)
OT-002	Q3UG50	Class A Orphan/other	Mas proto-oncogene and Mas-related (MRGs)
OT-002	Q91ZC1	Class A Orphan/other	Mas proto-oncogene and Mas-related (MRGs)
OT-002	Q3KNA1	Class A Orphan/other	Mas proto-oncogene and Mas-related (MRGs)
OT-003	Q91ZC5	Class A Orphan/other	Mas proto-oncogene and Mas-related (MRGs)

OT-003	Q91WW2	Class A Orphan/other	Mas proto-oncogene and Mas-related (MRGs)
OT-003	Q91WW3	Class A Orphan/other	Mas proto-oncogene and Mas-related (MRGs)
OT-003	Q91ZC4	Class A Orphan/other	Mas proto-oncogene and Mas-related (MRGs)
OT-004	Q96LB0	Class A Orphan/other	Mas proto-oncogene and Mas-related (MRGs)
OT-004	Q96LB2	Class A Orphan/other	Mas proto-oncogene and Mas-related (MRGs)
OT-004	Q96LA9	Class A Orphan/other	Mas proto-oncogene and Mas-related (MRGs)
OT-004	Q2LL16	Class A Orphan/other	Mas proto-oncogene and Mas-related (MRGs)
OT-005	Q924T9	Class A Orphan/other	GPR17
OT-005	Q9NS75	Class A Orphan/other	GPR17
OT-005	Q920A1	Class A Orphan/other	GPR17
OT-006	Q86SM5	Class A Orphan/other	Mas proto-oncogene and Mas-related (MRGs)
OT-006	Q7TN39	Class A Orphan/other	Mas proto-oncogene and Mas-related (MRGs)
OT-006	Q91ZB5	Class A Orphan/other	Mas proto-oncogene and Mas-related (MRGs)
OT-007	Q76JU8	Class A Orphan/other	Free fatty acid receptor 3
OT-007	Q8K3T4	Class A Orphan/other	Free fatty acid receptor 3
OT-007	Q76JU9	Class A Orphan/other	Free fatty acid receptor 3
OT-008	Q80SS6	Class A Orphan/other	G-protein coupled bile acid receptor
OT-008	Q862A9	Class A Orphan/other	G-protein coupled bile acid receptor
OT-008	Q80T02	Class A Orphan/other	G-protein coupled bile acid receptor
OT-009	B5X337	Class A Orphan/other	EBV-induced
OT-009	B0UXR0	Class A Orphan/other	EBV-induced
OT-009	A5PLE7	Class A Orphan/other	EBV-induced
OT-010	Q8C010	Class A Orphan/other	Other 519
OT-010	Q9BZJ8	Class A Orphan/other	Other 519
OT-010	Q91178	Class A Orphan/other	Other 519
OT-011	Q8VCK6	Class A Orphan/other	Free fatty acid receptor 3
OT-011	Q76EI6	Class A Orphan/other	Free fatty acid receptor 3
OT-011	O15552	Class A Orphan/other	Free fatty acid receptor 3
OT-012	Q9EPB7	Class A Orphan/other	GPR88
OT-012	Q9GZN0	Class A Orphan/other	GPR88
OT-012	Q9ESP4	Class A Orphan/other	GPR88
OT-013	Q9ES90	Class A Orphan/other	Other 519
OT-013	Q9HC97	Class A Orphan/other	Other 519
OT-014	Q9BZJ7	Class A Orphan/other	Other 519
OT-014	Q80UC6	Class A Orphan/other	Other 519
OT-015	Q91ZC0	Class A Orphan/other	Mas proto-oncogene and Mas-related (MRGs)
OT-015	Q91ZB9	Class A Orphan/other	Mas proto-oncogene and Mas-related (MRGs)
OT-016	Q7TN41	Class A Orphan/other	Mas proto-oncogene and Mas-related (MRGs)
OT-016	Q91ZB8	Class A Orphan/other	Mas proto-oncogene and Mas-related (MRGs)
OT-017	Q7TN40	Class A Orphan/other	Mas proto-oncogene and Mas-related (MRGs)
OT-017	Q91ZB7	Class A Orphan/other	Mas proto-oncogene and Mas-related (MRGs)
OT-018	Q7TN50	Class A Orphan/other	Mas proto-oncogene and Mas-related (MRGs)
OT-018	Q7TN51	Class A Orphan/other	Mas proto-oncogene and Mas-related (MRGs)
OT-019	Q7TN44	Class A Orphan/other	Mas proto-oncogene and Mas-related (MRGs)
OT-019	Q7TN45	Class A Orphan/other	Mas proto-oncogene and Mas-related (MRGs)

OT-020	Q862A8	Class A Orphan/other	G-protein coupled bile acid receptor
OT-020	Q8TDU6	Class A Orphan/other	G-protein coupled bile acid receptor
OT-021	Q8TDS7	Class A Orphan/other	Mas proto-oncogene and Mas-related (MRGs)
OT-021	Q6L786	Class A Orphan/other	Mas proto-oncogene and Mas-related (MRGs)
OT-022	Q8CIP3	Class A Orphan/other	Mas proto-oncogene and Mas-related (MRGs)
OT-022	Q8R4G1	Class A Orphan/other	Mas proto-oncogene and Mas-related (MRGs)
OT-023	Q86SM8	Class A Orphan/other	Mas proto-oncogene and Mas-related (MRGs)
OT-023	Q5U9D7	Class A Orphan/other	Mas proto-oncogene and Mas-related (MRGs)
OT-024	Q7TQN9	Class A Orphan/other	GPR139
OT-024	Q7Z601	Class A Orphan/other	GPR139
OT-025	Q76JV1	Class A Orphan/other	Free fatty acid receptor 3
OT-025	O14842	Class A Orphan/other	Free fatty acid receptor 3
OT-026	F8VQN3	Class A Orphan/other	GPR31
OT-026	O00270	Class A Orphan/other	GPR31
OT-027	A2ARI4	Class A Orphan/other	LGR like (hormone receptors) type 4 and 5
OT-027	O75473	Class A Orphan/other	LGR like (hormone receptors) type 4 and 5
OT-027	Q9HBX8	Class A Orphan/other	LGR like (hormone receptors) type 4 and 5
OT-027	B0BLW3	Class A Orphan/other	LGR like (hormone receptors) type 4 and 5
OT-027	Q3UVD5	Class A Orphan/other	LGR like (hormone receptors) type 4 and 5
OT-027	D4AC13	Class A Orphan/other	LGR like (hormone receptors) type 4 and 5
OT-027	Q9Z1P4	Class A Orphan/other	LGR like (hormone receptors) type 4 and 5
OT-027	Q9BXB1	Class A Orphan/other	LGR like (hormone receptors) type 4 and 5
OT-027	Q9Z2H4	Class A Orphan/other	LGR like (hormone receptors) type 4 and 5
OT-028	O88280	Class A Orphan/other	LGR like (hormone receptors) type 4 and 5
OT-028	O75093	Class A Orphan/other	LGR like (hormone receptors) type 4 and 5
OT-028	Q80TR4	Class A Orphan/other	LGR like (hormone receptors) type 4 and 5
OT-028	Q9WVB4	Class A Orphan/other	LGR like (hormone receptors) type 4 and 5
OT-028	Q9R1B9	Class A Orphan/other	LGR like (hormone receptors) type 4 and 5
OT-028	O94813	Class A Orphan/other	LGR like (hormone receptors) type 4 and 5
OT-028	O75094	Class A Orphan/other	LGR like (hormone receptors) type 4 and 5
OT-028	O88279	Class A Orphan/other	LGR like (hormone receptors) type 4 and 5
OT-029	Q5U9D9	Class A Orphan/other	Mas proto-oncogene and Mas-related (MRGs)
OT-029	Q4QXU4	Class A Orphan/other	Mas proto-oncogene and Mas-related (MRGs)
OT-029	Q4QXU2	Class A Orphan/other	Mas proto-oncogene and Mas-related (MRGs)
OT-029	Q96LB1	Class A Orphan/other	Mas proto-oncogene and Mas-related (MRGs)
OT-029	Q4QXU0	Class A Orphan/other	Mas proto-oncogene and Mas-related (MRGs)
OT-029	Q4QXU3	Class A Orphan/other	Mas proto-oncogene and Mas-related (MRGs)
OT-029	Q4QXU5	Class A Orphan/other	Mas proto-oncogene and Mas-related (MRGs)
OT-029	Q4QXX9	Class A Orphan/other	Mas proto-oncogene and Mas-related (MRGs)
OT-030	Q9I919	Class A Orphan/other	SREB
OT-030	P60894	Class A Orphan/other	SREB
OT-030	P60893	Class A Orphan/other	SREB
OT-030	P60895	Class A Orphan/other	SREB
OT-030	Q5RBG7	Class A Orphan/other	SREB
OT-031	P60019	Class A Orphan/other	GPR34

OT-031	Q9R1K6	Class A Orphan/other	GPR34
OT-031	Q3SAG9	Class A Orphan/other	GPR34
OT-031	Q9UPC5	Class A Orphan/other	GPR34
OT-031	Q6XCF2	Class A Orphan/other	GPR34
OT-032	Q8K1Z6	Class A Orphan/other	GPR18
OT-032	Q3T0E9	Class A Orphan/other	GPR18
OT-032	Q4R613	Class A Orphan/other	GPR18
OT-032	A1A5S3	Class A Orphan/other	GPR18
OT-032	Q14330	Class A Orphan/other	GPR18
OT-033	Q9I918	Class A Orphan/other	SREB
OT-033	Q9JJH2	Class A Orphan/other	SREB
OT-033	Q9NS66	Class A Orphan/other	SREB
OT-033	Q6PI62	Class A Orphan/other	SREB
OT-033	Q5E9H8	Class A Orphan/other	SREB
OT-034	Q9Y271	Class A Orphan/other	Cysteinyl leukotriene type 1
OT-034	Q99JA4	Class A Orphan/other	Cysteinyl leukotriene type 1
OT-034	Q2NNR5	Class A Orphan/other	Cysteinyl leukotriene type 1
OT-034	Q924T8	Class A Orphan/other	Cysteinyl leukotriene type 1
OT-035	Q3U6B2	Class A Orphan/other	EBV-induced
OT-035	Q1RMI1	Class A Orphan/other	EBV-induced
OT-035	D4A7K7	Class A Orphan/other	EBV-induced
OT-035	P32249	Class A Orphan/other	EBV-induced
OT-036	Q5NUL3	Class A Orphan/other	Other 512
OT-036	C8YUV0	Class A Orphan/other	Other 512
OT-036	Q7TMA4	Class A Orphan/other	Other 512
OT-036	Q2AC31	Class A Orphan/other	Other 512
OT-037	P11613	Class A Orphan/other	RDC1
OT-037	P25106	Class A Orphan/other	RDC1
OT-037	O89039	Class A Orphan/other	RDC1
OT-037	P56485	Class A Orphan/other	RDC1
OT-038	Q5U431	Class A Orphan/other	Other 511
OT-038	B2ZHY2	Class A Orphan/other	Other 511
OT-038	O43194	Class A Orphan/other	Other 511
OT-038	B4XF06	Class A Orphan/other	Other 511
OT-039	Q09QM4	Class A Orphan/other	GPR17
OT-039	Q13304	Class A Orphan/other	GPR17
OT-039	Q6NS65	Class A Orphan/other	GPR17
OT-040	P30554	Class A Orphan/other	Mas proto-oncogene and Mas-related (MRGs)
OT-040	P12526	Class A Orphan/other	Mas proto-oncogene and Mas-related (MRGs)
OT-040	P04201	Class A Orphan/other	Mas proto-oncogene and Mas-related (MRGs)
OT-041	P0C0W8	Class A Orphan/other	GPR139
OT-041	Q80UC8	Class A Orphan/other	GPR139
OT-041	Q6DWJ6	Class A Orphan/other	GPR139
OT-042	Q5QD04	Class A Orphan/other	GPR3, 6 and 12
OT-042	Q96RI9	Class A Orphan/other	GPR3, 6 and 12

OT-042	Q923Y6	Class A Orphan/other	GPR3, 6 and 12
OT-043	Q9JJH3	Class A Orphan/other	SREB
OT-043	Q9NS67	Class A Orphan/other	SREB
OT-043	O54897	Class A Orphan/other	SREB
OT-044	Q96AM1	Class A Orphan/other	Mas proto-oncogene and Mas-related (MRGs)
OT-044	P23749	Class A Orphan/other	Mas proto-oncogene and Mas-related (MRGs)
OT-044	Q8VCJ6	Class A Orphan/other	Mas proto-oncogene and Mas-related (MRGs)
OT-045	Q8TDV0	Class A Orphan/other	GPR151 like
OT-045	Q7TSN6	Class A Orphan/other	GPR151 like
OT-045	Q7TSN5	Class A Orphan/other	GPR151 like
OT-046	Q9BXC1	Class A Orphan/other	Other 519
OT-046	Q3U507	Class A Orphan/other	Other 519
OT-047	O14843	Class A Orphan/other	Free fatty acid receptor 3
OT-047	O15529	Class A Orphan/other	Free fatty acid receptor 3
OT-048	Q3UFD7	Class A Orphan/other	Free fatty acid receptor 3
OT-048	B2GV46	Class A Orphan/other	Free fatty acid receptor 3
OT-049	Q99MT8	Class A Orphan/other	Mas proto-oncogene and Mas-related (MRGs)
OT-049	Q7TN38	Class A Orphan/other	Mas proto-oncogene and Mas-related (MRGs)
Pe-001	P79234	Peptide	C5a anaphylatoxin
Pe-001	P30992	Peptide	C5a anaphylatoxin
Pe-001	O70129	Peptide	C5a anaphylatoxin
Pe-001	P79175	Peptide	C5a anaphylatoxin
Pe-001	P79240	Peptide	C5a anaphylatoxin
Pe-001	P21730	Peptide	C5a anaphylatoxin
Pe-001	Q9TUE1	Peptide	C5a anaphylatoxin
Pe-001	P79188	Peptide	C5a anaphylatoxin
Pe-002	P46663	Peptide	Bradykinin type B1
Pe-002	Q95L01	Peptide	Bradykinin type B1
Pe-002	Q8HZP2	Peptide	Bradykinin type B1
Pe-002	Q8HZN9	Peptide	Bradykinin type B1
Pe-002	Q3BCU0	Peptide	Bradykinin type B1
Pe-002	P48748	Peptide	Bradykinin type B1
Pe-002	Q9BDQ5	Peptide	Bradykinin type B1
Pe-002	Q8HZP1	Peptide	Bradykinin type B1
Pe-002	Q8HZP3	Peptide	Bradykinin type B1
Pe-003	Q90334	Peptide	Isotocin 8
Pe-003	P32306	Peptide	Mesotocin
Pe-003	Q90252	Peptide	Mesotocin
Pe-003	P97926	Peptide	Mesotocin
Pe-003	P56449	Peptide	Mesotocin
Pe-003	P56494	Peptide	Mesotocin
Pe-003	Q28756	Peptide	Mesotocin
Pe-003	P30559	Peptide	Mesotocin
Pe-003	P70536	Peptide	Mesotocin
Pe-004	P16177	Peptide	Neuromedin K (NK3)

Pe-004	O97512	Peptide	Neuromedin K (NK3)
Pe-004	P30098	Peptide	Neuromedin K (NK3)
Pe-004	P47937	Peptide	Neuromedin K (NK3)
Pe-004	P29371	Peptide	Neuromedin K (NK3)
Pe-004	P30974	Peptide	Tachykinin like 1
Pe-004	P30975	Peptide	Tachykinin like 2
Pe-005	P25025	Peptide	Interleukin-8 type B
Pe-005	P35344	Peptide	Interleukin-8 type B
Pe-005	Q28807	Peptide	Interleukin-8 type B
Pe-005	O97571	Peptide	Interleukin-8 type B
Pe-005	Q28519	Peptide	Interleukin-8 type B
Pe-005	Q28422	Peptide	Interleukin-8 type B
Pe-006	P51678	Peptide	C-C Chemokine type 3
Pe-006	O54814	Peptide	C-C Chemokine type 3
Pe-006	Q64H34	Peptide	C-C Chemokine type 3
Pe-006	Q9Z2I3	Peptide	C-C Chemokine type 3
Pe-006	Q89609	Peptide	C-C Chemokine type 3
Pe-007	Q865E5	Peptide	Melanocortin type 1
Pe-007	Q9TU05	Peptide	Melanocortin type 1
Pe-007	Q865E8	Peptide	Melanocortin type 1
Pe-007	P79166	Peptide	Melanocortin type 1
Pe-007	Q865E9	Peptide	Melanocortin type 1
Pe-008	Q9Z1S9	Peptide	Adrenocorticotropic hormone
Pe-008	P70115	Peptide	Adrenocorticotropic hormone
Pe-008	Q01718	Peptide	Adrenocorticotropic hormone
Pe-008	Q64326	Peptide	Adrenocorticotropic hormone
Pe-009	P49681	Peptide	Somatostatin type 5
Pe-009	P48145	Peptide	Somatostatin type 5
Pe-009	Q8MJV3	Peptide	Somatostatin type 5
Pe-009	Q56UD9	Peptide	Somatostatin type 5
Pe-010	Q66H29	Peptide	Melanin-concentrating hormone receptors
Pe-010	Q9UJ42	Peptide	Melanin-concentrating hormone receptors
Pe-010	Q3U3F9	Peptide	Melanin-concentrating hormone receptors
Pe-011	Q864F6	Peptide	Melanocortin type 1
Pe-011	Q864F4	Peptide	Melanocortin type 1
Pe-011	Q864F5	Peptide	Melanocortin type 1
Pe-012	Q864J4	Peptide	Melanocortin type 1
Pe-012	Q864J2	Peptide	Melanocortin type 1
Pe-012	Q01726	Peptide	Melanocortin type 1
Pe-012	Q864L0	Peptide	Melanocortin type 1
Pe-012	Q864J8	Peptide	Melanocortin type 1
Pe-012	Q9TUK4	Peptide	Melanocortin type 1
Pe-012	Q864G6	Peptide	Melanocortin type 1
Pe-012	Q864G3	Peptide	Melanocortin type 1
Pe-012	Q864J1	Peptide	Melanocortin type 1

Pe-012	Q864I3	Peptide	Melanocortin type 1
Pe-012	Q864I2	Peptide	Melanocortin type 1
Pe-012	Q864H7	Peptide	Melanocortin type 1
Pe-012	Q864G1	Peptide	Melanocortin type 1
Pe-012	Q864K9	Peptide	Melanocortin type 1
Pe-012	Q864K1	Peptide	Melanocortin type 1
Pe-012	Q864I7	Peptide	Melanocortin type 1
Pe-012	Q864H5	Peptide	Melanocortin type 1
Pe-012	Q864H9	Peptide	Melanocortin type 1
Pe-012	Q864K8	Peptide	Melanocortin type 1
Pe-012	Q864J5	Peptide	Melanocortin type 1
Pe-012	Q864I9	Peptide	Melanocortin type 1
Pe-012	Q864J6	Peptide	Melanocortin type 1
Pe-012	Q864I8	Peptide	Melanocortin type 1
Pe-012	Q864G9	Peptide	Melanocortin type 1
Pe-012	Q864K4	Peptide	Melanocortin type 1
Pe-012	Q864I4	Peptide	Melanocortin type 1
Pe-012	Q864I0	Peptide	Melanocortin type 1
Pe-012	Q864K2	Peptide	Melanocortin type 1
Pe-012	Q0Q460	Peptide	Melanocortin type 1
Pe-012	Q864K6	Peptide	Melanocortin type 1
Pe-012	Q864G4	Peptide	Melanocortin type 1
Pe-012	Q864K5	Peptide	Melanocortin type 1
Pe-012	Q864I6	Peptide	Melanocortin type 1
Pe-012	Q864H2	Peptide	Melanocortin type 1
Pe-012	Q864I5	Peptide	Melanocortin type 1
Pe-012	Q864H3	Peptide	Melanocortin type 1
Pe-012	Q864J3	Peptide	Melanocortin type 1
Pe-012	Q864I1	Peptide	Melanocortin type 1
Pe-012	Q864H4	Peptide	Melanocortin type 1
Pe-012	Q864K0	Peptide	Melanocortin type 1
Pe-012	Q864J7	Peptide	Melanocortin type 1
Pe-012	Q864G2	Peptide	Melanocortin type 1
Pe-012	Q864J9	Peptide	Melanocortin type 1
Pe-012	Q864H0	Peptide	Melanocortin type 1
Pe-012	Q864J0	Peptide	Melanocortin type 1
Pe-012	Q864K3	Peptide	Melanocortin type 1
Pe-012	Q864H6	Peptide	Melanocortin type 1
Pe-013	Q9N0W7	Peptide	Endothelin type B
Pe-013	P21451	Peptide	Endothelin type B
Pe-013	P35463	Peptide	Endothelin type B
Pe-013	Q90328	Peptide	Endothelin type B
Pe-013	P32940	Peptide	Endothelin type B
Pe-013	O62709	Peptide	Endothelin type B
Pe-013	P48302	Peptide	Endothelin type B

Pe-013	P28088	Peptide	Endothelin type B
Pe-013	P56497	Peptide	Endothelin type B
Pe-013	P24530	Peptide	Endothelin type B
Pe-014	O19037	Peptide	Melanocortin type 1
Pe-014	P55167	Peptide	Melanocortin type 1
Pe-014	P56447	Peptide	Melanocortin type 1
Pe-014	P56446	Peptide	Melanocortin type 1
Pe-014	P56445	Peptide	Melanocortin type 1
Pe-014	P56442	Peptide	Melanocortin type 1
Pe-014	P56448	Peptide	Melanocortin type 1
Pe-014	P47798	Peptide	Melanocortin type 1
Pe-014	P56444	Peptide	Melanocortin type 1
Pe-014	P56443	Peptide	Melanocortin type 1
Pe-015	Q95LF7	Peptide	Duffy antigen
Pe-015	Q95LG5	Peptide	Duffy antigen
Pe-015	Q9QUI6	Peptide	Duffy antigen
Pe-015	Q95LF5	Peptide	Duffy antigen
Pe-015	Q95LF4	Peptide	Duffy antigen
Pe-015	Q16570	Peptide	Duffy antigen
Pe-015	Q95LF2	Peptide	Duffy antigen
Pe-015	Q95LF9	Peptide	Duffy antigen
Pe-015	Q95LF3	Peptide	Duffy antigen
Pe-016	Q8HXX3	Peptide	Melanocortin type 4
Pe-016	P56450	Peptide	Melanocortin type 4
Pe-016	Q9GLJ8	Peptide	Melanocortin type 4
Pe-016	P70596	Peptide	Melanocortin type 4
Pe-016	P32245	Peptide	Melanocortin type 4
Pe-016	O97504	Peptide	Melanocortin type 4
Pe-016	Q0Z8I9	Peptide	Melanocortin type 4
Pe-016	B0V1P1	Peptide	Melanocortin type 4
Pe-017	P56481	Peptide	CCK 2
Pe-017	P30552	Peptide	CCK 2
Pe-017	P46627	Peptide	CCK 2
Pe-017	P30796	Peptide	CCK 2
Pe-017	P32239	Peptide	CCK 2
Pe-017	P30553	Peptide	CCK 2
Pe-017	P79266	Peptide	CCK 2
Pe-017	P70031	Peptide	CCK 3
Pe-018	Q9TV16	Peptide	C-X-C Chemokine type 6 (Bonzo)
Pe-018	Q9EQ16	Peptide	C-X-C Chemokine type 6 (Bonzo)
Pe-018	O19024	Peptide	C-X-C Chemokine type 6 (Bonzo)
Pe-018	Q9XT45	Peptide	C-X-C Chemokine type 6 (Bonzo)
Pe-018	Q9N0Z0	Peptide	C-X-C Chemokine type 6 (Bonzo)
Pe-018	O00574	Peptide	C-X-C Chemokine type 6 (Bonzo)
Pe-018	O18983	Peptide	C-X-C Chemokine type 6 (Bonzo)

Pe-018	Q9BDS6	Peptide	C-X-C Chemokine type 6 (Bonzo)
Pe-019	Q2YEG0	Peptide	Interleukin-8 type A
Pe-019	P55919	Peptide	Interleukin-8 type A
Pe-019	Q2YEG2	Peptide	Interleukin-8 type A
Pe-019	P55920	Peptide	Interleukin-8 type A
Pe-019	P21109	Peptide	Interleukin-8 type A
Pe-019	P25024	Peptide	Interleukin-8 type A
Pe-019	Q2YEF9	Peptide	Interleukin-8 type A
Pe-019	Q28003	Peptide	Interleukin-8 type B
Pe-020	Q6H2Y3	Peptide	Bombesin type 3
Pe-020	O97967	Peptide	Bombesin type 3
Pe-020	P35371	Peptide	Bombesin type 3
Pe-020	O54798	Peptide	Bombesin type 3
Pe-020	Q8K418	Peptide	Bombesin type 3
Pe-020	P32247	Peptide	Bombesin type 3
Pe-020	P47751	Peptide	Bombesin type 4
Pe-021	Q9WTV8	Peptide	Vasopressin type 1a
Pe-021	P48043	Peptide	Vasopressin type 1a
Pe-021	Q62463	Peptide	Vasopressin type 1a
Pe-021	P30560	Peptide	Vasopressin type 1a
Pe-021	Q9WTV9	Peptide	Vasopressin type 1a
Pe-021	P37288	Peptide	Vasopressin type 1a
Pe-021	Q90352	Peptide	Vasotocin
Pe-022	P30558	Peptide	Proteinase-activated type 1
Pe-022	P25116	Peptide	Proteinase-activated type 1
Pe-022	P56488	Peptide	Proteinase-activated type 1
Pe-022	P47749	Peptide	Proteinase-activated type 1
Pe-022	P26824	Peptide	Proteinase-activated type 1
Pe-022	A7YY44	Peptide	Proteinase-activated type 1
Pe-022	Q00991	Peptide	Proteinase-activated type 1
Pe-023	P21462	Peptide	Fmet-leu-phe
Pe-023	Q05394	Peptide	Fmet-leu-phe
Pe-023	P79235	Peptide	Fmet-leu-phe
Pe-023	P79176	Peptide	Fmet-leu-phe
Pe-023	P79189	Peptide	Fmet-leu-phe
Pe-023	P33766	Peptide	Fmet-leu-phe
Pe-023	P79241	Peptide	Fmet-leu-phe
Pe-024	Q28929	Peptide	Angiotensin type 2
Pe-024	P35374	Peptide	Angiotensin type 2
Pe-024	P50052	Peptide	Angiotensin type 2
Pe-024	P35351	Peptide	Angiotensin type 2
Pe-024	Q9Z0Z6	Peptide	Angiotensin type 2
Pe-025	Q2Y2P0	Peptide	C-C Chemokine type 1
Pe-025	P51675	Peptide	C-C Chemokine type 1
Pe-025	P51676	Peptide	C-C Chemokine type 1

Pe-025	P32246	Peptide	C-C Chemokine type 1
Pe-025	P56482	Peptide	C-C Chemokine type 1
Pe-026	Q6BD04	Peptide	Kiss receptor (GPR54)
Pe-026	Q91V45	Peptide	Kiss receptor (GPR54)
Pe-026	Q969F8	Peptide	Kiss receptor (GPR54)
Pe-026	Q924U1	Peptide	Kiss receptor (GPR54)
Pe-027	P35346	Peptide	Somatostatin type 5
Pe-027	O08858	Peptide	Somatostatin type 5
Pe-027	P30938	Peptide	Somatostatin type 5
Pe-028	Q695P6	Peptide	C5a anaphylatoxin C5L2
Pe-028	Q9P296	Peptide	C5a anaphylatoxin C5L2
Pe-028	Q8BW93	Peptide	C5a anaphylatoxin C5L2
Pe-029	P34974	Peptide	Adrenocorticotropic hormone
Pe-029	Q9TU77	Peptide	Adrenocorticotropic hormone
Pe-029	Q8HYN8	Peptide	Adrenocorticotropic hormone
Pe-030	P43142	Peptide	Adrenomedullin (G10D) 1
Pe-030	O15218	Peptide	Adrenomedullin (G10D) 1
Pe-030	P31392	Peptide	Adrenomedullin (G10D) 1
Pe-031	O88853	Peptide	Galanin type 3
Pe-031	O60755	Peptide	Galanin type 3
Pe-031	O88626	Peptide	Galanin type 3
Pe-032	Q9MZV8	Peptide	Melanocortin type 5
Pe-032	P56451	Peptide	Melanocortin type 5
Pe-032	P41983	Peptide	Melanocortin type 5
Pe-033	Q2KTE1	Peptide	C-X3-C Chemokine
Pe-033	A6QNL7	Peptide	C-X3-C Chemokine
Pe-033	P49238	Peptide	C-X3-C Chemokine
Pe-034	Q8VIH9	Peptide	Urotensin II
Pe-034	P49220	Peptide	Urotensin II
Pe-034	P49684	Peptide	Urotensin II
Pe-035	Q01727	Peptide	Melanocortin type 1
Pe-035	Q80SS9	Peptide	Melanocortin type 1
Pe-035	Q80SZ5	Peptide	Melanocortin type 1
Pe-036	Q99463	Peptide	Neuropeptide Y / peptide YY
Pe-036	Q61212	Peptide	Neuropeptide Y / peptide YY
Pe-036	P79217	Peptide	Neuropeptide Y / peptide YY
Pe-037	O88634	Peptide	Proteinase-activated type 4
Pe-037	Q920E0	Peptide	Proteinase-activated type 4
Pe-037	Q96RI0	Peptide	Proteinase-activated type 4
Pe-038	O00590	Peptide	C-C Chemokine type D6
Pe-038	O08707	Peptide	C-C Chemokine type D6
Pe-038	O09027	Peptide	C-C Chemokine type D6
Pe-039	O35457	Peptide	C-C Chemokine 102
Pe-039	Q9XSD7	Peptide	C-C Chemokine type 2-like
Pe-039	O00421	Peptide	C-C Chemokine type 2-like

Pe-040	O55040	Peptide	Neuromedin U
Pe-040	Q9JJI5	Peptide	Neuromedin U
Pe-040	Q9HB89	Peptide	Neuromedin U
Pe-041	P56484	Peptide	C-C Chemokine type 8
Pe-041	P51685	Peptide	C-C Chemokine type 8
Pe-041	O97665	Peptide	C-C Chemokine type 8
Pe-042	P0C7U4	Peptide	C5a anaphylatoxin
Pe-042	Q2WED0	Peptide	C5a anaphylatoxin
Pe-043	Q08520	Peptide	C-C Chemokine type 8
Pe-043	P32229	Peptide	C-C Chemokine type 8
Pe-044	P0C5I1	Peptide	GPR25
Pe-044	O00155	Peptide	GPR25
Pe-045	A4FUQ5	Peptide	Fmet-leu-phe
Pe-045	O88537	Peptide	Fmet-leu-phe
Pe-046	Q9R0M1	Peptide	XC Chemokine
Pe-046	P46094	Peptide	XC Chemokine
Pe-047	P46092	Peptide	C-C Chemokine type 10
Pe-047	Q9JL21	Peptide	C-C Chemokine type 10
Pe-048	Q8TDU9	Peptide	Relaxin-3 type 2
Pe-048	Q7TQP4	Peptide	Relaxin-3 type 2
Pe-049	O75388	Peptide	Fmet-leu-phe
Pe-049	Q8NGA4	Peptide	Fmet-leu-phe
Pe-050	Q8MJV2	Peptide	Somatostatin type 5
Pe-050	P48146	Peptide	Somatostatin type 5
Pe-051	Q86VZ1	Peptide	Proteinase-activated type 2
Pe-051	Q5ZI82	Peptide	Proteinase-activated type 2
Pe-052	Q75ZH0	Peptide	C-C Chemokine type 2-like
Pe-052	Q0II78	Peptide	C-C Chemokine type 2-like
Pe-053	Q6UNA4	Peptide	C5a anaphylatoxin
Pe-053	P0C7U5	Peptide	C5a anaphylatoxin
Pe-054	Q95NC7	Peptide	C-C Chemokine type 5
Pe-054	O97879	Peptide	C-C Chemokine type 5
Pe-054	P56493	Peptide	C-C Chemokine type 5
Pe-054	Q95NC4	Peptide	C-C Chemokine type 5
Pe-054	Q95NC1	Peptide	C-C Chemokine type 5
Pe-054	O08556	Peptide	C-C Chemokine type 5
Pe-054	Q2HJ17	Peptide	C-C Chemokine type 5
Pe-054	O62743	Peptide	C-C Chemokine type 5
Pe-054	Q9XT76	Peptide	C-C Chemokine type 5
Pe-054	Q95NC9	Peptide	C-C Chemokine type 5
Pe-054	Q5ECR9	Peptide	C-C Chemokine type 5
Pe-054	P68269	Peptide	C-C Chemokine type 5
Pe-054	P61815	Peptide	C-C Chemokine type 5
Pe-054	P51681	Peptide	C-C Chemokine type 5
Pe-054	Q9TV49	Peptide	C-C Chemokine type 5

Pe-054	P56440	Peptide	C-C Chemokine type 5
Pe-054	O97883	Peptide	C-C Chemokine type 5
Pe-054	Q95NC3	Peptide	C-C Chemokine type 5
Pe-054	P61814	Peptide	C-C Chemokine type 5
Pe-054	Q95NC8	Peptide	C-C Chemokine type 5
Pe-054	Q95ND2	Peptide	C-C Chemokine type 5
Pe-054	Q9TV45	Peptide	C-C Chemokine type 5
Pe-054	O97878	Peptide	C-C Chemokine type 5
Pe-054	P68270	Peptide	C-C Chemokine type 5
Pe-054	P61756	Peptide	C-C Chemokine type 5
Pe-054	O97882	Peptide	C-C Chemokine type 5
Pe-054	Q9TV48	Peptide	C-C Chemokine type 5
Pe-054	Q95NC6	Peptide	C-C Chemokine type 5
Pe-054	Q1ZY22	Peptide	C-C Chemokine type 5
Pe-054	P61757	Peptide	C-C Chemokine type 5
Pe-054	Q95ND1	Peptide	C-C Chemokine type 5
Pe-054	Q9TV43	Peptide	C-C Chemokine type 5
Pe-054	Q9BGN6	Peptide	C-C Chemokine type 5
Pe-054	O97880	Peptide	C-C Chemokine type 5
Pe-054	O97881	Peptide	C-C Chemokine type 5
Pe-054	Q8HZT9	Peptide	C-C Chemokine type 5
Pe-054	P51682	Peptide	C-C Chemokine type 5
Pe-054	Q9TV42	Peptide	C-C Chemokine type 5
Pe-054	O97962	Peptide	C-C Chemokine type 5
Pe-054	Q95NC0	Peptide	C-C Chemokine type 5
Pe-054	P61755	Peptide	C-C Chemokine type 5
Pe-054	P60574	Peptide	C-C Chemokine type 5
Pe-054	O97975	Peptide	C-C Chemokine type 5
Pe-054	Q95NE8	Peptide	C-C Chemokine type 5
Pe-054	P61813	Peptide	C-C Chemokine type 5
Pe-054	P56439	Peptide	C-C Chemokine type 5
Pe-054	Q9TV47	Peptide	C-C Chemokine type 5
Pe-054	Q95NC2	Peptide	C-C Chemokine type 5
Pe-054	Q95ND0	Peptide	C-C Chemokine type 5
Pe-054	Q6WN98	Peptide	C-C Chemokine type 5
Pe-054	Q95NC5	Peptide	C-C Chemokine type 5
Pe-055	P61072	Peptide	C-X-C Chemokine type 4
Pe-055	Q8HZU0	Peptide	C-X-C Chemokine type 4
Pe-055	P25930	Peptide	C-X-C Chemokine type 4
Pe-055	P70658	Peptide	C-X-C Chemokine type 4
Pe-055	P56491	Peptide	C-X-C Chemokine type 4
Pe-055	Q764M9	Peptide	C-X-C Chemokine type 4
Pe-055	O08565	Peptide	C-X-C Chemokine type 4
Pe-055	P79394	Peptide	C-X-C Chemokine type 4
Pe-055	Q9TSQ8	Peptide	C-X-C Chemokine type 4

Pe-055	Q28474	Peptide	C-X-C Chemokine type 4
Pe-055	Q8HZU1	Peptide	C-X-C Chemokine type 4
Pe-055	Q3LSL6	Peptide	C-X-C Chemokine type 4
Pe-055	P56498	Peptide	C-X-C Chemokine type 4
Pe-055	P61073	Peptide	C-X-C Chemokine type 4
Pe-055	Q7YS92	Peptide	C-X-C Chemokine type 4
Pe-055	O62747	Peptide	C-X-C Chemokine type 4
Pe-056	Q9WV26	Peptide	Angiotensin type 1
Pe-056	P29754	Peptide	Angiotensin type 1
Pe-056	P43240	Peptide	Angiotensin type 1
Pe-056	P25095	Peptide	Angiotensin type 1
Pe-056	P30555	Peptide	Angiotensin type 1
Pe-056	Q9GLN9	Peptide	Angiotensin type 1
Pe-056	P34976	Peptide	Angiotensin type 1
Pe-056	P29089	Peptide	Angiotensin type 1
Pe-056	O77590	Peptide	Angiotensin type 1
Pe-056	P29755	Peptide	Angiotensin type 1
Pe-056	P33396	Peptide	Angiotensin type 1
Pe-056	P25104	Peptide	Angiotensin type 1
Pe-056	O35210	Peptide	Angiotensin type 1
Pe-056	P30556	Peptide	Angiotensin type 1
Pe-056	P79785	Peptide	Angiotensin type 1
Pe-057	Q95247	Peptide	Opioid type M
Pe-057	P79350	Peptide	Opioid type M
Pe-057	Q9MYW9	Peptide	Opioid type M
Pe-057	P33535	Peptide	Opioid type M
Pe-057	Q5IS84	Peptide	Opioid type M
Pe-057	Q95M54	Peptide	Opioid type M
Pe-057	Q5IS39	Peptide	Opioid type M
Pe-057	P35372	Peptide	Opioid type M
Pe-057	P42866	Peptide	Opioid type M
Pe-058	Q1RMU8	Peptide	Neuropeptide Y type 1
Pe-058	P25929	Peptide	Neuropeptide Y type 1
Pe-058	Q04573	Peptide	Neuropeptide Y type 1
Pe-058	P34992	Peptide	Neuropeptide Y type 1
Pe-058	O02835	Peptide	Neuropeptide Y type 1
Pe-058	Q9WVD0	Peptide	Neuropeptide Y type 1
Pe-058	O02813	Peptide	Neuropeptide Y type 1
Pe-058	P21555	Peptide	Neuropeptide Y type 1
Pe-059	Q8K458	Peptide	Prokineticin receptors
Pe-059	Q8R416	Peptide	Prokineticin receptors
Pe-059	Q8TCW9	Peptide	Prokineticin receptors
Pe-059	Q8SPN1	Peptide	Prokineticin receptors
Pe-059	Q8R415	Peptide	Prokineticin receptors
Pe-059	Q9JKL1	Peptide	Prokineticin receptors

Pe-059	Q8SPN2	Peptide	Prokineticin receptors
Pe-059	Q8NFJ6	Peptide	Prokineticin receptors
Pe-060	Q5IS62	Peptide	Neuropeptide Y type 2
Pe-060	P97295	Peptide	Neuropeptide Y type 2
Pe-060	Q9DDN6	Peptide	Neuropeptide Y type 2
Pe-060	P49146	Peptide	Neuropeptide Y type 2
Pe-060	Q9Z2D5	Peptide	Neuropeptide Y type 2
Pe-060	O02836	Peptide	Neuropeptide Y type 2
Pe-060	Q9GK74	Peptide	Neuropeptide Y type 2
Pe-060	P79113	Peptide	Neuropeptide Y type 2
Pe-061	P16610	Peptide	Substance K (NK2)
Pe-061	P05363	Peptide	Substance K (NK2)
Pe-061	Q64077	Peptide	Substance K (NK2)
Pe-061	Q5DUB2	Peptide	Substance K (NK2)
Pe-061	P21452	Peptide	Substance K (NK2)
Pe-061	P51144	Peptide	Substance K (NK2)
Pe-061	P30549	Peptide	Substance K (NK2)
Pe-061	P79218	Peptide	Substance K (NK2)
Pe-062	Q0GBZ5	Peptide	Orexin type 1
Pe-062	P58307	Peptide	Orexin type 1
Pe-062	O43613	Peptide	Orexin type 1
Pe-062	P56718	Peptide	Orexin type 1
Pe-062	O43614	Peptide	Orexin type 2
Pe-062	P58308	Peptide	Orexin type 2
Pe-062	P56719	Peptide	Orexin type 2
Pe-062	Q9TUP7	Peptide	Orexin type 2
Pe-063	Q61614	Peptide	Endothelin type A
Pe-063	P26684	Peptide	Endothelin type A
Pe-063	Q95L55	Peptide	Endothelin type A
Pe-063	Q5KSU9	Peptide	Endothelin type A
Pe-063	P21450	Peptide	Endothelin type A
Pe-063	Q29010	Peptide	Endothelin type A
Pe-063	P25101	Peptide	Endothelin type A
Pe-064	Q5DUB3	Peptide	Substance P (NK1)
Pe-064	Q98982	Peptide	Substance P (NK1)
Pe-064	P25103	Peptide	Substance P (NK1)
Pe-064	P14600	Peptide	Substance P (NK1)
Pe-064	Q5DUB1	Peptide	Substance P (NK1)
Pe-064	P30548	Peptide	Substance P (NK1)
Pe-064	P30547	Peptide	Substance P (NK1)
Pe-065	Q0VDU3	Peptide	APJ like Non Vertebrate type 1
Pe-065	Q9BG77	Peptide	GPR15
Pe-065	Q9BDS7	Peptide	GPR15
Pe-065	P56412	Peptide	GPR15
Pe-065	P49685	Peptide	GPR15

Pe-065	O18982	Peptide	GPR15
Pe-065	O97663	Peptide	GPR15
Pe-066	O88680	Peptide	C3a anaphylatoxin
Pe-066	Q16581	Peptide	C3a anaphylatoxin
Pe-066	O55197	Peptide	C3a anaphylatoxin
Pe-066	Q6TAC8	Peptide	C3a anaphylatoxin
Pe-066	Q5REI5	Peptide	C3a anaphylatoxin
Pe-066	O09047	Peptide	C3a anaphylatoxin
Pe-067	O88410	Peptide	C-X-C Chemokine type 3
Pe-067	Q867B2	Peptide	C-X-C Chemokine type 3
Pe-067	P49682	Peptide	C-X-C Chemokine type 3
Pe-067	Q9JII9	Peptide	C-X-C Chemokine type 3
Pe-067	Q5MD61	Peptide	C-X-C Chemokine type 3
Pe-067	Q5KSK8	Peptide	C-X-C Chemokine type 3
Pe-068	O08786	Peptide	CCK 1
Pe-068	Q63931	Peptide	CCK 1
Pe-068	P30551	Peptide	CCK 1
Pe-068	O97772	Peptide	CCK 1
Pe-068	P32238	Peptide	CCK 1
Pe-068	Q5D0K2	Peptide	CCK 1
Pe-069	Q00788	Peptide	Vasopressin type 2
Pe-069	O77808	Peptide	Vasopressin type 2
Pe-069	P48044	Peptide	Vasopressin type 2
Pe-069	P32307	Peptide	Vasopressin type 2
Pe-069	P30518	Peptide	Vasopressin type 2
Pe-069	O88721	Peptide	Vasopressin type 2
Pe-070	Q28642	Peptide	Bradykinin type B2
Pe-070	O70526	Peptide	Bradykinin type B2
Pe-070	P25023	Peptide	Bradykinin type B2
Pe-070	Q9GLX8	Peptide	Bradykinin type B2
Pe-070	P32299	Peptide	Bradykinin type B2
Pe-070	P30411	Peptide	Bradykinin type B2
Pe-071	P30874	Peptide	Somatostatin type 2
Pe-071	P34993	Peptide	Somatostatin type 2
Pe-071	P30875	Peptide	Somatostatin type 2
Pe-071	P34994	Peptide	Somatostatin type 2
Pe-071	P30680	Peptide	Somatostatin type 2
Pe-071	Q49LX6	Peptide	Somatostatin type 2
Pe-072	P35370	Peptide	Opioid type X
Pe-072	P79292	Peptide	Opioid type X
Pe-072	P35377	Peptide	Opioid type X
Pe-072	P41146	Peptide	Opioid type X
Pe-072	P47748	Peptide	Opioid type X
Pe-073	B1PHQ8	Peptide	Fmet-leu-phe
Pe-073	B9VR26	Peptide	Fmet-leu-phe

Pe-073	Q99788	Peptide	Fmet-leu-phe
Pe-073	P97468	Peptide	Fmet-leu-phe
Pe-073	O35786	Peptide	Fmet-leu-phe
Pe-074	Q2KIP6	Peptide	Opioid type K
Pe-074	P41144	Peptide	Opioid type K
Pe-074	P34975	Peptide	Opioid type K
Pe-074	P41145	Peptide	Opioid type K
Pe-074	P33534	Peptide	Opioid type K
Pe-075	P25089	Peptide	Fmet-leu-phe
Pe-075	P79191	Peptide	Fmet-leu-phe
Pe-075	P79243	Peptide	Fmet-leu-phe
Pe-075	P79178	Peptide	Fmet-leu-phe
Pe-075	P79237	Peptide	Fmet-leu-phe
Pe-076	P79177	Peptide	Fmet-leu-phe
Pe-076	P79236	Peptide	Fmet-leu-phe
Pe-076	P79190	Peptide	Fmet-leu-phe
Pe-076	P25090	Peptide	Fmet-leu-phe
Pe-076	P79242	Peptide	Fmet-leu-phe
Pe-077	Q15761	Peptide	Neuropeptide Y type 5
Pe-077	O62729	Peptide	Neuropeptide Y type 5
Pe-077	O70342	Peptide	Neuropeptide Y type 5
Pe-077	O97969	Peptide	Neuropeptide Y type 5
Pe-077	Q63634	Peptide	Neuropeptide Y type 5
Pe-078	Q8MJ89	Peptide	Melanin-concentrating hormone receptors
Pe-078	P97639	Peptide	Melanin-concentrating hormone receptors
Pe-078	Q8JZL2	Peptide	Melanin-concentrating hormone receptors
Pe-078	Q5IJ49	Peptide	Melanin-concentrating hormone receptors
Pe-078	Q99705	Peptide	Melanin-concentrating hormone receptors
Pe-079	P46090	Peptide	Fmet-leu-phe
Pe-079	O97664	Peptide	Fmet-leu-phe
Pe-079	P46091	Peptide	Fmet-leu-phe
Pe-079	Q8K087	Peptide	Fmet-leu-phe
Pe-079	Q95LH1	Peptide	Fmet-leu-phe
Pe-080	Q8BZ39	Peptide	Neuromedin U
Pe-080	Q9ESQ4	Peptide	Neuromedin U
Pe-080	Q58CW4	Peptide	Neuromedin U
Pe-080	Q9GZQ4	Peptide	Neuromedin U
Pe-081	Q8BZP8	Peptide	Vasotocin
Pe-081	Q6W5P4	Peptide	Vasotocin
Pe-081	P0C0L6	Peptide	Vasotocin
Pe-081	Q56H79	Peptide	Vasotocin
Pe-082	O60883	Peptide	GPR37 / endothelin B-like
Pe-082	Q9QYC5	Peptide	GPR37 / endothelin B-like
Pe-082	Q17QD8	Peptide	GPR37 / endothelin B-like
Pe-082	Q99JG2	Peptide	GPR37 / endothelin B-like

Pe-083	Q63645	Peptide	Proteinase-activated type 2
Pe-083	Q2HJA4	Peptide	Proteinase-activated type 2
Pe-083	P55086	Peptide	Proteinase-activated type 2
Pe-083	P55085	Peptide	Proteinase-activated type 2
Pe-084	O18793	Peptide	C-C Chemokine type 2
Pe-084	P41597	Peptide	C-C Chemokine type 2
Pe-084	P51683	Peptide	C-C Chemokine type 2
Pe-084	O55193	Peptide	C-C Chemokine type 2
Pe-085	P30873	Peptide	Somatostatin type 1
Pe-085	P30872	Peptide	Somatostatin type 1
Pe-085	P28646	Peptide	Somatostatin type 1
Pe-085	Q49LX5	Peptide	Somatostatin type 1
Pe-086	O97666	Peptide	APJ like type 1
Pe-086	Q9JHG3	Peptide	APJ like type 1
Pe-086	Q9WV08	Peptide	APJ like type 1
Pe-086	P35414	Peptide	APJ like type 1
Pe-087	Q9BDS8	Peptide	C-C Chemokine type 3
Pe-087	P56483	Peptide	C-C Chemokine type 3
Pe-087	P51677	Peptide	C-C Chemokine type 3
Pe-087	P56492	Peptide	C-C Chemokine type 3
Pe-088	Q4EW11	Peptide	Prolactin-releasing peptide (GPR10)
Pe-088	P49683	Peptide	Prolactin-releasing peptide (GPR10)
Pe-088	Q6VMN6	Peptide	Prolactin-releasing peptide (GPR10)
Pe-088	Q64121	Peptide	Prolactin-releasing peptide (GPR10)
Pe-089	P52500	Peptide	Gastrin-releasing peptide receptor
Pe-089	P21729	Peptide	Gastrin-releasing peptide receptor
Pe-089	P30550	Peptide	Gastrin-releasing peptide receptor
Pe-090	P70585	Peptide	Somatostatin type 5
Pe-090	Q61121	Peptide	Somatostatin type 5
Pe-090	Q15760	Peptide	Somatostatin type 5
Pe-091	Q924I3	Peptide	C-C Chemokine type 11
Pe-091	P35350	Peptide	C-C Chemokine type 11
Pe-091	Q9NPB9	Peptide	C-C Chemokine type 11
Pe-092	Q49SQ3	Peptide	Chemokine receptor-like 1
Pe-092	Q49SQ1	Peptide	Chemokine receptor-like 1
Pe-092	Q49SQ2	Peptide	Chemokine receptor-like 1
Pe-093	Q29154	Peptide	Melanocortin type 1
Pe-093	Q6A155	Peptide	Melanocortin type 1
Pe-093	O77616	Peptide	Melanocortin type 1
Pe-094	O88854	Peptide	Galanin type 2
Pe-094	O43603	Peptide	Galanin type 2
Pe-094	O08726	Peptide	Galanin type 2
Pe-095	Q9Z2J6	Peptide	Fmet-leu-phe
Pe-095	Q9Y5Y4	Peptide	Fmet-leu-phe
Pe-095	Q6XKD3	Peptide	Fmet-leu-phe

Pe-096	O08878	Peptide	Chemokine receptor-like 2
Pe-096	Q8BMP4	Peptide	Chemokine receptor-like 2
Pe-096	Q99527	Peptide	Chemokine receptor-like 2
Pe-097	P33533	Peptide	Opioid type D
Pe-097	P41143	Peptide	Opioid type D
Pe-097	P32300	Peptide	Opioid type D
Pe-098	Q4VA82	Peptide	APJ like Non Vertebrate type 1
Pe-098	Q2TAD5	Peptide	APJ like Non Vertebrate type 1
Pe-098	P79960	Peptide	APJ like Non Vertebrate type 1
Pe-099	Q9WU02	Peptide	Vasopressin type 1b
Pe-099	P47901	Peptide	Vasopressin type 1b
Pe-099	P48974	Peptide	Vasopressin type 1b
Pe-100	Q9QY42	Peptide	GPR37 / endothelin B-like
Pe-100	O15354	Peptide	GPR37 / endothelin B-like
Pe-100	Q9QYC6	Peptide	GPR37 / endothelin B-like
Pe-101	P51679	Peptide	C-C Chemokine type 4
Pe-101	Q8MJW8	Peptide	C-C Chemokine type 4
Pe-101	P51680	Peptide	C-C Chemokine type 4
Pe-102	Q63447	Peptide	Neuropeptide Y type 4
Pe-102	P50391	Peptide	Neuropeptide Y type 4
Pe-102	Q61041	Peptide	Neuropeptide Y type 4
Pe-103	P49660	Peptide	Somatostatin type 4
Pe-103	P30937	Peptide	Somatostatin type 4
Pe-103	P31391	Peptide	Somatostatin type 4
Pe-104	P32248	Peptide	C-C Chemokine type 7
Pe-104	P47774	Peptide	C-C Chemokine type 7
Pe-104	Q5MD62	Peptide	C-C Chemokine type 7
Pe-105	Q2T9S0	Peptide	Melanocortin type 1
Pe-105	Q60HC2	Peptide	Melanocortin type 1
Pe-105	Q13509	Peptide	Melanocortin type 1
Pe-106	Q1WLP9	Peptide	C-C Chemokine type 9
Pe-106	P51686	Peptide	C-C Chemokine type 9
Pe-106	Q9WUT7	Peptide	C-C Chemokine type 9
Pe-107	Q04683	Peptide	C-X-C Chemokine type 5
Pe-107	P32302	Peptide	C-X-C Chemokine type 5
Pe-107	P34997	Peptide	C-X-C Chemokine type 5
Pe-108	Q96P65	Peptide	Orexigenic neuropeptide QRFP
Pe-108	P83858	Peptide	Orexigenic neuropeptide QRFP
Pe-108	P83861	Peptide	Orexigenic neuropeptide QRFP
Pe-109	O95665	Peptide	Neurotensin type 2
Pe-109	P70310	Peptide	Neurotensin type 2
Pe-109	Q63384	Peptide	Neurotensin type 2
Pe-110	Q924H0	Peptide	Neuropeptide FF type 2
Pe-110	Q9Y5X5	Peptide	Neuropeptide FF type 2
Pe-110	Q9EQD2	Peptide	Neuropeptide FF type 2

Pe-111	P47211	Peptide	Galanin type 1
Pe-111	P56479	Peptide	Galanin type 1
Pe-111	Q62805	Peptide	Galanin type 1
Pe-112	Q7ZXJ7	Peptide	C-X-C Chemokine type 4
Pe-112	Q9YGC3	Peptide	C-X-C Chemokine type 4
Pe-112	Q07FZ4	Peptide	C-X-C Chemokine type 4
Pe-113	P30731	Peptide	GPR83 like
Pe-113	Q9TTQ9	Peptide	GPR83 like
Pe-113	Q9NYM4	Peptide	GPR83 like
Pe-114	Q8CIM5	Peptide	Kiss receptor (GPR54)
Pe-114	Q9NQS5	Peptide	Kiss receptor (GPR54)
Pe-114	Q2KI97	Peptide	Kiss receptor (GPR54)
Pe-115	P32745	Peptide	Somatostatin type 3
Pe-115	P30935	Peptide	Somatostatin type 3
Pe-115	P30936	Peptide	Somatostatin type 3
Pe-116	Q8MJ88	Peptide	Melanin-concentrating hormone receptors
Pe-116	Q969V1	Peptide	Melanin-concentrating hormone receptors
Pe-116	Q8SQ54	Peptide	Melanin-concentrating hormone receptors
Pe-117	P28336	Peptide	Neuromedin B receptor
Pe-117	P24053	Peptide	Neuromedin B receptor
Pe-117	O54799	Peptide	Neuromedin B receptor
Pe-118	P20789	Peptide	Neurotensin type 1
Pe-118	O88319	Peptide	Neurotensin type 1
Pe-118	P30989	Peptide	Neurotensin type 1
Pe-119	Q863H8	Peptide	Duffy antigen
Pe-119	Q9GLX0	Peptide	Duffy antigen
Pe-120	P70612	Peptide	Interleukin-8 type A
Pe-120	Q810W6	Peptide	Interleukin-8 type A
Pe-121	Q9NSD7	Peptide	Relaxin-3 type 1
Pe-121	Q8BGE9	Peptide	Relaxin-3 type 1
Pe-122	Q6NV75	Peptide	Melanin-concentrating hormone receptors
Pe-122	Q8K0Z9	Peptide	Melanin-concentrating hormone receptors
Pe-123	P32303	Peptide	Angiotensin type 1
Pe-123	P35373	Peptide	Angiotensin type 1
Pe-124	Q8BXS7	Peptide	Melanin-concentrating hormone receptors
Pe-124	Q8TDT2	Peptide	Melanin-concentrating hormone receptors
Pe-125	Q29J90	Peptide	Melanin-concentrating hormone receptors
Pe-125	Q9W534	Peptide	Melanin-concentrating hormone receptors
Pe-126	Q58D85	Peptide	Proteinase-activated type 3
Pe-126	O00254	Peptide	Proteinase-activated type 3
Pe-127	P30993	Peptide	C5a anaphylatoxin
Pe-127	P97520	Peptide	C5a anaphylatoxin
Pe-128	B3G515	Peptide	Chemokine receptor-like 2
Pe-128	B0F9W3	Peptide	Chemokine receptor-like 2
Pe-129	P32244	Peptide	Melanocortin type 3

Pe-129	P33033	Peptide	Melanocortin type 3
Pe-130	P51684	Peptide	C-C Chemokine type 6
Pe-130	O54689	Peptide	C-C Chemokine type 6
Pe-131	Q71MR7	Peptide	Fmet-leu-phe
Pe-131	Q3SXG2	Peptide	Fmet-leu-phe
Pe-132	Q16538	Peptide	Melanin-concentrating hormone receptors
Pe-132	Q3UN16	Peptide	Melanin-concentrating hormone receptors
Pe-133	P33032	Peptide	Melanocortin type 5
Pe-133	Q9TT23	Peptide	Melanocortin type 5
Pe-134	O88416	Peptide	Chemokine receptor-like 1
Pe-134	Q49SP8	Peptide	Chemokine receptor-like 1
Pe-135	Q8HYC3	Peptide	Urotensin II
Pe-135	Q9UKP6	Peptide	Urotensin II
Pe-136	P41149	Peptide	Melanocortin type 5
Pe-136	P35345	Peptide	Melanocortin type 5
Pe-137	P35411	Peptide	C-X3-C Chemokine
Pe-137	Q9Z0D9	Peptide	C-X3-C Chemokine
Pe-138	A0T2N3	Peptide	APJ like 3
Pe-138	Q7SZP9	Peptide	APJ like 3
Pe-139	P97583	Peptide	Bradykinin type B1
Pe-139	Q61125	Peptide	Bradykinin type B1
Pe-140	Q96P67	Peptide	Angiotensin type 1
Pe-140	Q8BZR0	Peptide	Angiotensin type 1
Pe-141	O08790	Peptide	Fmet-leu-phe
Pe-141	O88536	Peptide	Fmet-leu-phe
Pe-142	Q864F8	Peptide	Melanocortin type 1
Pe-142	Q864F7	Peptide	Melanocortin type 1
Pe-143	Q9EP86	Peptide	Neuropeptide FF type 1
Pe-143	Q9GZQ6	Peptide	Neuropeptide FF type 1
Pe-144	P35343	Peptide	Interleukin-8 type B
Pe-144	P35407	Peptide	Interleukin-8 type B
Pe-145	O08675	Peptide	Proteinase-activated type 3
Pe-145	Q920E1	Peptide	Proteinase-activated type 3
Pl-001	P21556	Platelet activating factor	Platelet activating factor
Pl-001	Q9TTY5	Platelet activating factor	Platelet activating factor
Pl-001	Q62035	Platelet activating factor	Platelet activating factor
Pl-001	Q9XSD4	Platelet activating factor	Platelet activating factor
Pl-001	P25105	Platelet activating factor	Platelet activating factor
Pl-001	Q9GK76	Platelet activating factor	Platelet activating factor
Pl-001	P46002	Platelet activating factor	Platelet activating factor
Pr-001	P35375	Prostanoid	Prostaglandin E2 subtype EP1
Pr-001	P34995	Prostanoid	Prostaglandin E2 subtype EP1
Pr-001	Q9BGL8	Prostanoid	Prostaglandin E2 subtype EP1
Pr-001	P70597	Prostanoid	Prostaglandin E2 subtype EP1
Pr-002	Q95125	Prostanoid	Thromboxane

Pr-002	P21731	Prostanoid	Thromboxane
Pr-002	P56486	Prostanoid	Thromboxane
Pr-003	A5D7K8	Prostanoid	Prostaglandin E2/D2 subtype EP2
Pr-003	Q13258	Prostanoid	Prostaglandin E2/D2 subtype EP2
Pr-004	P43114	Prostanoid	Prostaglandin E2 subtype EP4
Pr-004	P32240	Prostanoid	Prostaglandin E2 subtype EP4
Pr-004	Q95KZ0	Prostanoid	Prostaglandin E2 subtype EP4
Pr-004	Q8MJ08	Prostanoid	Prostaglandin E2 subtype EP4
Pr-004	P35408	Prostanoid	Prostaglandin E2 subtype EP4
Pr-004	Q28691	Prostanoid	Prostaglandin E2 subtype EP4
Pr-005	P43115	Prostanoid	Prostaglandin E2 subtype EP3
Pr-005	P50131	Prostanoid	Prostaglandin E2 subtype EP3
Pr-005	P46069	Prostanoid	Prostaglandin E2 subtype EP3
Pr-005	P34979	Prostanoid	Prostaglandin E2 subtype EP3
Pr-005	P34980	Prostanoid	Prostaglandin E2 subtype EP3
Pr-005	P30557	Prostanoid	Prostaglandin E2 subtype EP3
Pr-006	P43088	Prostanoid	Prostaglandin F2-alpha
Pr-006	P37289	Prostanoid	Prostaglandin F2-alpha
Pr-006	P43117	Prostanoid	Prostaglandin F2-alpha
Pr-006	P43118	Prostanoid	Prostaglandin F2-alpha
Pr-006	Q28905	Prostanoid	Prostaglandin F2-alpha
Pr-007	P79393	Prostanoid	Prostacyclin
Pr-007	P43119	Prostanoid	Prostacyclin
Pr-007	P43252	Prostanoid	Prostacyclin
Pr-007	P43253	Prostanoid	Prostacyclin
Pr-008	Q62928	Prostanoid	Prostaglandin E2/D2 subtype EP2
Pr-008	Q62053	Prostanoid	Prostaglandin E2/D2 subtype EP2
Pr-008	P43116	Prostanoid	Prostaglandin E2/D2 subtype EP2
Pr-008	Q9XT82	Prostanoid	Prostaglandin E2/D2 subtype EP2
Pr-009	O35932	Prostanoid	Prostaglandin E2/D2 subtype EP2
Pr-009	Q9R261	Prostanoid	Prostaglandin E2/D2 subtype EP2
Pr-009	P70263	Prostanoid	Prostaglandin E2/D2 subtype EP2
Pr-010	P34978	Prostanoid	Thromboxane
Pr-010	P30987	Prostanoid	Thromboxane
Rh-001	O42431	(Rhod)opsin	Vertebrate blue/green opsin
Rh-001	O42330	(Rhod)opsin	Vertebrate blue/green opsin
Rh-001	O42327	(Rhod)opsin	Vertebrate blue/green opsin
Rh-001	O42307	(Rhod)opsin	Vertebrate blue/green opsin
Rh-001	O42301	(Rhod)opsin	Vertebrate blue/green opsin
Rh-001	O42328	(Rhod)opsin	Vertebrate blue/green opsin
Rh-001	Q90373	(Rhod)opsin	Vertebrate blue/green opsin
Rh-001	O42294	(Rhod)opsin	Vertebrate blue/green opsin
Rh-001	O42451	(Rhod)opsin	Vertebrate blue/green opsin
Rh-001	O42300	(Rhod)opsin	Vertebrate blue/green opsin
Rh-001	O42427	(Rhod)opsin	Vertebrate blue/green opsin

Rh-001	O42452	(Rhod)opsin	Vertebrate blue/green opsin
Rh-002	P51472	(Rhod)opsin	Vertebrate blue/green opsin
Rh-002	Q9W6A8	(Rhod)opsin	Vertebrate blue/green opsin
Rh-002	P32310	(Rhod)opsin	Vertebrate blue/green opsin
Rh-002	P28682	(Rhod)opsin	Vertebrate blue/green opsin
Rh-002	P87365	(Rhod)opsin	Vertebrate blue/green opsin
Rh-003	O96107	(Rhod)opsin	Rhodopsin Arthropod short wavelength 1
Rh-003	P91657	(Rhod)opsin	Rhodopsin Arthropod short wavelength 1
Rh-003	Q26495	(Rhod)opsin	Rhodopsin Arthropod short wavelength 1
Rh-003	P90680	(Rhod)opsin	Rhodopsin Arthropod short wavelength 1
Rh-004	P04000	(Rhod)opsin	Vertebrate red opsin
Rh-004	P34989	(Rhod)opsin	Vertebrate red opsin
Rh-004	Q9W6A7	(Rhod)opsin	Vertebrate red opsin
Rh-004	P87367	(Rhod)opsin	Vertebrate red opsin
Rh-004	O35478	(Rhod)opsin	Vertebrate red opsin
Rh-004	Q9BGI7	(Rhod)opsin	Vertebrate red opsin
Rh-004	P22330	(Rhod)opsin	Vertebrate red opsin
Rh-004	P41592	(Rhod)opsin	Vertebrate red opsin
Rh-004	Q95170	(Rhod)opsin	Vertebrate red opsin
Rh-004	Q8AYN0	(Rhod)opsin	Vertebrate red opsin
Rh-004	P22331	(Rhod)opsin	Vertebrate red opsin
Rh-004	O18912	(Rhod)opsin	Vertebrate red opsin
Rh-004	P22329	(Rhod)opsin	Vertebrate red opsin
Rh-004	O18911	(Rhod)opsin	Vertebrate red opsin
Rh-004	O18910	(Rhod)opsin	Vertebrate red opsin
Rh-004	O18913	(Rhod)opsin	Vertebrate red opsin
Rh-004	O12948	(Rhod)opsin	Vertebrate red opsin
Rh-004	P04001	(Rhod)opsin	Vertebrate red opsin
Rh-004	Q9R024	(Rhod)opsin	Vertebrate red opsin
Rh-004	O18914	(Rhod)opsin	Vertebrate red opsin
Rh-004	O35599	(Rhod)opsin	Vertebrate red opsin
Rh-004	O35476	(Rhod)opsin	Vertebrate red opsin
Rh-004	P22332	(Rhod)opsin	Vertebrate red opsin
Rh-004	P32313	(Rhod)opsin	Vertebrate red opsin
Rh-004	P35358	(Rhod)opsin	Vertebrate red opsin
Rh-005	Q4R1I4	(Rhod)opsin	Cephalochordata melanopsin
Rh-005	Q2KNE5	(Rhod)opsin	Vertebrate melanopsin
Rh-005	Q9UHM6	(Rhod)opsin	Vertebrate melanopsin
Rh-005	Q5YKK9	(Rhod)opsin	Vertebrate melanopsin
Rh-005	Q8R456	(Rhod)opsin	Vertebrate melanopsin
Rh-005	Q9QXZ9	(Rhod)opsin	Vertebrate melanopsin
Rh-005	Q5XXP2	(Rhod)opsin	Vertebrate melanopsin
Rh-006	P04950	(Rhod)opsin	Rhodopsin Arthropod short wavelength 1
Rh-006	O61303	(Rhod)opsin	Rhodopsin Arthropod short wavelength 1
Rh-006	P08255	(Rhod)opsin	Rhodopsin Arthropod short wavelength 1

Rh-006	P29404	(Rhod)opsin	Rhodopsin Arthropod short wavelength 1
Rh-006	P17646	(Rhod)opsin	Rhodopsin Arthropod short wavelength 1
Rh-006	P28680	(Rhod)opsin	Rhodopsin Arthropod short wavelength 1
Rh-006	O02465	(Rhod)opsin	Rhodopsin Arthropod short wavelength 1
Rh-007	Q9Z2B3	(Rhod)opsin	Vertebrate red opsin
Rh-007	P47804	(Rhod)opsin	Vertebrate red opsin
Rh-007	P47803	(Rhod)opsin	Vertebrate red opsin
Rh-008	Q7T3Q7	(Rhod)opsin	Vertebrate ancient (long) opsin
Rh-008	O13018	(Rhod)opsin	Vertebrate ancient (long) opsin
Rh-008	O42490	(Rhod)opsin	Vertebrate P-opsin
Rh-009	P56515	(Rhod)opsin	Vertebrate blue/green opsin
Rh-009	O93441	(Rhod)opsin	Vertebrate blue/green opsin
Rh-009	Q68J47	(Rhod)opsin	Vertebrate blue/green opsin
Rh-009	P28681	(Rhod)opsin	Vertebrate blue/green opsin
Rh-009	Q9YGZ4	(Rhod)opsin	Vertebrate blue/green opsin
Rh-009	P79809	(Rhod)opsin	Vertebrate blue/green opsin
Rh-009	P52202	(Rhod)opsin	Vertebrate blue/green opsin
Rh-009	Q9YH01	(Rhod)opsin	Vertebrate blue/green opsin
Rh-009	P79902	(Rhod)opsin	Vertebrate blue/green opsin
Rh-009	P79911	(Rhod)opsin	Vertebrate blue/green opsin
Rh-009	Q9YH02	(Rhod)opsin	Vertebrate blue/green opsin
Rh-009	Q9YH05	(Rhod)opsin	Vertebrate blue/green opsin
Rh-009	P32309	(Rhod)opsin	Vertebrate blue/green opsin
Rh-009	Q9YGZ0	(Rhod)opsin	Vertebrate blue/green opsin
Rh-009	P02699	(Rhod)opsin	Vertebrate blue/green opsin
Rh-009	P79808	(Rhod)opsin	Vertebrate blue/green opsin
Rh-009	P79848	(Rhod)opsin	Vertebrate blue/green opsin
Rh-009	Q6W3E1	(Rhod)opsin	Vertebrate blue/green opsin
Rh-009	P51488	(Rhod)opsin	Vertebrate blue/green opsin
Rh-009	P02700	(Rhod)opsin	Vertebrate blue/green opsin
Rh-009	Q9YGZ7	(Rhod)opsin	Vertebrate blue/green opsin
Rh-009	O93459	(Rhod)opsin	Vertebrate blue/green opsin
Rh-009	P15409	(Rhod)opsin	Vertebrate blue/green opsin
Rh-009	P79812	(Rhod)opsin	Vertebrate blue/green opsin
Rh-009	O62793	(Rhod)opsin	Vertebrate blue/green opsin
Rh-009	P56516	(Rhod)opsin	Vertebrate blue/green opsin
Rh-009	P32308	(Rhod)opsin	Vertebrate blue/green opsin
Rh-009	P79756	(Rhod)opsin	Vertebrate blue/green opsin
Rh-009	Q95KU1	(Rhod)opsin	Vertebrate blue/green opsin
Rh-009	Q9YGZ2	(Rhod)opsin	Vertebrate blue/green opsin
Rh-009	Q98980	(Rhod)opsin	Vertebrate blue/green opsin
Rh-009	O18766	(Rhod)opsin	Vertebrate blue/green opsin
Rh-009	P41591	(Rhod)opsin	Vertebrate blue/green opsin
Rh-009	P35359	(Rhod)opsin	Vertebrate blue/green opsin
Rh-009	Q9YGZ8	(Rhod)opsin	Vertebrate blue/green opsin

Rh-009	P22328	(Rhod)opsin	Vertebrate blue/green opsin
Rh-009	O42268	(Rhod)opsin	Vertebrate blue/green opsin
Rh-009	P51470	(Rhod)opsin	Vertebrate blue/green opsin
Rh-009	O62798	(Rhod)opsin	Vertebrate blue/green opsin
Rh-009	Q8HY69	(Rhod)opsin	Vertebrate blue/green opsin
Rh-009	Q9YGZ6	(Rhod)opsin	Vertebrate blue/green opsin
Rh-009	Q9YGZ9	(Rhod)opsin	Vertebrate blue/green opsin
Rh-009	P87369	(Rhod)opsin	Vertebrate blue/green opsin
Rh-009	P31355	(Rhod)opsin	Vertebrate blue/green opsin
Rh-009	O42466	(Rhod)opsin	Vertebrate blue/green opsin
Rh-009	O62795	(Rhod)opsin	Vertebrate blue/green opsin
Rh-009	Q9YH03	(Rhod)opsin	Vertebrate blue/green opsin
Rh-009	O42604	(Rhod)opsin	Vertebrate blue/green opsin
Rh-009	P35403	(Rhod)opsin	Vertebrate blue/green opsin
Rh-009	P29403	(Rhod)opsin	Vertebrate blue/green opsin
Rh-009	Q90214	(Rhod)opsin	Vertebrate blue/green opsin
Rh-009	O62791	(Rhod)opsin	Vertebrate blue/green opsin
Rh-009	Q9YH00	(Rhod)opsin	Vertebrate blue/green opsin
Rh-009	O62796	(Rhod)opsin	Vertebrate blue/green opsin
Rh-009	Q90215	(Rhod)opsin	Vertebrate blue/green opsin
Rh-009	P79807	(Rhod)opsin	Vertebrate blue/green opsin
Rh-009	O62794	(Rhod)opsin	Vertebrate blue/green opsin
Rh-009	P79901	(Rhod)opsin	Vertebrate blue/green opsin
Rh-009	P79863	(Rhod)opsin	Vertebrate blue/green opsin
Rh-009	Q9YGZ5	(Rhod)opsin	Vertebrate blue/green opsin
Rh-009	Q9DGG4	(Rhod)opsin	Vertebrate blue/green opsin
Rh-009	Q28886	(Rhod)opsin	Vertebrate blue/green opsin
Rh-009	P79798	(Rhod)opsin	Vertebrate blue/green opsin
Rh-009	P08100	(Rhod)opsin	Vertebrate blue/green opsin
Rh-009	P56514	(Rhod)opsin	Vertebrate blue/green opsin
Rh-009	P41590	(Rhod)opsin	Vertebrate blue/green opsin
Rh-009	P79914	(Rhod)opsin	Vertebrate blue/green opsin
Rh-009	O13227	(Rhod)opsin	Vertebrate blue/green opsin
Rh-009	Q9YGY9	(Rhod)opsin	Vertebrate blue/green opsin
Rh-009	Q9YGZ3	(Rhod)opsin	Vertebrate blue/green opsin
Rh-009	Q9YGZ1	(Rhod)opsin	Vertebrate blue/green opsin
Rh-009	O62792	(Rhod)opsin	Vertebrate blue/green opsin
Rh-009	P49912	(Rhod)opsin	Vertebrate blue/green opsin
Rh-009	P51489	(Rhod)opsin	Vertebrate blue/green opsin
Rh-009	Q90245	(Rhod)opsin	Vertebrate blue/green opsin
Rh-009	Q769E8	(Rhod)opsin	Vertebrate blue/green opsin
Rh-009	Q9YH04	(Rhod)opsin	Vertebrate blue/green opsin
Rh-009	P79898	(Rhod)opsin	Vertebrate blue/green opsin
Rh-009	P79903	(Rhod)opsin	Vertebrate blue/green opsin
Rh-010	Q9W6A5	(Rhod)opsin	Vertebrate blue/green opsin

Rh-010	Q8AYM8	(Rhod)opsin	Vertebrate blue/green opsin
Rh-010	Q8AYM7	(Rhod)opsin	Vertebrate blue/green opsin
Rh-010	P87366	(Rhod)opsin	Vertebrate blue/green opsin
Rh-010	P32312	(Rhod)opsin	Vertebrate blue/green opsin
Rh-010	P51474	(Rhod)opsin	Vertebrate blue/green opsin
Rh-010	P51471	(Rhod)opsin	Vertebrate blue/green opsin
Rh-010	P35357	(Rhod)opsin	Vertebrate blue/green opsin
Rh-010	P28683	(Rhod)opsin	Vertebrate blue/green opsin
Rh-010	Q9W6A6	(Rhod)opsin	Vertebrate blue/green opsin
Rh-010	P32311	(Rhod)opsin	Vertebrate blue/green opsin
Rh-011	O57605	(Rhod)opsin	Vertebrate short wavelength opsin
Rh-011	P03999	(Rhod)opsin	Vertebrate short wavelength opsin
Rh-011	P51490	(Rhod)opsin	Vertebrate short wavelength opsin
Rh-011	P60573	(Rhod)opsin	Vertebrate short wavelength opsin
Rh-011	P51491	(Rhod)opsin	Vertebrate short wavelength opsin
Rh-011	Q63652	(Rhod)opsin	Vertebrate short wavelength opsin
Rh-011	P28684	(Rhod)opsin	Vertebrate short wavelength opsin
Rh-011	P51473	(Rhod)opsin	Vertebrate short wavelength opsin
Rh-011	O13092	(Rhod)opsin	Vertebrate short wavelength opsin
Rh-011	P60015	(Rhod)opsin	Vertebrate short wavelength opsin
Rh-012	O16019	(Rhod)opsin	Rhodopsin Arthropod short wavelength 2
Rh-012	O18486	(Rhod)opsin	Rhodopsin Arthropod short wavelength 2
Rh-012	O16018	(Rhod)opsin	Rhodopsin Arthropod short wavelength 2
Rh-012	O16017	(Rhod)opsin	Rhodopsin Arthropod short wavelength 2
Rh-012	P35356	(Rhod)opsin	Rhodopsin Arthropod short wavelength 2
Rh-012	O18312	(Rhod)opsin	Rhodopsin Arthropod short wavelength 2
Rh-012	O18315	(Rhod)opsin	Rhodopsin Arthropod short wavelength 2
Rh-012	O18481	(Rhod)opsin	Rhodopsin Arthropod short wavelength 2
Rh-012	O18485	(Rhod)opsin	Rhodopsin Arthropod short wavelength 2
Rh-012	O16020	(Rhod)opsin	Rhodopsin Arthropod short wavelength 2
Rh-013	Q17053	(Rhod)opsin	Rhodopsin Arthropod short wavelength 2
Rh-013	Q95YI3	(Rhod)opsin	Rhodopsin Arthropod short wavelength 2
Rh-013	P35362	(Rhod)opsin	Rhodopsin Arthropod short wavelength 2
Rh-013	O01668	(Rhod)opsin	Rhodopsin Arthropod short wavelength 2
Rh-013	Q17296	(Rhod)opsin	Rhodopsin Arthropod short wavelength 2
Rh-013	O02464	(Rhod)opsin	Rhodopsin Arthropod short wavelength 2
Rh-013	Q94741	(Rhod)opsin	Rhodopsin Arthropod short wavelength 2
Rh-014	O15973	(Rhod)opsin	Rhodopsin 6 1
Rh-014	O16005	(Rhod)opsin	Vertebrate opsin
Rh-014	Q17094	(Rhod)opsin	Vertebrate opsin
Rh-014	P31356	(Rhod)opsin	Vertebrate opsin
Rh-014	P24603	(Rhod)opsin	Vertebrate opsin
Rh-014	P09241	(Rhod)opsin	Vertebrate opsin
Rh-015	Q90309	(Rhod)opsin	Vertebrate short wavelength opsin
Rh-015	P87368	(Rhod)opsin	Vertebrate short wavelength opsin

Rh-015	Q9W6A9	(Rhod)opsin	Vertebrate short wavelength opsin
Rh-016	O42266	(Rhod)opsin	Vertebrate Parapinopsin
Rh-016	P51476	(Rhod)opsin	Vertebrate Pinopsin
Rh-016	P51475	(Rhod)opsin	Vertebrate Pinopsin
Rh-017	O14718	(Rhod)opsin	Vertebrate Peropsin
Rh-017	O35214	(Rhod)opsin	Vertebrate Peropsin
Rh-018	Q804X9	(Rhod)opsin	Vertebrate melanopsin
Rh-018	Q804Q2	(Rhod)opsin	Vertebrate melanopsin
Rh-019	Q4U4D2	(Rhod)opsin	Vertebrate melanopsin
Rh-019	O57422	(Rhod)opsin	Vertebrate melanopsin
Rh-020	P28678	(Rhod)opsin	Rhodopsin Arthropod short wavelength 2
Rh-020	P06002	(Rhod)opsin	Rhodopsin Arthropod short wavelength 2
Rh-020	P22269	(Rhod)opsin	Rhodopsin Arthropod short wavelength 2
Rh-021	P35361	(Rhod)opsin	Rhodopsin Arthropod short wavelength 2
Rh-021	P35360	(Rhod)opsin	Rhodopsin Arthropod short wavelength 2
Rh-022	P28679	(Rhod)opsin	Rhodopsin Arthropod short wavelength 2
Rh-022	P08099	(Rhod)opsin	Rhodopsin Arthropod short wavelength 2
Rh-023	Q6U736	(Rhod)opsin	Vertebrate opsin 5
Rh-023	Q6VZZ7	(Rhod)opsin	Vertebrate opsin 5
Rh-024	Q9H1Y3	(Rhod)opsin	Vertebrate opsin type 3
Rh-024	Q9WUK7	(Rhod)opsin	Vertebrate opsin type 3
Rh-025	Q1JPS6	(Rhod)opsin	Vertebrate melanopsin
Rh-025	Q6XL69	(Rhod)opsin	Vertebrate melanopsin
Rh-026	Q25157	(Rhod)opsin	Rhodopsin 5 3
Rh-026	Q25158	(Rhod)opsin	Rhodopsin 5 3
T2R-001	P59539	Taste receptors T2R	Taste receptors T2R
T2R-001	P59540	Taste receptors T2R	Taste receptors T2R
T2R-001	Q646G0	Taste receptors T2R	Taste receptors T2R
T2R-001	Q646F9	Taste receptors T2R	Taste receptors T2R
T2R-001	Q646E1	Taste receptors T2R	Taste receptors T2R
T2R-001	Q645Z7	Taste receptors T2R	Taste receptors T2R
T2R-001	Q645T4	Taste receptors T2R	Taste receptors T2R
T2R-001	Q5Y4Z8	Taste receptors T2R	Taste receptors T2R
T2R-001	Q5Y4Y8	Taste receptors T2R	Taste receptors T2R
T2R-002	Q7RTR8	Taste receptors T2R	Taste receptors T2R
T2R-002	Q646G3	Taste receptors T2R	Taste receptors T2R
T2R-002	Q646B8	Taste receptors T2R	Taste receptors T2R
T2R-002	Q645U9	Taste receptors T2R	Taste receptors T2R
T2R-002	Q5Y4Z2	Taste receptors T2R	Taste receptors T2R
T2R-002	Q645Z3	Taste receptors T2R	Taste receptors T2R
T2R-003	Q7M707	Taste receptors T2R	Taste receptors T2R
T2R-003	Q7M715	Taste receptors T2R	Taste receptors T2R
T2R-003	Q675B9	Taste receptors T2R	Taste receptors T2R
T2R-003	Q675B8	Taste receptors T2R	Taste receptors T2R
T2R-004	Q9NYW5	Taste receptors T2R	Taste receptors T2R

T2R-004	Q646F1	Taste receptors T2R	Taste receptors T2R
T2R-004	Q645Y8	Taste receptors T2R	Taste receptors T2R
T2R-004	Q646D3	Taste receptors T2R	Taste receptors T2R
T2R-005	Q7TQB0	Taste receptors T2R	Taste receptors T2R
T2R-005	Q67ES7	Taste receptors T2R	Taste receptors T2R
T2R-005	Q5Y4Z0	Taste receptors T2R	Taste receptors T2R
T2R-006	P59538	Taste receptors T2R	Taste receptors T2R
T2R-006	Q646B9	Taste receptors T2R	Taste receptors T2R
T2R-006	Q645V3	Taste receptors T2R	Taste receptors T2R
T2R-007	Q9NYW0	Taste receptors T2R	Taste receptors T2R
T2R-007	Q646F5	Taste receptors T2R	Taste receptors T2R
T2R-007	Q646B5	Taste receptors T2R	Taste receptors T2R
T2R-007	Q646D7	Taste receptors T2R	Taste receptors T2R
T2R-007	Q645V0	Taste receptors T2R	Taste receptors T2R
T2R-008	Q9NYV7	Taste receptors T2R	Taste receptors T2R
T2R-008	Q646B3	Taste receptors T2R	Taste receptors T2R
T2R-008	Q646E7	Taste receptors T2R	Taste receptors T2R
T2R-008	Q646D1	Taste receptors T2R	Taste receptors T2R
T2R-009	Q9NYV9	Taste receptors T2R	Taste receptors T2R
T2R-009	Q646B6	Taste receptors T2R	Taste receptors T2R
T2R-009	Q646D8	Taste receptors T2R	Taste receptors T2R
T2R-009	Q645V1	Taste receptors T2R	Taste receptors T2R
T2R-010	Q9NYW7	Taste receptors T2R	Taste receptors T2R
T2R-010	Q8MJU6	Taste receptors T2R	Taste receptors T2R
T2R-010	Q646G9	Taste receptors T2R	Taste receptors T2R
T2R-010	Q646H0	Taste receptors T2R	Taste receptors T2R
T2R-011	P59542	Taste receptors T2R	Taste receptors T2R
T2R-011	Q645Z9	Taste receptors T2R	Taste receptors T2R
T2R-011	Q5Y4Z5	Taste receptors T2R	Taste receptors T2R
T2R-011	Q5Y4Y9	Taste receptors T2R	Taste receptors T2R
T2R-012	Q646A5	Taste receptors T2R	Taste receptors T2R
T2R-012	Q645U7	Taste receptors T2R	Taste receptors T2R
T2R-012	Q646D0	Taste receptors T2R	Taste receptors T2R
T2R-013	Q9NYV8	Taste receptors T2R	Taste receptors T2R
T2R-013	Q646D9	Taste receptors T2R	Taste receptors T2R
T2R-013	Q645V2	Taste receptors T2R	Taste receptors T2R
T2R-014	Q9NYW2	Taste receptors T2R	Taste receptors T2R
T2R-014	Q646A3	Taste receptors T2R	Taste receptors T2R
T2R-014	Q645U8	Taste receptors T2R	Taste receptors T2R
T2R-015	Q7M713	Taste receptors T2R	Taste receptors T2R
T2R-015	Q67ER8	Taste receptors T2R	Taste receptors T2R
T2R-016	Q7TQA8	Taste receptors T2R	Taste receptors T2R
T2R-016	Q675B7	Taste receptors T2R	Taste receptors T2R
T2R-017	Q7M710	Taste receptors T2R	Taste receptors T2R
T2R-017	Q67ET4	Taste receptors T2R	Taste receptors T2R

T2R-018	Q9JKA3	Taste receptors T2R	Taste receptors T2R
T2R-018	Q67ET5	Taste receptors T2R	Taste receptors T2R
T2R-019	Q7TQA4	Taste receptors T2R	Taste receptors T2R
T2R-019	Q67ES0	Taste receptors T2R	Taste receptors T2R
T2R-020	Q7M711	Taste receptors T2R	Taste receptors T2R
T2R-020	Q67ES1	Taste receptors T2R	Taste receptors T2R
T2R-021	Q7M709	Taste receptors T2R	Taste receptors T2R
T2R-021	Q67ES6	Taste receptors T2R	Taste receptors T2R
T2R-022	Q7M724	Taste receptors T2R	Taste receptors T2R
T2R-022	Q67ET1	Taste receptors T2R	Taste receptors T2R
T2R-023	Q7M721	Taste receptors T2R	Taste receptors T2R
T2R-023	Q67ET3	Taste receptors T2R	Taste receptors T2R
T2R-024	Q7M717	Taste receptors T2R	Taste receptors T2R
T2R-024	Q675C0	Taste receptors T2R	Taste receptors T2R
T2R-025	Q9JKT7	Taste receptors T2R	Taste receptors T2R
T2R-025	Q7M720	Taste receptors T2R	Taste receptors T2R
T2R-026	Q9JKT8	Taste receptors T2R	Taste receptors T2R
T2R-026	Q7M722	Taste receptors T2R	Taste receptors T2R
T2R-027	Q9JKT4	Taste receptors T2R	Taste receptors T2R
T2R-027	Q9JKT5	Taste receptors T2R	Taste receptors T2R
T2R-028	Q9JKF0	Taste receptors T2R	Taste receptors T2R
T2R-028	P59528	Taste receptors T2R	Taste receptors T2R
T2R-029	Q7TQA5	Taste receptors T2R	Taste receptors T2R
T2R-029	Q67ER9	Taste receptors T2R	Taste receptors T2R
T2R-030	Q7M718	Taste receptors T2R	Taste receptors T2R
T2R-030	Q67ES5	Taste receptors T2R	Taste receptors T2R
T2R-031	Q7M712	Taste receptors T2R	Taste receptors T2R
T2R-031	Q9JKE8	Taste receptors T2R	Taste receptors T2R
T2R-032	Q9JKT3	Taste receptors T2R	Taste receptors T2R
T2R-032	Q67ET0	Taste receptors T2R	Taste receptors T2R
T2R-033	Q7TQB9	Taste receptors T2R	Taste receptors T2R
T2R-033	Q67ES3	Taste receptors T2R	Taste receptors T2R
T2R-034	Q7TQA7	Taste receptors T2R	Taste receptors T2R
T2R-034	Q67ET7	Taste receptors T2R	Taste receptors T2R
T2R-035	Q7M725	Taste receptors T2R	Taste receptors T2R
T2R-035	Q9JKT9	Taste receptors T2R	Taste receptors T2R
T2R-036	Q7M723	Taste receptors T2R	Taste receptors T2R
T2R-036	Q67ES9	Taste receptors T2R	Taste receptors T2R
T2R-037	Q9NYW1	Taste receptors T2R	Taste receptors T2R
T2R-037	Q645T0	Taste receptors T2R	Taste receptors T2R
T2R-038	Q646F4	Taste receptors T2R	Taste receptors T2R
T2R-038	Q645T2	Taste receptors T2R	Taste receptors T2R
T2R-039	Q7TQB8	Taste receptors T2R	Taste receptors T2R
T2R-039	Q67ET2	Taste receptors T2R	Taste receptors T2R
T2R-040	Q9JKE7	Taste receptors T2R	Taste receptors T2R

T2R-040	P59532	Taste receptors T2R	Taste receptors T2R
T2R-041	Q9JKT2	Taste receptors T2R	Taste receptors T2R
T2R-041	Q9JKU1	Taste receptors T2R	Taste receptors T2R
T2R-042	P59533	Taste receptors T2R	Taste receptors T2R
T2R-042	Q697L6	Taste receptors T2R	Taste receptors T2R
T2R-042	Q697L5	Taste receptors T2R	Taste receptors T2R
T2R-042	Q697L4	Taste receptors T2R	Taste receptors T2R
T2R-042	Q697L2	Taste receptors T2R	Taste receptors T2R
T2R-042	Q646E9	Taste receptors T2R	Taste receptors T2R
T2R-042	Q697L3	Taste receptors T2R	Taste receptors T2R
T2R-043	Q9NYW3	Taste receptors T2R	Taste receptors T2R
T2R-043	Q646F6	Taste receptors T2R	Taste receptors T2R
T2R-043	Q646A2	Taste receptors T2R	Taste receptors T2R
T2R-043	Q645V8	Taste receptors T2R	Taste receptors T2R
T2R-043	Q645T7	Taste receptors T2R	Taste receptors T2R
T2R-043	Q646D6	Taste receptors T2R	Taste receptors T2R
T2R-044	Q9NYW4	Taste receptors T2R	Taste receptors T2R
T2R-044	Q646D5	Taste receptors T2R	Taste receptors T2R
T2R-044	Q646E6	Taste receptors T2R	Taste receptors T2R
T2R-044	Q645Z1	Taste receptors T2R	Taste receptors T2R
T2R-044	Q645U0	Taste receptors T2R	Taste receptors T2R
T2R-045	P59544	Taste receptors T2R	Taste receptors T2R
T2R-045	Q646C3	Taste receptors T2R	Taste receptors T2R
T2R-045	Q646E4	Taste receptors T2R	Taste receptors T2R
T2R-045	Q646A1	Taste receptors T2R	Taste receptors T2R
T2R-045	Q645V7	Taste receptors T2R	Taste receptors T2R
T2R-046	P59535	Taste receptors T2R	Taste receptors T2R
T2R-046	Q646B1	Taste receptors T2R	Taste receptors T2R
T2R-046	Q645Y9	Taste receptors T2R	Taste receptors T2R
T2R-046	Q645U5	Taste receptors T2R	Taste receptors T2R
T2R-046	Q646D4	Taste receptors T2R	Taste receptors T2R
T2R-047	Q646E2	Taste receptors T2R	Taste receptors T2R
T2R-047	Q646C1	Taste receptors T2R	Taste receptors T2R
T2R-047	Q645V4	Taste receptors T2R	Taste receptors T2R
T2R-047	P59541	Taste receptors T2R	Taste receptors T2R
T2R-048	P59534	Taste receptors T2R	Taste receptors T2R
T2R-048	Q646A9	Taste receptors T2R	Taste receptors T2R
T2R-048	Q646C8	Taste receptors T2R	Taste receptors T2R
T2R-048	Q645S5	Taste receptors T2R	Taste receptors T2R
T2R-049	Q9NYW6	Taste receptors T2R	Taste receptors T2R
T2R-049	Q645Y5	Taste receptors T2R	Taste receptors T2R
T2R-049	Q646A7	Taste receptors T2R	Taste receptors T2R
T2R-049	Q646D2	Taste receptors T2R	Taste receptors T2R
T2R-050	P59536	Taste receptors T2R	Taste receptors T2R
T2R-050	Q646B2	Taste receptors T2R	Taste receptors T2R

T2R-050	Q646C7	Taste receptors T2R	Taste receptors T2R
T2R-051	P59543	Taste receptors T2R	Taste receptors T2R
T2R-051	Q646E3	Taste receptors T2R	Taste receptors T2R
T2R-051	Q646A0	Taste receptors T2R	Taste receptors T2R
T2R-052	P59537	Taste receptors T2R	Taste receptors T2R
T2R-052	Q646B4	Taste receptors T2R	Taste receptors T2R
T2R-052	Q5Y500	Taste receptors T2R	Taste receptors T2R
T2R-053	Q646F8	Taste receptors T2R	Taste receptors T2R
T2R-053	Q645T3	Taste receptors T2R	Taste receptors T2R
T2R-054	Q645T6	Taste receptors T2R	Taste receptors T2R
T2R-054	Q646G2	Taste receptors T2R	Taste receptors T2R
T2R-055	Q7TQA9	Taste receptors T2R	Taste receptors T2R
T2R-055	Q67ES2	Taste receptors T2R	Taste receptors T2R
T2R-056	Q7TQA6	Taste receptors T2R	Taste receptors T2R
T2R-056	Q4VHE7	Taste receptors T2R	Taste receptors T2R
T2R-057	P59530	Taste receptors T2R	Taste receptors T2R
T2R-057	Q9JKE9	Taste receptors T2R	Taste receptors T2R
Th-001	O08725	Thyrotropin-releasing hormone and Secretagogue	Growth hormone secretagogue
Th-001	Q99P50	Thyrotropin-releasing hormone and Secretagogue	Growth hormone secretagogue
Th-001	A5A4L1	Thyrotropin-releasing hormone and Secretagogue	Growth hormone secretagogue
Th-001	A5A4K9	Thyrotropin-releasing hormone and Secretagogue	Growth hormone secretagogue
Th-001	Q92847	Thyrotropin-releasing hormone and Secretagogue	Growth hormone secretagogue
Th-001	Q95254	Thyrotropin-releasing hormone and Secretagogue	Growth hormone secretagogue
Th-001	O43193	Thyrotropin-releasing hormone and Secretagogue	Growth hormone secretagogue like
Th-002	P21761	Thyrotropin-releasing hormone and Secretagogue	Thyrotropin-releasing hormone
Th-002	Q01717	Thyrotropin-releasing hormone and Secretagogue	Thyrotropin-releasing hormone
Th-002	O46639	Thyrotropin-releasing hormone and Secretagogue	Thyrotropin-releasing hormone
Th-002	P34981	Thyrotropin-releasing hormone and Secretagogue	Thyrotropin-releasing hormone
Th-002	O93603	Thyrotropin-releasing hormone and Secretagogue	Thyrotropin-releasing hormone
Th-002	Q28596	Thyrotropin-releasing hormone and Secretagogue	Thyrotropin-releasing hormone
Vi-001	P69332	Viral	US28
Vi-001	P69333	Viral	US28
outlier	O15974	(Rhod)opsin	G0 coupled Rhodopsin Mollusc
outlier	P22147	Amine	Alpha Adrenoceptors type 1d
outlier	O77408	Amine	Alpha Adrenoceptors type 2a
outlier	O01670	Amine	Alpha Adrenoceptors type 2c
outlier	P41596	Amine	Dopamine Insect type 1
outlier	Q24563	Amine	Dopamine Insect type 2

outlier	Q8IS44	Amine	Dopamine Vertebrate type 2
outlier	Q09388	Amine	Musc. acetylcholine Non Vertebrate
outlier	Q18007	Amine	Musc. acetylcholine Non Vertebrate
outlier	P16395	Amine	Musc. acetylcholine Non Vertebrate 2
outlier	Q19084	Amine	Serotonin type 1a
outlier	P20905	Amine	Serotonin type 1a
outlier	Q18775	Amine	Serotonin type 1a
outlier	Q8CGM1	Brain-specific angiogenesis inhibitor (BAI)	Brain-specific angiogenesis inhibitor (BAI) type 2
outlier	Q5R7Y0	Brain-specific angiogenesis inhibitor (BAI)	Brain-specific angiogenesis inhibitor (BAI) type 2
outlier	O60241	Brain-specific angiogenesis inhibitor (BAI)	Brain-specific angiogenesis inhibitor (BAI) type 2
outlier	Q80ZF8	Brain-specific angiogenesis inhibitor (BAI)	Brain-specific angiogenesis inhibitor (BAI) type 3
outlier	O60242	Brain-specific angiogenesis inhibitor (BAI)	Brain-specific angiogenesis inhibitor (BAI) type 3
outlier	Q9R0M0	Cadherin EGF LAG	Cadherin EGF LAG (CELSR)
outlier	Q9NYQ6	Cadherin EGF LAG	Cadherin EGF LAG (CELSR)
outlier	Q9QYP2	Cadherin EGF LAG	Cadherin EGF LAG (CELSR)
outlier	Q91ZI0	Cadherin EGF LAG	Cadherin EGF LAG (CELSR)
outlier	O88278	Cadherin EGF LAG	Cadherin EGF LAG (CELSR)
outlier	Q9NYQ7	Cadherin EGF LAG	Cadherin EGF LAG (CELSR)
outlier	Q9V5N8	Cadherin EGF LAG	Cadherin EGF LAG (CELSR)
outlier	Q9HCU4	Cadherin EGF LAG	Cadherin EGF LAG (CELSR)
outlier	O35161	Cadherin EGF LAG	Cadherin EGF LAG (CELSR)
outlier	Q9IB86	Calcitonin	Calcitonin
outlier	Q68EK2	Calcitonin	Calcitonin
outlier	Q8AXU4	Calcitonin	Calcitonin
outlier	Q7ZXS8	Calcitonin	Calcitonin
outlier	Q9R1W5	Calcitonin	Calcitonin
outlier	Q8WN93	Calcitonin	Calcitonin
outlier	Q63118	Calcitonin	Calcitonin
outlier	Q16602	Calcitonin	Calcitonin
outlier	A6QP74	Calcitonin	Calcitonin
outlier	Q0P4Y4	Calcitonin	Calcitonin
outlier	P25117	Calcitonin	Calcitonin
outlier	Q60755	Calcitonin	Calcitonin
outlier	P30988	Calcitonin	Calcitonin
outlier	O08893	Calcitonin	Calcitonin
outlier	P79222	Calcitonin	Calcitonin
outlier	P32214	Calcitonin	Calcitonin
outlier	Q09460	Calcitonin	Calcitonin
outlier	Q9QY96	Calcium sensing	Calcium sensing 1
outlier	P35384	Calcium sensing	Calcium sensing 1
outlier	P41180	Calcium sensing	Calcium sensing 1
outlier	P48442	Calcium sensing	Calcium sensing 1

outlier	O70410	Calcium sensing	Calcium sensing 2
outlier	P13773	cAMP receptors	cAMP receptors
outlier	P35352	cAMP receptors	cAMP receptors
outlier	P34907	cAMP receptors	cAMP receptors
outlier	Q9TX43	cAMP receptors	cAMP receptors
outlier	Q8ITC7	CAPA	CAPA
outlier	P48960	CD97	CD97
outlier	Q9Z0M6	CD97	CD97
outlier	Q8SQA4	CD97	CD97
outlier	Q9J529	Class A Orphan/other	EBV-induced
outlier	Q96CH1	Class A Orphan/other	GPR139
outlier	Q9DDD1	Class A Orphan/other	GPR139
outlier	Q8TDV2	Class A Orphan/other	GPR139
outlier	Q8N684	Class A Orphan/other	GPR88
outlier	Q7Z2Q7	Class A Orphan/other	LGR like (hormone receptors) type 7 and 8
outlier	P35410	Class A Orphan/other	Mas proto-oncogene and Mas-related (MRGs)
outlier	Q9Y2T6	Class A Orphan/other	Other 519
outlier	Q99PG6	Class C Other	Other 15
outlier	Q9Z0R8	Class C Other	Other 15
outlier	Q09630	Class C Other	Other 16
outlier	Q7RTX1	Class C Other	Other 25
outlier	Q9PW88	Class C Other	Other 5
outlier	Q8K4Z6	Class C Other	Other 6
outlier	Q70VB1	Class C Other	Other 6
outlier	P91685	Class C Other	Other 8
outlier	P34998	Corticotropin releasing factor	Corticotropin releasing factor
outlier	O42602	Corticotropin releasing factor	Corticotropin releasing factor
outlier	Q60748	Corticotropin releasing factor	Corticotropin releasing factor
outlier	Q13324	Corticotropin releasing factor	Corticotropin releasing factor
outlier	P47866	Corticotropin releasing factor	Corticotropin releasing factor
outlier	O42603	Corticotropin releasing factor	Corticotropin releasing factor
outlier	Q16983	Diuretic hormone	Diuretic hormone
outlier	P35464	Diuretic hormone	Diuretic hormone
outlier	Q9UHX3	ERM1	ERM1
outlier	Q2Q426	ERM1	ERM1
outlier	Q2Q421	ERM1	ERM1
outlier	Q9BY15	ERM1	ERM1
outlier	Q86SQ3	ERM1	ERM1
outlier	Q91ZE5	ERM1	ERM1
outlier	Q61549	ERM1	ERM1
outlier	Q5Y4N8	ERM1	ERM1
outlier	Q14246	ERM1	ERM1
outlier	Q9WV18	GABA-B	GABA-B subtype 1
outlier	Q9UBS5	GABA-B	GABA-B subtype 1
outlier	Q9Z0U4	GABA-B	GABA-B subtype 1

outlier	O75899	GABA-B	GABA-B subtype 2
outlier	O88871	GABA-B	GABA-B subtype 2
outlier	Q80T41	GABA-B	GABA-B subtype 2
outlier	Q0P543	Gastric inhibitory peptide	Gastric inhibitory peptide
outlier	P48546	Gastric inhibitory peptide	Gastric inhibitory peptide
outlier	P43218	Gastric inhibitory peptide	Gastric inhibitory peptide
outlier	P43219	Gastric inhibitory peptide	Gastric inhibitory peptide
outlier	O35659	Glucagon	Glucagon
outlier	P32301	Glucagon	Glucagon
outlier	P43220	Glucagon	Glucagon
outlier	Q5IXF8	Glucagon	Glucagon
outlier	O95838	Glucagon	Glucagon
outlier	Q9Z0W0	Glucagon	Glucagon
outlier	Q61606	Glucagon	Glucagon
outlier	P47871	Glucagon	Glucagon
outlier	P30082	Glucagon	Glucagon
outlier	Q8VEC3	GPR110	GPR110
outlier	Q5T601	GPR110	GPR110
outlier	Q8IZF5	GPR113	GPR113
outlier	Q58Y75	GPR113	GPR113
outlier	Q3V3Z3	GPR114	GPR114
outlier	Q8IZF4	GPR114	GPR114
outlier	Q7Z7M1	GPR114	GPR114
outlier	Q86SQ6	GPR123	GPR123
outlier	Q8IWK6	GPR124	GPR124
outlier	Q7TT36	GPR124	GPR124
outlier	Q96PE1	GPR124	GPR124
outlier	Q91ZV8	GPR124	GPR124
outlier	Q6F3F9	GPR126	GPR126
outlier	Q86SQ4	GPR126	GPR126
outlier	C6KFA3	GPR126	GPR126
outlier	B7ZCC9	GPR126	GPR126
outlier	Q8BM96	GPR128	GPR128
outlier	Q96K78	GPR128	GPR128
outlier	Q80T32	GPR133	GPR133
outlier	A6QLU6	GPR133	GPR133
outlier	Q8K209	GPR56	GPR56
outlier	Q9Y653	GPR56	GPR56
outlier	Q8K3V3	GPR56	GPR56
outlier	Q50DM8	GPR56	GPR56
outlier	Q50DM7	GPR56	GPR56
outlier	Q50DM6	GPR56	GPR56
outlier	Q50DM5	GPR56	GPR56
outlier	Q8CJ12	GPR64	GPR64
outlier	Q8CJ11	GPR64	GPR64

outlier	Q8IZP9	GPR64	GPR64
outlier	Q8IZF6	GPR64	GPR64
outlier	Q86Y34	GPR97	GPR97
outlier	Q8R0T6	GPR97	GPR97
outlier	P32082	Growth hormone-releasing hormone	Growth hormone-releasing hormone
outlier	Q02643	Growth hormone-releasing hormone	Growth hormone-releasing hormone
outlier	P34999	Growth hormone-releasing hormone	Growth hormone-releasing hormone
outlier	Q02644	Growth hormone-releasing hormone	Growth hormone-releasing hormone
outlier	Q80TR1	Latrophilin	Latrophilin
outlier	O88917	Latrophilin	Latrophilin
outlier	O94910	Latrophilin	Latrophilin
outlier	O97831	Latrophilin	Latrophilin
outlier	O97817	Latrophilin	Latrophilin
outlier	O88923	Latrophilin	Latrophilin
outlier	O95490	Latrophilin	Latrophilin
outlier	B3MFV7	Latrophilin	Latrophilin
outlier	B3N8M1	Latrophilin	Latrophilin
outlier	B4J780	Latrophilin	Latrophilin
outlier	B4KMZ1	Latrophilin	Latrophilin
outlier	B4GD14	Latrophilin	Latrophilin
outlier	B4LNA8	Latrophilin	Latrophilin
outlier	B4P3A0	Latrophilin	Latrophilin
outlier	Q292N4	Latrophilin	Latrophilin
outlier	A1Z7G7	Latrophilin	Latrophilin
outlier	B4HS00	Latrophilin	Latrophilin
outlier	Q9ESC1	Latrophilin	Latrophilin
outlier	Q923X1	Latrophilin	Latrophilin
outlier	Q9HBW9	Latrophilin	Latrophilin
outlier	P97772	Metabotropic glutamate	Metabotropic glutamate type 1 3
outlier	P23385	Metabotropic glutamate	Metabotropic glutamate type 1 3
outlier	Q13255	Metabotropic glutamate	Metabotropic glutamate type 1 3
outlier	Q14BI2	Metabotropic glutamate	Metabotropic glutamate type 2 3
outlier	P31421	Metabotropic glutamate	Metabotropic glutamate type 2 3
outlier	Q14416	Metabotropic glutamate	Metabotropic glutamate type 2 3
outlier	Q9QYS2	Metabotropic glutamate	Metabotropic glutamate type 3 1
outlier	P31422	Metabotropic glutamate	Metabotropic glutamate type 3 1
outlier	Q14832	Metabotropic glutamate	Metabotropic glutamate type 3 1
outlier	Q5RAL3	Metabotropic glutamate	Metabotropic glutamate type 3 1
outlier	Q1ZZH1	Metabotropic glutamate	Metabotropic glutamate type 3 1
outlier	P41594	Metabotropic glutamate	Metabotropic glutamate type 5 2
outlier	P31424	Metabotropic glutamate	Metabotropic glutamate type 5 2
outlier	Q3UVX5	Metabotropic glutamate	Metabotropic glutamate type 5 2

outlier	P35349	Metabotropic glutamate	Metabotropic glutamate type 6
outlier	O15303	Metabotropic glutamate	Metabotropic glutamate type 6
outlier	Q863I4	Metabotropic glutamate	Metabotropic glutamate type 6
outlier	Q5NCH9	Metabotropic glutamate	Metabotropic glutamate type 6
outlier	Q14833	Metabotropic glutamate	Metabotropic glutamate type 8
outlier	Q14831	Metabotropic glutamate	Metabotropic glutamate type 8
outlier	P31423	Metabotropic glutamate	Metabotropic glutamate type 8
outlier	P35400	Metabotropic glutamate	Metabotropic glutamate type 8
outlier	O00222	Metabotropic glutamate	Metabotropic glutamate type 8
outlier	P70579	Metabotropic glutamate	Metabotropic glutamate type 8
outlier	P47743	Metabotropic glutamate	Metabotropic glutamate type 8
outlier	Q68ED2	Metabotropic glutamate	Metabotropic glutamate type 8
outlier	Q5RDQ8	Metabotropic glutamate	Metabotropic glutamate type 8
outlier	Q1ZZH0	Metabotropic glutamate	Metabotropic glutamate type 8
outlier	Q68EF4	Metabotropic glutamate	Metabotropic glutamate type 8
outlier	O97148	Methuselah-like proteins (MTH)	Methuselah-like proteins (MTH) type 1
outlier	P83118	Methuselah-like proteins (MTH)	Methuselah-like proteins (MTH) type 1
outlier	Q9VRN2	Methuselah-like proteins (MTH)	Methuselah-like proteins (MTH) type 1
outlier	Q9GT50	Methuselah-like proteins (MTH)	Methuselah-like proteins (MTH) type 1
outlier	P83120	Methuselah-like proteins (MTH)	Methuselah-like proteins (MTH) type 1
outlier	Q9VS77	Methuselah-like proteins (MTH)	Methuselah-like proteins (MTH) type 1
outlier	Q9VSE7	Methuselah-like proteins (MTH)	Methuselah-like proteins (MTH) type 1
outlier	P83119	Methuselah-like proteins (MTH)	Methuselah-like proteins (MTH) type 1
outlier	Q95NT6	Methuselah-like proteins (MTH)	Methuselah-like proteins (MTH) type 2
outlier	Q95NQ0	Methuselah-like proteins (MTH)	Methuselah-like proteins (MTH) type 2
outlier	Q9W0R5	Methuselah-like proteins (MTH)	Methuselah-like proteins (MTH) type 2
outlier	Q9V818	Methuselah-like proteins (MTH)	Methuselah-like proteins (MTH) type 3
outlier	Q9W0R6	Methuselah-like proteins (MTH)	Methuselah-like proteins (MTH) type 3
outlier	Q9V817	Methuselah-like proteins (MTH)	Methuselah-like proteins (MTH) type 4
outlier	Q9NQ55	Nucleotide-like	P2RY11
outlier	Q96G91	Nucleotide-like	P2RY11
outlier	Q8TDS5	Nucleotide-like	P2RY5
outlier	Q99ME2	Nucleotide-like	Purinoceptor P2RY12-14 GPR87 (UDP-Glucose)
outlier	Q5U9X3	Odorant	Odorant 4
outlier	Q5T6X5	Odorant	Odorant 5
outlier	Q8NGE0	Olfactory	Olfactory 184
outlier	Q8N148	Olfactory	Olfactory 216
outlier	Q9H342	Olfactory	Olfactory 243
outlier	Q8NGR6	Olfactory	Olfactory 257
outlier	A6NFC9	Olfactory	Olfactory 73
outlier	Q8NH09	Olfactory	Olfactory 85
outlier	Q8NH95	Olfactory	Olfactory 95
outlier	Q8NGJ8	Olfactory	Olfactory II fam 10 / MOR263-269
outlier	Q6IF63	Olfactory	Olfactory II fam 12 / MOR250
outlier	Q9GZK7	Olfactory	Olfactory II fam 3 / MOR255

outlier	Q8NG92	Olfactory	Olfactory II fam 3 / MOR255
outlier	A6NND4	Olfactory	Olfactory II fam 3 / MOR255
outlier	Q8NH08	Olfactory	Olfactory II fam 3 / MOR255
outlier	P70205	PACAP	PACAP
outlier	P41586	PACAP	PACAP
outlier	Q29627	PACAP	PACAP
outlier	P32215	PACAP	PACAP
outlier	P41593	Parathyroid hormone	Parathyroid hormone
outlier	Q03431	Parathyroid hormone	Parathyroid hormone
outlier	Q9TU31	Parathyroid hormone	Parathyroid hormone
outlier	P25107	Parathyroid hormone	Parathyroid hormone
outlier	P50133	Parathyroid hormone	Parathyroid hormone
outlier	P25961	Parathyroid hormone	Parathyroid hormone
outlier	Q5RAQ1	Parathyroid hormone	Parathyroid hormone
outlier	Q1LZF7	Parathyroid hormone	Parathyroid hormone
outlier	P49190	Parathyroid hormone	Parathyroid hormone
outlier	Q91V95	Parathyroid hormone	Parathyroid hormone
outlier	Q9W4Y2	PDF	PDF
outlier	Q8WPA2	Peptide	Allostatin
outlier	Q75W84	Peptide	Annetocin
outlier	Q6SW98	Peptide	C-C Chemokine type 11
outlier	Q66673	Peptide	C-C Chemokine type 3
outlier	P52380	Peptide	C-C Chemokine type 3
outlier	Q86917	Peptide	C-C Chemokine type 8
outlier	Q9J5H4	Peptide	Fmet-leu-phe
outlier	Q9J5I0	Peptide	Fmet-leu-phe
outlier	Q11082	Peptide	Galanin type 1
outlier	Q03613	Peptide	GPR74 like
outlier	Q09638	Peptide	GPR74 like
outlier	P34311	Peptide	GPR74 like
outlier	Q9NDM2	Peptide	Melanin-concentrating hormone receptors
outlier	Q9VZW5	Peptide	Neuropeptide Y / peptide YY
outlier	Q9VNM1	Peptide	Neuropeptide Y type 1
outlier	Q09502	Peptide	Prolactin-releasing peptide (GPR10)
outlier	O42179	Peptide	Somatostatin type 5
outlier	Q03566	Peptide	Substance P (NK1)
outlier	P25931	Peptide	Tachykinin like 2
outlier	Q5W9T5	Peptide	Vasotocin
outlier	Q7YW31	Peptide	Vasotocin
outlier	Q868T3	Peptide	Vasotocin
outlier	Q2V2K5	Peptide	Vasotocin
outlier	Q5WA50	Peptide	Vasotocin
outlier	P47872	Secretin	Secretin
outlier	O46502	Secretin	Secretin
outlier	P23811	Secretin	Secretin

outlier	Q5FWI2	Secretin	Secretin
outlier	Q717C2	Taste	Taste 1
outlier	Q717C1	Taste	Taste 1
outlier	Q7RTX0	Taste	Taste 1
outlier	Q925I4	Taste	Taste 2
outlier	Q9Z0R7	Taste	Taste 2
outlier	Q8TE23	Taste	Taste 2
outlier	Q49HI0	Taste	Taste 2
outlier	A3QNZ8	Taste	Taste 2
outlier	A3QNZ9	Taste	Taste 2
outlier	A3QP00	Taste	Taste 2
outlier	A3QP01	Taste	Taste 2
outlier	A3QP07	Taste	Taste 2
outlier	A3QP08	Taste	Taste 2
outlier	Q925D8	Taste	Taste 21
outlier	Q923K1	Taste	Taste 21
outlier	Q49KI5	Taste	Taste 22
outlier	Q49HH9	Taste	Taste 23
outlier	O62772	To be sorted	To be sorted
outlier	Q90812	To be sorted	To be sorted
outlier	P35347	To be sorted	To be sorted
outlier	P35353	To be sorted	To be sorted
outlier	Q76LL8	To be sorted	To be sorted
outlier	O97827	To be sorted	To be sorted
outlier	Q9Z173	To be sorted	To be sorted
outlier	Q9HAR2	To be sorted	To be sorted
outlier	Q80TS3	To be sorted	To be sorted
outlier	O14514	To be sorted	To be sorted
outlier	Q3UHD1	To be sorted	To be sorted
outlier	Q8IZF7	To be sorted	To be sorted
outlier	Q8IZF3	To be sorted	To be sorted
outlier	Q9D2L6	To be sorted	To be sorted
outlier	Q6QNK2	To be sorted	To be sorted
outlier	Q9WVT0	To be sorted	To be sorted
outlier	Q8IZF2	To be sorted	To be sorted
outlier	Q6JAN0	To be sorted	To be sorted
outlier	Q8VHN7	To be sorted	To be sorted
outlier	Q91085	Vasoactive intestinal polypeptide	Vasoactive intestinal polypeptide
outlier	Q90308	Vasoactive intestinal polypeptide	Vasoactive intestinal polypeptide
outlier	P97751	Vasoactive intestinal polypeptide	Vasoactive intestinal polypeptide
outlier	P32241	Vasoactive intestinal polypeptide	Vasoactive intestinal polypeptide
outlier	P30083	Vasoactive intestinal polypeptide	Vasoactive intestinal polypeptide
outlier	Q28992	Vasoactive intestinal polypeptide	Vasoactive intestinal polypeptide
outlier	P41588	Vasoactive intestinal polypeptide	Vasoactive intestinal polypeptide
outlier	P41587	Vasoactive intestinal polypeptide	Vasoactive intestinal polypeptide

outlier	P35000	Vasoactive intestinal polypeptide	Vasoactive intestinal polypeptide
outlier	P09703	Viral	US27
outlier	Q6TAC4	Vomeronasal	Vomeronasal 9
outlier	Q5J3F6	Vomeronasal receptors other	Vomeronasal receptors other
outlier	Q7Z5H4	Vomeronasal receptors other	Vomeronasal receptors other
outlier	Q7YRP1	Vomeronasal receptors other	Vomeronasal receptors other
outlier	Q9EQ48	Vomeronasal receptors V1RA	Vomeronasal receptors V1RA
outlier	Q9EP79	Vomeronasal receptors V1RA	Vomeronasal receptors V1RA
outlier	Q5J3G9	Vomeronasal receptors V1RA	Vomeronasal receptors V1RA
outlier	Q5J3E5	Vomeronasal receptors V1RA	Vomeronasal receptors V1RA
outlier	Q5J3M4	Vomeronasal receptors V1RA	Vomeronasal receptors V1RA
outlier	Q5J3L6	Vomeronasal receptors V1RA	Vomeronasal receptors V1RA
outlier	Q9EPB8	Vomeronasal receptors V1RA	Vomeronasal receptors V1RA
outlier	Q9EQ51	Vomeronasal receptors V1RA	Vomeronasal receptors V1RA
outlier	Q9EQ52	Vomeronasal receptors V1RA	Vomeronasal receptors V1RA
outlier	Q5J3K9	Vomeronasal receptors V1RA	Vomeronasal receptors V1RA
outlier	Q5J3K5	Vomeronasal receptors V1RA	Vomeronasal receptors V1RA
outlier	Q62850	Vomeronasal receptors V1RA	Vomeronasal receptors V1RA
outlier	Q8VIC7	Vomeronasal receptors V1RA	Vomeronasal receptors V1RA
outlier	Q8R2E6	Vomeronasal receptors V1RA	Vomeronasal receptors V1RA
outlier	Q8VIC6	Vomeronasal receptors V1RA	Vomeronasal receptors V1RA
outlier	Q8VIC9	Vomeronasal receptors V1RA	Vomeronasal receptors V1RA
outlier	Q8VBS7	Vomeronasal receptors V1RA	Vomeronasal receptors V1RA
outlier	Q9WUF1	Vomeronasal receptors V1RB	Vomeronasal receptors V1RB
outlier	Q9EQ45	Vomeronasal receptors V1RB	Vomeronasal receptors V1RB
outlier	Q9EP51	Vomeronasal receptors V1RB	Vomeronasal receptors V1RB
outlier	Q9EQ47	Vomeronasal receptors V1RB	Vomeronasal receptors V1RB
outlier	Q9EP93	Vomeronasal receptors V1RB	Vomeronasal receptors V1RB
outlier	Q9EQ46	Vomeronasal receptors V1RB	Vomeronasal receptors V1RB
outlier	Q9EQ44	Vomeronasal receptors V1RB	Vomeronasal receptors V1RB
outlier	Q5J3M9	Vomeronasal receptors V1RB	Vomeronasal receptors V1RB
outlier	Q5J3L7	Vomeronasal receptors V1RB	Vomeronasal receptors V1RB
outlier	Q5J3N1	Vomeronasal receptors V1RB	Vomeronasal receptors V1RB
outlier	Q5J3M3	Vomeronasal receptors V1RB	Vomeronasal receptors V1RB
outlier	Q5J3L4	Vomeronasal receptors V1RB	Vomeronasal receptors V1RB
outlier	Q7Z5H5	Vomeronasal receptors V1RF	Vomeronasal receptors V1RF
outlier	Q7YRP2	Vomeronasal receptors V1RF	Vomeronasal receptors V1RF
outlier	Q8NFZ6	Vomeronasal receptors V1RL	Vomeronasal receptors V1RL
outlier	Q9GZP7	Vomeronasal receptors V1RL	Vomeronasal receptors V1RL
outlier	Q8WN92	Vomeronasal receptors V1RL	Vomeronasal receptors V1RL
outlier	Q7YRP3	Vomeronasal receptors V1RL	Vomeronasal receptors V1RL
outlier	Q9BXE9	Vomeronasal receptors V1RL	Vomeronasal receptors V1RL

Table S2. GPCR detection of 54 membrane protein sequences in the test dataset. The three sequences in red are falsely predicted to be non-GPCRs, due to incomplete information in the target dataset.

Uniprot ID	Entry name	Minimum distance to the target dataset	GPCR annotation
Q8ITC9	PK1R_DROME	1.00E-53	GPCR
Q6IF36	O8G2P_HUMAN	8.00E-148	GPCR
P0DN80	OR5H8_HUMAN	1.00E-149	GPCR
P0DN82	O12D1_HUMAN	2.00E-152	GPCR
P0DMU2	OR83P_HUMAN	0	GPCR
P0DN81	O13C7_HUMAN	1.00E-154	GPCR
P0DMS8	AA3R_HUMAN	0	GPCR
O45767	SRX43_CAEEL	0.002	GPCR
G5ECD9	AEX2_CAEEL	4.00E-08	GPCR
P0DN77	OPSG2_HUMAN	0	GPCR
P0DN78	OPSG3_HUMAN	0	GPCR
G4WMX4	RYAR_TRICA	1.00E-94	GPCR
E9QJ73	CXR32_DANRE	9.00E-61	GPCR
O02300	NTR1_CAEEL	4.00E-35	GPCR
O62169	NTR2_CAEEL	2.00E-31	GPCR
Q9U320	DAF38_CAEEL	2.00E-23	GPCR
Q8SWR3	SPR_DROME	6.00E-20	GPCR
Q23497	NPR9_CAEEL	7.00E-59	GPCR
Q18321	DAF37_CAEEL	3.00E-35	GPCR
Q4V622	CCH2R_DROME	4.00E-57	GPCR
A1ZAX0	CCH1R_DROME	5.00E-55	GPCR
G5ECQ2	FRIZ2_CAEEL	0.022	GPCR
Q8C4G9	AGRA1_MOUSE	0	GPCR
Q8K4C8	FZD9_RAT	0.015	GPCR
D4A3T6	AGRF2_RAT	0	GPCR
E9Q4J9	AGRF2_MOUSE	0	GPCR
Q7JQF1	OAMB_DROME	3.00E-65	GPCR
Q8BGT1	FLRT3_MOUSE	5.00E-21	GPCR
Q9VML9	TRISR_DROME	6.00E-27	GPCR
Q7SY09	AGRL4_DANRE	0	GPCR
Q8IN35	SIFAR_DROME	2.00E-54	GPCR
S4X0Q8	AGRA3_DANRE	0	GPCR
G5E8Q8	AGRF5_MOUSE	0	GPCR
E7FBY6	AGRA2_DANRE	0	GPCR
Q6ZIV7	HIR1_ORYSJ	12	Non-GPCR
P0DOE6	MATRX_HRSS2	8.2	Non-GPCR
P0DOE7	MATRX_HRSVA	8.2	Non-GPCR
Q5QNI2	NCL1_ORYSJ	1	Non-GPCR

Q8L636	NCL_ARATH	0.48	Non-GPCR
Q8LD98	NHL6_ARATH	0.025	Non-GPCR
Q6K3R5	NCL2_ORYSJ	1.8	Non-GPCR
G3V7W1	PDCD6_RAT	2	Non-GPCR
A3PDP9	PHND2_PROM0	3.6	Non-GPCR
Q9AYU1	QR1_TRIVS	1.4	Non-GPCR
F1PCT7	RPN2_CANLF	0.83	Non-GPCR
E2RQ08	RPN1_CANLF	0.22	Non-GPCR
F4I3V6	SHW1_ARATH	0.91	Non-GPCR
Q9V3J4	SEC13_DROME	0.054	Non-GPCR
Q9FJ04	SPH26_ARATH	1.9	Non-GPCR
F1PJP5	STT3A_CANLF	1.6	Non-GPCR
B3H7A6	SPH19_ARATH	10	Non-GPCR
F2Q9V4	SPH6_ARATH	1.6	Non-GPCR
Q9FI83	SPH28_ARATH	1.2	Non-GPCR
Q8GUJ1	UXT2_ARATH	0.75	Non-GPCR
